# A neural model of normal and abnormal learning and memory consolidation: adaptively timed conditioning, hippocampus, amnesia, neurotrophins, and consciousness

**DOI:** 10.3758/s13415-016-0463-y

**Published:** 2016-11-30

**Authors:** Daniel J. Franklin, Stephen Grossberg

**Affiliations:** Center for Adaptive Systems, Graduate Program in Cognitive and Neural Systems, and Departments of Mathematics, Psychological & Brain Sciences, and Biomedical Engineering, Boston University, 677 Beacon Street, Room 213, Boston, MA 02215 USA

**Keywords:** **C**ognitive-emotional learning, Conditioning, Memory consolidation, Amnesia, Hippocampus, Amygdala, Pontine nuclei, Adaptive timing, Time cells, BDNF

## Abstract

How do the hippocampus and amygdala interact with thalamocortical systems to regulate cognitive and cognitive-emotional learning? Why do lesions of thalamus, amygdala, hippocampus, and cortex have differential effects depending on the phase of learning when they occur? In particular, why is the hippocampus typically needed for trace conditioning, but not delay conditioning, and what do the exceptions reveal? Why do amygdala lesions made before or immediately after training decelerate conditioning while those made later do not? Why do thalamic or sensory cortical lesions degrade trace conditioning more than delay conditioning? Why do hippocampal lesions during trace conditioning experiments degrade recent but not temporally remote learning? Why do orbitofrontal cortical lesions degrade temporally remote but not recent or post-lesion learning? How is temporally graded amnesia caused by ablation of prefrontal cortex after memory consolidation? How are attention and consciousness linked during conditioning? How do neurotrophins, notably brain-derived neurotrophic factor (BDNF), influence memory formation and consolidation? Is there a common output path for learned performance? A neural model proposes a unified answer to these questions that overcome problems of alternative memory models.

## Overview and scope

The roles and interactions of amygdala, hippocampus, thalamus, and neocortex in cognitive and cognitive-emotional learning, memory, and consciousness have been extensively investigated through experimental and clinical studies (Berger & Thompson, 1978; Clark, Manns, & Squire, 2001; Frankland & Bontempi, 2005; Kim, Clark, & Thompson, 1995; Lee & Kim, 2004 **;** Mauk & Thompson 1987; Moustafa et al., 2013; Port, Romano, Steinmetz, Mikhail, & Patterson, 1986; Powell & Churchwell, 2002; Smith, 1968; Takehara, Kawahara, & Krino, 2003). This article develops a neural model aimed at providing a unified explanation of challenging data about how these brain regions interact during normal learning, and how lesions may cause specific learning and behavioral deficits, including amnesia. The model also proposes testable predictions to further test its explanations. The most relevant experiments use the paradigm of classical conditioning, notably delay conditioning and trace conditioning during the eyeblink conditioning task that is often used to explicate basic properties of associative learning. Earlier versions of this work were briefly presented in Franklin and Grossberg (2005, 2008).

Eyeblink conditioning has been extensively studied because it has disclosed behavioral, neurophysiological, and anatomical information about the learning and memory processes related to adaptively timed, conditioned responses to aversive stimuli, as measured by eyelid movements in mice (Chen et al., 1995), rats (Clark, Broadbent, Zola, & Squire, 2002; Neufeld & Mintz, 2001; Schmajuk, Lam, & Christiansen, 1994), monkeys (Clark & Zola, 1998), and humans (Clark, Manns, & Squire, 2001; Solomon et al., 1990), and by the timing and amplitude of the nictitating membrane reflex (NMR) which involves a nictitating membrane that covers the eye like an eyelid in cats (Norman et al., 1974), rabbits (Berger & Thompson, 1978; Christian & Thompson, 2003; McLaughlin, Skaggs, Churchwell, & Powell, 2002; Port, Mikhail, & Patterson, 1985; Port et al., 1986; Powell & Churchill 2002; Powell, Skaggs, Churchwell, & McLauglin, 2001; Solomon et al., 1990), and other animals. Eyeblink/NMR conditioning data will herein be used to help formulate and answer basic questions about associative learning, adaptive timing, and memory consolidation.

**Fig. 1:**
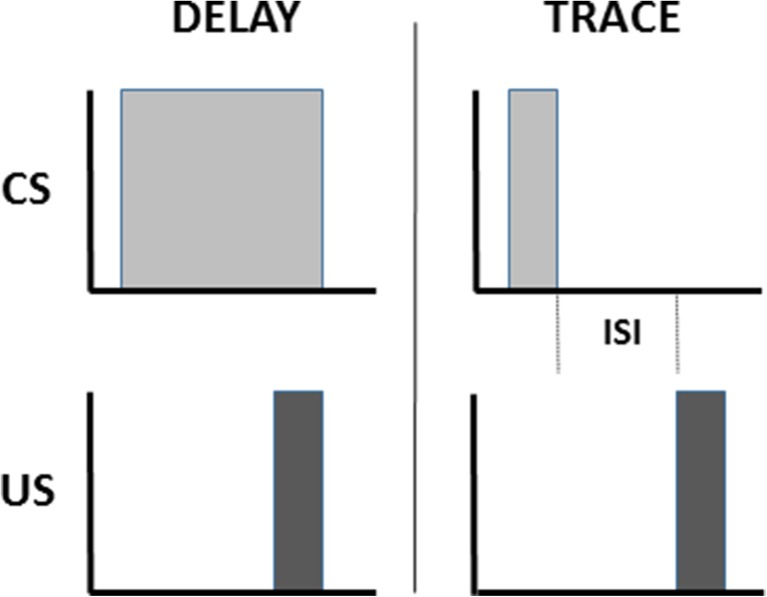
Eyeblink conditioning associates a neutral event, called the conditioned stimulus (CS), with an emotionally-charged, reflex-inducing event, called the unconditioned stimulus (US). Delay conditioning occurs when the stimulus events temporally overlap. Trace conditioning involves a temporal gap between CS offset and US onset such that a CS-activated memory trace is required during the inter-stimulus interval (ISI) in order to establish an association between CS and US. After either normal delay and trace conditioning, with a range of stimulus durations and ISIs a conditioned response (CR) is performed in anticipation of the US

Classical conditioning involves learning associations between objects or events. Eyeblink conditioning associates a neutral event, such as a tone or a light, called the *conditioned stimulus* (CS), with an emotionally-charged, reflex-inducing event, such as a puff of air to the eye or a shock to the periorbital area, called the *unconditioned stimulus* (US). *Delay conditioning* occurs when the stimulus events temporally overlap so that the subject learns to make a conditioned response (CR) in anticipation of the US (Fig. 1). *Trace conditioning* involves a temporal gap between CS offset and US onset such that a CS-activated memory trace is required during the inter-stimulus interval (ISI) in order to establish an adaptively timed association between CS and US that leads to a successful CR (Pavlov, 1927).

**Fig. 2:**
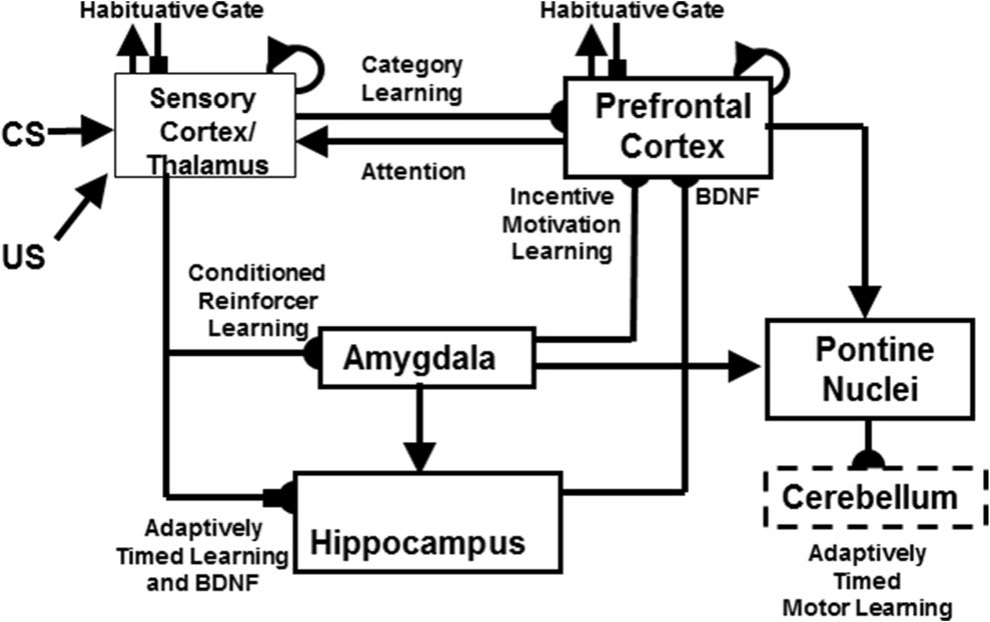
The neurotrophic START, or nSTART, macrocircuit is formed from parallel and interconencted networks that support both delay and trace conditioing. Connectivity between thalamus and sensory cortex includes pathways from the amygdala and hippocampus, as does connectivity between sensory cortex and prefrontal cortex, specifically orbitofrontal cortex. These circuits are homologous. Hence the current model lumps the thalamus and sensory cortex together and simulates only sensory cortical dynamics. Multiple types of learning and neurotrophic mechanisms of memory consolidation cooperate in these circuits to generate adaptively timed responses. Connections from sensory cortex to orbitofrontal cortex support category learning. Reciprocal connections from orbitofrontal cortex to sensory cortex support attention. Habituative transmitter gates modulate excitatory conductances at all processing stages. Connections from sensory cortex to amygdala connections support conditioned reinforcer learning. Connections from amygdala to orbitofrontal cortex support incentive motivation learning. Hippocampal adaptive timing and brain-derived neurotrophic factor (BDNF) bridge temporal delays between conditioned stimulus (CS) offset and unconditioned stimulus (US) onset during trace conditioning acquisition. BDNF also supports long-term memory consolidation within sensory cortex to hippocampal pathways and from hippocampal to orbitofrontal pathways. The pontine nuclei serve as a final common pathway for reading-out conditioned responses. Cerebellar dynamics are not simulated in nSTART. Key: arrowhead = excitatory synapse; hemidisc = adaptive weight; square = habituative transmitter gate; square followed by a hemidisc = habituative transmitter gate followed by an adaptive weight

Multiple brain areas are involved in eyeblink conditioning. Many of these regions, and their interactions, are simulated in the current neural model (Fig. 2). Sensory input comes into the cortex, and the model, by way of the thalamus. Since the US is an aversive stimulus, the amygdala is involved (Büchel, Dolan, Armony, & Friston, 1999; Lee & Kim, 2004). The hippocampus plays a role in new learning, in general (Frankland & Bontempi, 2005; Kim, Clark, & Thompson, 1995; Takehara et al., 2003) and in adaptively timed learning, in particular (Büchel et al., 1999; Green & Woodruff-Pak, 2000; Kaneko & Thompson, 1997; Port et al., 1986; Smith, 1968). The prefrontal cortex plays an essential role in the consolidation of long-term memory (Frankland & Bontempi, 2005; Takehara, Kawahara, & Krino, 2003; Winocur, Moscovitch, & Bontempi, 2010). Lesions of the amygdala, hippocampus, thalamus, and neocortex have different effects depending on the phase of learning when they occur.

In particular, the model clarifies why the hippocampus is needed for trace conditioning, but not delay conditioning (Büchel et al., 1999; Frankland & Bontempi, 2005; Green & Woodruff-Pak, 2000; Kaneko & Thompson, 1997; Kim, Clark, & Thompson, 1995; Port et al., 1986; Takehara, Kawahara, & Krino, 2003); why thalamic lesions retard the acquisition of trace conditioning (Powell & Churchwell, 2002), but have less of a statistically significant effect on delay conditioning (Buchanan & Thompson, 1990); why early but not late amygdala lesions degrade both delay conditioning (Lee & Kim, 2004) and trace conditioning (Büchel et al., 1999); why hippocampal lesions degrade recent but not temporally remote trace conditioning (Kim et al., 1995; Takehara et al., 2003); why in delay conditioning, such lesions typically have no negative impact on CR performance but this finding may vary with experimental preparation and CR success criteria (Berger, 1984; Chen et al., 1995; Lee & Kim, 2004; Port, 1985; Shors, 1992; Moustafa, et al., 2013); why cortical lesions degrade temporally remote but not recent trace conditioning, but have no impact on the acquisition of delay conditioning (Frankland & Bontempi, 2005; Kronforst-Collins & Disterhoft, 1998; McLaughlin et al., 2002; Takehara et al., 2003; see also, Oakley & Steele Russell, 1972; Yeo, Hardiman, Moore, & Steele Russell,^.^ 1984); how temporally-graded amnesia may be caused by ablation of the medial prefrontal cortex after memory consolidation (Simon, Knuckley, Churchwell, & Powell, 2005; Takehara et al., 2003; Weible, McEchron, & Disterhoft, 2000); how attention and consciousness are linked during delay and trace conditioning (Clark, Manns, & Squire, 2002; Clark & Squire, 1998, 2010); and how neurotrophins, notably brain-derived neurotrophic factor (BDNF), influence memory formation and consolidation (Kokaia et al., 1993, Tyler et al., 2002).

The article does not attempt to explain all aspects of memory consolidation, although its proposed explanations may help to do so in future studies. One reason for this is that the prefrontal cortex and hippocampus, which figure prominently in model explanations, carry out multiple functions (see section ‘*Clinical relevance of BDNF*). The model only attempts to explain how an interacting subset of these mechanisms contribute to conditioning and memory consolidation. Not considered, for example, are sequence-dependent learning, which depends on prefrontal working memories and list chunking dynamics (cf. compatible models for such processes in Grossberg & Kazerounian, 2016; Grossberg & Pearson, 2008; and Silver et al., 2011), or spatial navigation, which depends upon entorhinal grid cells and hippocampal place cells (cf. compatible models in Grossberg & Pilly, 2014; Pilly & Grossberg, 2012). In addition, the model does not attempt to simulate properties such as hippocampal replay, which require an analysis of sequence-dependent learning, including spatial navigation, for their consideration, or finer neurophysiological properties such the role of sleep, sharp wave ripples, and spindles in memory consolidation (see Albouy, King, Maquet, & Doyon, 2013, for a review).

Data about brain activity during sleep provide further evidence about learning processes that support memory consolidation. These processes begin with awake experience and may continue during sleep where there are no external stimuli that support learning (Kali & Dayan, 2004; Wilson, 2002). The activity generated during waking in the hippocampus is reproduced in sequence during rapid eye movement (REM) sleep with the same time scale as the original experiences, lasting tens of seconds to minutes (Louie & Wilson, 2001), or is compressed during slow-wave sleep (Nádasdy et al. 1999). During sleep, slow waves appear to be initiated in hippocampal CA3 (Siapas, Lubenov, & Wilson, 2005; Wilson & McNaughton, 1994), and hippocampal place cells tend to fire as though neuronal states were being played back in their previously experienced sequence as part of the memory consolidation process (Ji & Wilson, 2007; Qin, McNaughton, Skaggs, & Barnes, 1997; Skaggs & McNaughton, 1996; Steriade, 1999; Wilson & McNaughton, 1994). Relevant to the nSTART analysis are the facts that, during sleep, the interaction of hippocampal cells with cortex leads to neurotrophic expression (Hobson & Pace-Schott, 2002; Monteggia et al., 2004), and that similar sequential, self-organizing ensembles that are based on experience may also exist in various areas of the neocortex (Ji & Wilson, 2007; Maquet et al., 2000; cf. Deadwyler, West, & Robinson, 1981; Schoenbaum & Eichenbaum, 1995). With the nSTART analyses of neurotrophically-modulated memory consolidation as a function, these sleep- and sequence-dependent processes, which require substantial additional model development, can be more easily understood.

## Unifying three basic competences

The model reconciles three basic behavioral competences. Its explanatory power is illustrated by the fact that these basic competences are self-evident, but the above data properties are not. All three competences involve the brain’s ability to *adaptively time* its learning processes in a task-appropriate manner.

First, the brain needs to pay attention quickly to salient events, both positive and negative. However, such a rapid attention shift to focus on a salient event creates the risk of prematurely responding to that event, or of prematurely resetting and shifting the attentional focus to a different event before the response to that event could be fully executed. As explained below, this fast motivated attention pathway includes the amygdala. These potential problems of a fast motivated attention shift are alleviated by the second and third competences.

Second, the brain needs to be able to adaptively time and maintain motivated attention on a salient event until an appropriate response is executed. The ability to maintain motivated attention for an adaptively timed interval on the salient event involves the hippocampus, notably its dentate-CA3 region (Berger, Clark, & Thompson, 1980). Recent data have further developed this theme through the discovery of hippocampal “time cells” (Kraus et al., 2013; MacDonald et al., 2011).

Third, the brain needs to be able to adaptively time and execute an appropriate response to the salient event. The ability to execute an adaptively timed behavioral response always involves the cerebellum (Christian & Thompson, 2003; Fiala, Grossberg, & Bullock, 1996; Green & Woodruff-Pak, 2000; Ito, 1984). When the timing contingencies involve a relatively long trace conditioning ISI, or the onset of the US in delay conditioning is sufficiently delayed, then the hippocampus may also be required due to higher cognitive demand (Beylin, Gandhi, Wood, Talk, Matzel, & Shors, 2001).

How the brain may realize these three competences, along with data supporting these hypotheses, has been described in articles about the Spectrally Timed Adaptive Resonance Theory (START) model of Grossberg & Merrill (1992, 1996). A variation of the START model in which several of its mechanisms are out of balance is called the Imbalanced START, or iSTART, model that has been used to describe possible neural mechanisms of autism (Grossberg & Seidman, 2006). START mechanisms have also been used to offer mechanistic explanations of various symptoms of schizophrenia (Grossberg, 2000b). The current *neurotrophic START*, or nSTART, model builds upon this foundation. The nSTART model further develops the START model to refine the anatomical interactions that are described in START, to clarify how adaptively timed learning and memory consolidation depend upon neurotrophins acting within several of these anatomical interactions, and to explain using this expanded model how various brain lesions to areas involved in eyeblink conditioning may cause abnormal learning and memory.

## nSTART model of adaptively timed eyeblink conditioning

Neural pathways that support the conditioned eyeblink response involve various hierarchical and parallel circuits (Thompson, 1988; Woodruff-Pak & Steinmetz, 2000a, 2000b). The nSTART macrocircuit (Fig. 2) simulates key processes that exist within the wider network that supports the eyeblink response *in vivo* and highlights circuitry required for adaptively timed trace conditioning. Thalamus and sensory cortex are lumped into one sensory cortical representation for representational simplicity. However, the exposition of the model and its output pathways will require discussion of independent thalamocortical and corticocortical pathways. Different experimental manipulations affect brain regions like the thalamus, cortex, amygdala, and hippocampus in different ways. Our model computer simulations illustrate these differences. In addition, it is important to explain how these several individual responses of different brain regions contribute to a final common path the activity of which covaries with observed conditioned responses. Outputs from these brain regions meet directly or indirectly at the pontine nucleus, the final common bridge to the cerebellum which generates the CR (Freeman & Muckler, 2003; Kalmbach et al. 2009a, b; Siegel et al., 2012; Woodruff-Pak & Disterhoft, 2007). Simulations of how the model pontine nucleus responds to the aggregate effect of all the other brain regions are thus also provided. The internal dynamics of the cerebellum are not, however, simulated in the nSTART model; but see Fiala, Grossberg, and Bullock (1996) for a detailed cerebellar learning model that simulates how Ca^++^ can modulate mGluR dynamics to adaptively time responses across long ISIs.

## Normal and amnesic delay conditioning and trace conditioning

The ability to associatively learn what subset of earlier events predicts, or causes, later consequences, and what event combinations are not predictive, is a critical survival competence in normal adaptive behavior. In this section, data are highlighted that describe the differences between the normal and abnormal acquisition and retention of associative learning relative to the specific role of interactions among the processing areas in nSTART’s functional anatomy; notably, interactions between sensory cortex and thalamus, prefrontal cortex, amygdala, and hippocampus. See ‘*Methods* *,’* for an exposition of design principles and heuristic modeling concepts that go into the nSTART model; *‘* *Model description* *,’* for a non-technical exposition of the model processes and their interactions; *‘Results,’* for model simulations of data; *‘* *Discussion* *,’* for a general summary; and *‘Mathematical Equations and Parameters,’* for a complete summary of the model mechanisms.

Lesion data show that delay conditioning requires the cerebellum but does not need the hippocampus to acquire an adaptively timed conditioned response. Studies of hippocampal lesions in rats, rabbits, and humans reveal that, if a lesion occurs before delay conditioning (Daum, Schugens, Breitenstein, Topka, & Spieker, 1996; Ivkovich & Thompson, 1997; Schmaltz & Theios, 1972; Solomon & Moore, 1975; Weiskrantz & Warrington, 1979;), or any time after delay conditioning (Akase, Alkon, & Disterhoft, 1989; Orr & Berger, 1985; Port et al., 1986), the subject can still acquire or retain a CR. Depending on the performance criteria, sometimes the acquisition is reported as facilitated (Berger, 1984; Chen, 1995; Lee & Kim, 2004; Port, 1985; Shors, 1992).

Lee and Kim (2004) presented electromyography (EMG) data showing that amygdala lesions in rats decelerated delay conditioning if made prior to training, but not if made post-training, while hippocampal lesions accelerated delay conditioning if made prior to training. They found a time-limited role of the amygdala similar to the time-limited role of the hippocampus: The amygdala is more active during early acquisition than later. In addition, they found that the amygdala without the hippocampus is not sufficient for trace conditioning. During functional magnetic resonance imaging (fMRI) studies of human trace conditioning, Büchel et al. (1999) also found decreases in amygdala responses over time. They cited other fMRI studies that found robust hippocampal activity in trace conditioning, but not delay conditioning, to underscore their hypothesis that, while the amygdala may contribute to trace conditioning, the hippocampus is required. Chau and Galvez (2012) discussed the likelihood of the same time-limited involvement of the amygdala in trace eyeblink conditioning.

Holland and Gallagher (1999) reviewed literature describing the role of the amygdala as either modulatory or required, depending on specific connections with other brain systems, for normal “functions often characterized as attention, reinforcement and representation” (p. 66). Aggleton and Saunders (2000) described the amygdala in terms of four functional systems (accessory olfactory, main olfactory, autonomic, and frontotemporal). In the macaque monkey, ten interconnected cytotonic areas were defined within the amygdala, with 15 types of cortical inputs and 17 types of cortical projections, and 22 types of subcortical inputs from the amygdala and 15 types of subcortical projections to the amygdala (their Figs. 1.2–1.7, pp. 4–9). Given this complexity, the data are mixed about whether the amygdala is required for acquisition, or retention after consolidation, depending on the cause (cytotoxin, acid or electronic burning, cutting), target area, and degree of lesion, as well as the strength of the US, learning paradigm, and specific task (Blair, Sotres-Bayon, Moiya, & LeDoux, 2005; Cahill & McGaugh, 1990; Everitt, Cardinal, Hall, Parkinson, & Robbins, 2000; Kapp, Wilson, Pascoe, Supple, & Whalen, 1990; Killcross, Everitt, & Robbins, 1997; Lehmann, Treit, & Parent, 2000; Medina, Repa, Mauk, & LeDoux, 2002; Neufeld & Mintz, 2001; Oswald, Maddox, Tisdale, & Powell, 2010; Vazdarjanova & McGaugh, 1998). In fact, "…aversive eyeblink conditioning…survives lesions of either the central or basolateral parts of the amygdala" (Thompson et al. 1987). Additionally, such lesions have been found not to prevent Pavlovian appetitive conditioning or other types of appetitively-based learning (McGaugh, 2002, p.456).

These inconsistencies among the data may exist due to the contributions from multiple pathways that support emotion. For example, within the MOTIVATOR model extension of the CogEM model (see below), hypothalamic and related internal homeostatic and drive circuits may function without amygdala (Dranias et al., 2008). The nSTART model only incorporates an afferent cortical connection from the amygdala to represent incentive motivational learning signals. Within the cortex, however, the excitatory inputs from both the amygdala and hippocampus are modulated by the strength of thalamocortical signals.

A clear pattern emerges from comparing various data that disclose essential functions of the hippocampus, functions that are qualititatively simulated in nSTART. The hippocampus has been studied with regard to the acquisition of trace eyeblink conditioning, and the adaptive timing of conditioned responses (Berger, Laham, & Thompson, 1980; Mauk & Ruiz, 1992; Schmaltz & Theios, 1972; Sears & Steinmetz, 1990; Woodruff-Pak, 1993; Woodruff-Pak & Disterhoft, 2007). If a hippocampal lesion or other system disruption occurs before trace conditioning acquisition (Ivkovich & Thompson, 1997; Kaneko & Thompson, 1997; Weiss & Thompson, 1991b; Woodruff-Pak, 2001), or shortly thereafter (Kim et al., 1995; Moyer, Deyo, & Disterhoft, 1990; Takehara et al., 2003), the CR is not obtained or retained. Trace conditioning is impaired by pre-acquisition hippocampal lesions created during laboratory experimentation on animals (Anagnostaras, Maren, & Fanselow, 1999; Berry & Thompson, 1979; Garrud et al., 1984; James, Hardiman, & Yeo, 1987; Kim et al., 1995; Orr & Berger, 1985; Schmajuk, Lam, & Christiansen, 1994; Schmaltz & Theios, 1972; Solomon & Moore, 1975), and in humans with amnesia (Clark & Squire, 1998; Gabrieli et al., 1995; McGlinchey-Berroth, Carrillo, Gabrieli, Brawn, & Disterhoft, 1997), Alzheimer’s disease, or age-related deficits (Little, Lipsitt, & Rovee-Collier, 1984; Solomon et al., 1990; Weiss & Thompson, 1991a; Woodruff-Pak 2001).

The data show that, during trace conditioning, there is successful post-acquisition performance of the CR only if the hippocampal lesion occurs after a critical period of hippocampal support of memory consolidation within the neocortex (Kim et al., 1995; Takashima et al., 2009; Takehara et al., 2003). Data from *in vitro* cell preparations also support the time-limited role of the hippocampus in new learning that is simulated in nSTART: activity in hippocampal CA1 and CA3 pyramidal neurons peaked 24 h after conditioning was completed and decayed back to baseline within 14 days (Thompson, Moyer, & Disterhoft, 1996). The effect of early versus late hippocampal lesions is challenging to explain since no overt training occurs after conditioning during the period before hippocampal ablation.

After consolidation due to hippocampal involvement is accomplished, thalamocortical signals in conjunction with the cerebellum determine the timed execution of the CR during performance (Gabreil, Sparenborg, & Stolar, 1987; Sosina, 1992). Indeed, “…there are two memory circuitries for trace conditioning. One involves the hippocampus and the cerebellum and mediates recently acquired memory; the other involves the mPFC and the cerebellum and mediates remotely acquired memory” (Takehara et al., 2003, p. 9904; see also Berger, Weikart, Basset, & Orr, 1986; O'Reilly et al., 2010). nSTART qualitatively models these data as follows: after the consolidation of memory, when there is no need for hippocampus, nSTART models the cortical connections to the pontine nuclei that serve to elicit conditioned responses by way of the cerebellum (Siegel, Kalmback, Chitwood, & Mauk, 2012; Woodruff-Pak & Disterhoft, 2007).

Based on the extent and timing of hippocampal damage, learning impairments range from needing more training trials than normal in order to learn successfully, through persistent response-timing difficulties, to the inability to learn and form new memories. The nSTART model explains the need for the hippocampus during trace conditioning in terms of how the hippocampus supports strengthening of partially conditioned thalamocortical and cortiocortical connections during memory consolidation (see Fig. 2). The hippocampus has this ability because it includes circuits that can bridge the temporal gaps between CS and US during trace conditioning, unlike the amygdala, and can learn to adaptively time these temporal gaps in its responses, as originally simulated in the START model (Grossberg & Merrill, 1992, 1996; Grossberg & Schmajuk, 1989). The current nSTART model extends this analysis using mechanisms of endogenous hippocampal activation and BDNF modulation (see below) to explain the time-limited role of the hippocampus in terms of its support of the consolidation of new learning into long-term memories. This hypothesis is elaborated and contrasted with alternative models of memory consolidation below (*‘Multiple hippocampal functions: Space, time, novelty, consolidation, and episodic learning’)*.

## Conditioning and consciousness

Several studies of humans have described a link between consciousness and conditioning. Early work interpreted conscious awareness as another class of conditioned responses (Grant, 1973; Hilgard, Campbell, & Sears, 1937; Kimble, 1962; McAllister & McAllister, 1958). More recently, it was found that, while amnesic patients with hippocampal damage acquired delay conditioning at a normal rate, they failed to acquire trace conditioning (Clark & Squire, 1998). These experimenters postulated that normal humans acquire trace conditioning because they have intact declarative or episodic memory and, therefore, can demonstrate conscious knowledge of a temporal relationship between CS and US: “trace conditioning requires the acquisition and retention of conscious knowledge” (p. 79). They did not, however, discuss mechanisms underlying this ability, save mentioning that the neocortex probably represents temporal relationships between stimuli and “would require the hippocampus and related structures to work conjointly with the neocortex” (p.79).

Other studies have also demonstrated a link between consciousness and conditioning (Gabrieli et al., 1995; McGlinchey-Berroth, Brawn, & Disterhoft, 1999; McGlinchey-Berroth et al., 1997) and described an essential role for awareness in declarative learning, but no necessary role in non-declarative or procedural learning, as illustrated by experimental findings related to trace and delay conditioning, respectively (Manns, Clark, & Squire, 2000; Papka, Ivry, & Woodruff-Pak, 1997). For example, trace conditioning is facilitated by conscious awareness in normal control subjects while delay conditioning is not, whereas amnesics with bilateral hippocampal lesions perform at a success rate similar to unaware controls for both delay and trace conditioning (Clark, Manns, & Squire, 2001). Amnesics were found to be unaware of experimental contingencies, and poor performers on trace conditioning (Clark & Squire, 1998). Thus, the link between adaptive timing, attention, awareness, and consciousness has been experimentally established within the trace conditioning paradigm. The nSTART model traces the link between consciousness and conditioning to the role of hippocampus in supporting a sustained cognitive-emotional resonance that underlies motivated attention, consolidation of long-term memory, core consciousness, and "the feeling of what happens" (Damasio, 1999).

## Brain-derived neurotrophic factor (BDNF) in memory formation and consolidation

Memory consolidation, a process that supports an enduring memory of new learning, has been extensively studied: (McGaugh, 2000, 2002; Mehta, 2007; Nadel & Bohbot, 2001; Takehara, Kawahara, & Krino, 2003; Squire & Alverez, 1995; Takashima, 2009; Thompson, Moyer, & Disterhoft, 1996; Tyler, et al. 2002). These data show time-limited involvement of the limbic system, and long-term involvement of the neocortex. The question of what sort of process occurs during the period that actively strengthens memory, even when there is no explicit practice, has been linked to the action of neurotrophins (Zang, et al., 2007), especially BDNF, a complex class of proteins that have important effects on learning and memory (Heldt, Stanek, Chhatwal, & Ressler, 2007; Hu & Russek, 2008; Monteggia et al., 2004; Purves, 1988; Rattiner, Davis, & Ressler, 2005; Schuman, 1999; Thoenen, 1995; Tyler, Alonso, Bramham, & Pozzo-Miller, 2002). Postsynaptically, neurotrophins enhance responsiveness of target synapses (Kang & Schuman, 1995; Kohara, Kitamura, Morishima, & Tsumoto, 2001) and allow for quicker processing (Knipper et al., 1993; Lessman, 1998). Presynaptically, they act as retrograde messengers (Davis & Murphy, 1994; Ganguly, Koss, & Poo, 2000) coming from a target cell population back to excitatory source cells and increasing the flow of transmitter from the source cell population to generate a positive feedback loop between the source and the target cells (Schinder, Berninger, & Poo, 2000), as also occurs in some neural models of learning and memory search (e.g., Carpenter & Grossberg, 1990). BDNF has also been interpreted as an essential component of long-term potentiation (LTP) in normal cell processing (Chen, Kolbeck, Barde, Bonhoeffer, & Kossel, 1999; Korte et al., 1995; Phillips et al., 1990). The functional involvement of existing BDNF receptors is critical in early LTP (up to 1 h) during the acquisition phase of learning the CR, whereas continued activation of the slowly decaying late phase LTP signal (3+ h) requires new protein synthesis and gene expression. Rossato et al. (2009) have shown that hippocampal dopamine and the ventral tegmental area provide a temporally sensitive trigger for the expression of BDNF that is essential for long-term consolidation of memory related to reinforcement learning.

The BDNF response to a particular stimulus event may vary from microseconds (initial acquisition) to several days or weeks (long-term memory consolidation); thus, neurotrophins have a role whether the phase of learning is one of initial synaptic enhancement or long-term memory consolidation (Kang, Welcher, Shelton, & Schuman, 1997; Schuman, 1999; Singer, 1999). Furthermore, BDNF blockade shows that BDNF is essential for memory development at different phases of memory formation (Kang et al., 1997), and during all ages of an individual (Cabelli, Hohn, & Shatz, 1995; Tokuka, Saito, Yorifugi, Kishimoto, & Hisanaga, 2000). As nSTART qualitatively simulates, neurotrophins are thus required for both the initial acquisition of a memory and for its ongoing maintenance as memory consolidates.

BDNF is heavily expressed in the hippocampus as well as in the neocortex, where neurotrophins figure largely in activity-dependent development and plasticity, not only to build new bridges as needed, but also to inhibit and dismantle old synaptic bridges. A process of competition among axons during the development of nerve connections (Bonhoffer, 1996; Tucker, Meyer, & Barde, 2001; van Ooyen & Willshaw, 1999; see review in Tyler et al., 2002), exists both in young and mature animals (Phillips, Hains, Laramee, Rosenthal, & Winslow, 1990). BDNF also maintains cortical circuitry for long-term memory that may be shaped by various BDNF-independent factors during and after consolidation (Gorski, Zeiler, Tamowski, & Jones, 2003).

The nSTART model hypothesizes how BDNF may amplify and temporally extend activity-based signals within the hippocampus and the neocortex that facilitate endogenous strengthening of memory without further explicit learning. In particular, memory consolidation may be mechanistically achieved by means of a sustained cascade of BDNF expression beginning in the hippocampus and spreading to the cortex (Buzsáki & Chrobak, 2005; Cousens & Otto, 1998; Hobson & Pace-Schott, 2002; Monteggia, et al., 2004; Nádasdy, Hirase, Czurkó, Csicsvari, & Buzsáki, 1999; Smythe, Colom, & Bland, 1992; Staubli & Lynch, 1987; Vertes, Hoover, & Di Prisco, 2004), which is modeled in nSTART by the maintained activity level of hippocampal and cortical BDNF after conditioning trials end (see Fig. 2).

Hippocampal bursting activity is not the only bursting activity that drives consolidation. Long-term activity-dependent consolidation of new learning is also supported by the synchronization of thalamocortical interactions in response to thalamic or cortical inputs (Llinas, Ribary, Joliot, & Wang, 1994; Steriade, 1999). Thalamic bursting neurons may lead to synaptic modifications in cortex, and cortex can in turn influence thalamic oscillations (Sherman & Guillery, 2003; Steriade, 1999). Thalamocortical resonance has been described as a basis for temporal binding and consciousness in increasingly specific models over the years. These models simulate how specific and nonspecific thalamic nuclei interact with the reticular nucleus and multiple stages of laminar cortical circuitry (Buzsáki, Llinás, Singer, Berthoz, & Christen, 1994; Engel, Fries, & Singer, 2001; Grossberg, 1980, 2003, 2007; Grossberg & Versace, 2008; Pollen, 1999; Yazdanbakhsh & Grossberg, 2004). nSTART qualitatively explains consolidation without including bursting phenomena, although oscillatory dynamics of this kind arise naturally in finer spiking versions of rate-based models such as nSTART (Grossberg & Versace, 2008; Palma, Grossberg, & Versace, 2012a, 2012b).

The nSTART model focuses on amygdala and hippocampal interactions with thalamus and neocortex during conditioning (Fig. 2). The model proposes that the hippocampus supports thalamo-cortical and cortico-cortical category learning that becomes well established during memory consolidation through its endogenous (bursting) activity (Siapas, Lubenov, & Wilson, 2005; Sosina, 1992) that is supported by neurotrophin mediators (Destexhe, Contreras & Steriade, 1998). nSTART proposes that thalamo-cortical sustained activity is maintained through the combination of two mechanisms: the level of cortical BDNF activity, and the strength of the learned thalamo-cortical adaptive weights, or long-term memory (LTM) traces that were strengthened by the memory consolidation process. This proposal is consistent with trace conditioning data showing that, after consolidation, when the hippocampus is no longer required for performance of CRs, the medial prefrontal cortex takes on a critical role for performance of the CR in reaction to the associated thalamic sensory input, Here, the etiology of retrograde amnesia is understood as a failure to retain memory, rather than as a failure of adaptive timing (Takehara et al., 2003).

## Methods

### From CogEM to nSTART

The nSTART model synthesizes and extends key principles, mechanisms, and properties of three previously published brain models of conditioning and behavior. These three models describe aspects of:

1. How the brain learns to categorize objects and events in the world (Carpenter & Grossberg, 1987, 1991, 1993; Grossberg, 1976a, 1976b, 1980, 1982, 1984, 1987, 1999, 2013; Raizada & Grossberg, 2003); this is described within Adaptive Resonance Theory, or ART;
2. How the brain learns the emotional meanings of such events through cognitive-emotional interactions, notably rewarding and punishing experiences, and how the brain determines which events are motivationally predictive, as during attentional blocking and unblocking (Dranias, Grossberg, & Bullock, 2008; Grossberg, 1971, 1972a, 1972b, 1980, 1982, 1984, 2000b; Grossberg, Bullock, & Dranias, 2008; Grossberg & Gutowski, 1987; Grossberg & Levine, 1987; Grossberg & Schmajuk, 1987); this is described within the Cognitive-Emotional-Motor, or CogEM, model; and
3. How the brain learns to adaptively time the attention that is paid to motivationally important events, and when to respond to these events, in a context-appropriate manner (Fiala, Grossberg, & Bullock, 1996; Grossberg & Merrill, 1992, 1996; Grossberg & Paine, 2000; Grossberg & Schmajuk, 1989); this is described within the START model.

All three component models have been mathematically and computationally characterized elsewhere in order to explain behavioral and brain data about normal and abnormal behaviors. The principles and mechanisms that these models employ have thus been independently validated through their ability to explain a wide range of data. nSTART builds on this foundation to explain data about conditioning and memory consolidation, as it is affected by early and late amygdala, hippocampal, and cortical lesions, as well as BDNF expression in the hippocampus and cortex. The exposition in this section heuristically states the main modeling concepts and mechanisms before building upon them to mathematically realize the current model advances and synthesis.

The simulated data properties emerge from interactions of several brain regions for which processes evolve on multiple time scales, interacting in multiple nonlinear feedback loops. In order to simulate these data, the model incorporates only those network interactions that are rate-limiting in generating the targeted data. More detailed models of the relevant brain regions, that are consistent with the model interactions simulated herein, are described below, and provide a guide to future studies aimed at incorporating a broader range of functional competences.

### Adaptive resonance theory

**Fig. 3:**
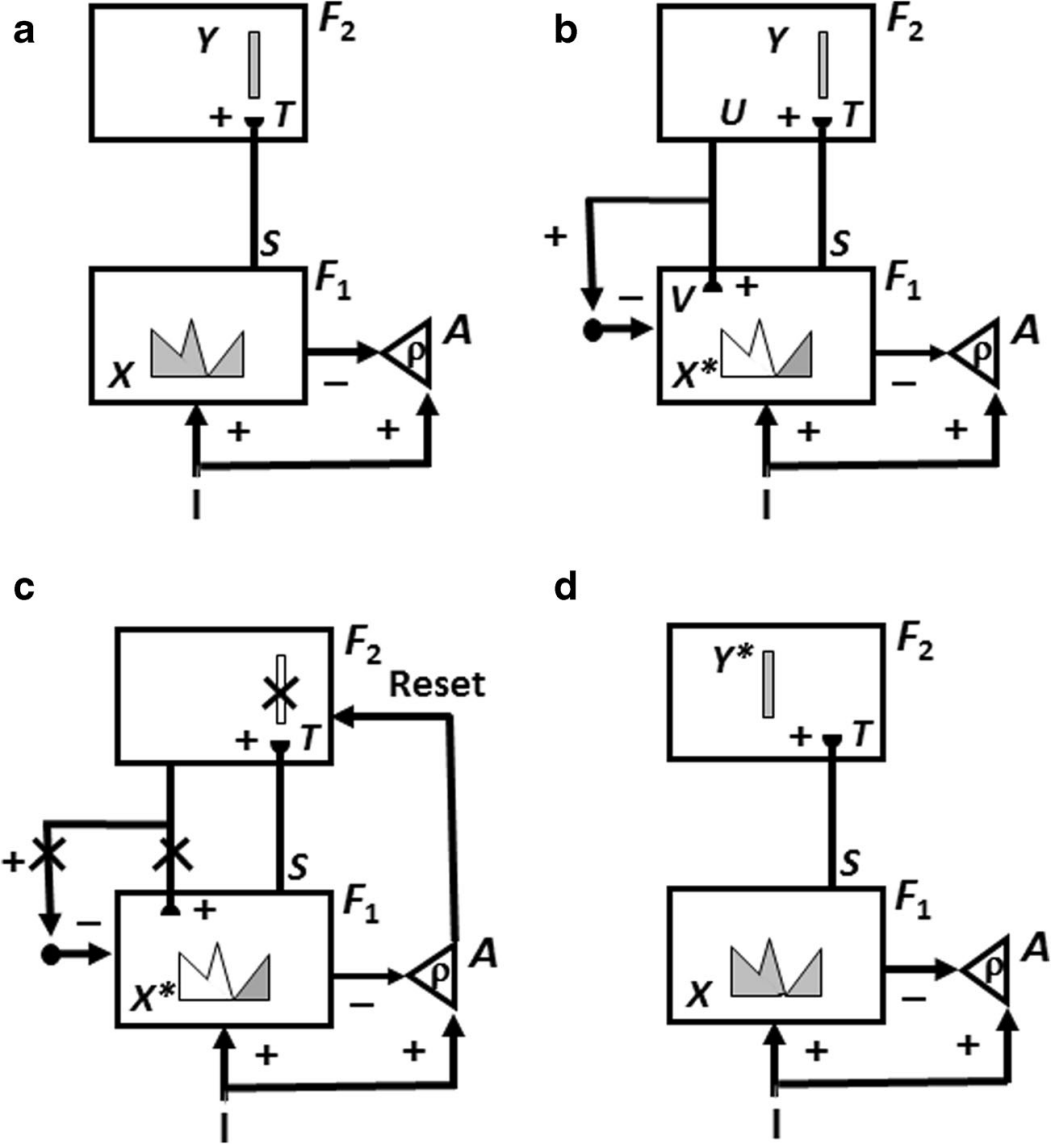
How ART searches for and learns a new recognition category using cycles of match-induced resonance and mismatch-induced reset. Active cells are shaded gray; inhibited cells are not shaded. (**a**) Input pattern *I* is instated across feature detectors at level *F*_*1*_ as an activity pattern *X*, at the same time that it generates excitatory signals to the orienting system *A* with a gain *ρ* that is called the *vigilance* parameter. Activity pattern *X* generates inhibitory signals to the orienting system *A* as it generates a bottom-up input pattern *S* to the category level *F*_*2*_. A dynamic balance within *A* between excitatory inputs from *I* and inhibitory inputs from *S* keeps *A* quiet. The bottom-up signals in *S* are multiplied by learned adaptive weights to form the input pattern *T* to *F*_*2*_. The inputs *T* are contrast-enhanced and normalized within *F*_*2*_ by recurrent lateral inhibitory signals that obey the membrane equations of neurophysiology, otherwise called shunting interactions. This competition leads to selection and activation of a small number of cells within *F*_*2*_ that receive the largest inputs. In this figure, a winner-take-all category is chosen, represented by a single cell (population). The chosen cells represent the category Y that codes for the feature pattern at *F*_*1*_. (**b**) The category activity *Y* generates top-down signals *U* that are multiplied by adaptive weights to form a prototype, or critical feature pattern, *V* that encodes the expectation that the active *F*_*2*_ category has learned for what feature pattern to expect at *F*_*1*_. This top-down expectation input *V* is added at *F*_*1*_ cells. If *V* mismatches *I* at *F*_*1*_, then a new STM activity pattern X* (the gray pattern), is selected at cells where the patterns match well enough. In other words, X* is active at *I* features that are confirmed by *V*. Mismatched features (white area) are inhibited. When *X* changes to X*, total inhibition decreases from *F*_*1*_ to *A*. (**c**) If inhibition decreases sufficiently, *A r*eleases a nonspecific arousal burst to *F*_*2*_; that is, “novel events are arousing”. Within the orienting system *A*, a vigilance parameter *ρ* determines how bad a match will be tolerated before a burst of nonspecific arousal is triggered. This arousal burst triggers a memory search for a better-matching category, as follows: Arousal resets *F*_*2*_ by inhibiting *Y.* (**d**) After *Y* is inhibited, *X* is reinstated and *Y* stays inhibited as *X* activates a different category, that is represented by a different activity winner-take-all category Y*, at *F*_*2.*_. Search continues until a better matching, or novel, category is selected. When search ends, an attentive resonance triggers learning of the attended data in adaptive weights within both the bottom-up and top-down pathways. As learning stabilizes, inputs *I* can activate their globally best-matching categories directly through the adaptive filter, without activating the orienting system [Adapted with permission from Carpenter and Grossberg (1987)]

The first model upon which nSTART builds is called Adaptive Resonance Theory, or ART. ART is reviewed because a key process in nSTART is a form of category learning, and also because nSTART simulates a cognitive-emotional resonance that is essential for explaining its targeted data. ART proposes how the brain can rapidly learn to attend, recognize, and predict new objects and events without catastrophically forgetting memories of previously learned objects and events. This is accomplished through an attentive matching process between the feature patterns that are created by stimulus-driven bottom-up adaptive filters, and learned top-down expectations (Fig. 3). The top-down expectations, acting by themselves, can also prime the brain to anticipate future bottom-up feature patterns with which they will be matched.

In nSTART, it is assumed that each CS and US is familiar and has already undergone category learning before the current simulations begin. The CS and US inputs to sensory cortex in the nSTART macrocircuit are assumed to be processed as learned object categories (Fig. 2). nSTART models a second stage of category learning from an object category in sensory cortex to an object-value category in orbitofrontal cortex. In general, each object category can become associated with more than one object-value category, so the same sensory cue can learn to generate different conditioned responses in response to learning with different reinforcers. It does this by learning to generate different responses when different value categories are active. These adaptive connections are thus, in general, one-to-many. Conceptually, the two stages of learning, at the object category stage and the object-value category stage, can be interpreted as a coordinated category learning process through which the orbitofrontal cortex categorizes objects and their motivational significance (Barbas, 1995, 2007; Rolls, 1998, 2000). The current model simulates such conditioning with only a single type of reinforcer. Strengthening the connection from object category to object-value category represents a simplified form of this category learning process in the current model simulations. One-to-many learning from an object category to multiple object-value categories is simulated in Chang, Grossberg, and Cao (2014).

As in other ART models, a top-down expectation pathway also exists from the orbitofrontal cortex to the sensory cortex. It provides top-down attentive modulation of sensory cortical activity, and is part of the cortico-cortico-amygdalar-hippocampal resonance that develops in the model during learning. This *cognitive-emotional* resonance, which plays a key role in the current model and its simulations, as well as its precursors in the START and iSTART models, is the main reason that nSTART is considered to be part of the family of ART models. Indeed, Grossberg (2016) summarizes an emerging classification of brain resonances that support conscious seeing, hearing, feeling, and knowing that includes this cognitive-emotional resonance.

nSTART explains how this cognitive-emotional resonance is sustained through time by adaptively-timed hippocampal feedback signals (Fig. 2). This hippocampal feedback plays a critical role in the model’s explanation of data about memory consolidation, and its ability to explain how the brain bridges the temporal gap between stimuli that occur in experimental paradigms like trace conditioning. Consolidation is complete within nSTART when the hippocampus is no longer needed to further strengthen the category memory that is activated by the CS. Finally, the role of the hippocampus in sustaining the cognitive-emotional resonances helps to explain the experimentally reported link between conditioning and consciousness (Clark & Squire, 1998).

In a complete ART model, when a sufficiently good match occurs between a bottom-up input pattern and an active top-down expectation, the system locks into a resonant state that focuses attention on the matched features and drives learning to incorporate them into the learned category; hence the term *adaptive* resonance. ART also predicts that *all conscious states are resonant states,* and the Grossberg (2016) classification of resonances contributes to clarifying their diverse functions throughout the brain**.** Such an adaptive resonance is one of the key mechanisms whereby ART ensures that memories are dynamically buffered against catastrophic forgetting. As noted above, a simplified form of this attentive matching process is included in nSTART in order to explain the cognitive-emotional resonances that support memory consolidation and the link between conditioning and consciousness.

In addition to the attentive resonant state itself, a hypothesis testing, or memory search, process in response to unexpected events helps to discover predictive recognition categories with which to learn about novel environments, and to switch attention to new inputs within a known environment. This hypothesis testing process is not simulated herein because the object categories that are activated in response to the CS and US stimuli are assumed to already have been learned, and unexpected events are minimized in the kinds of highly controlled delay and trace conditioning experiments that are the focus of the current study.

For the same reason, another mechanism that is important during hypothesis testing is not included in nSTART. The degree of match between bottom-up and top-down signal patterns that is required for resonance, sustained attention, and learning to occur is set by a *vigilance* parameter (Carpenter & Grossberg, 1987) (see ρ in Fig. 3a). Vigilance may be increased by predictive errors, and controls whether a particular learned category will represent concrete information, such as a particular view of a particular face, or abstract information, such as the fact that everyone has a face. Low vigilance allows the learning of general and abstract recognition categories, whereas high vigilance forces the learning of specific and concrete categories. The current simulations do not need to vary the degree of abstractness of the categories to be learned, so vigilance control has been omitted for simplicity.

A big enough mismatch designates that the selected category does not represent the input data well enough, and drives a memory search, or hypothesis testing, for a category that can better represent the input data. In a more complete nSTART model, hypothesis testing would enable the learning and stable memory of large numbers of thalamo-cortical and cortico-cortical recognition categories. Such a hypothesis testing process includes a novelty-sensitive orienting system *A*, which is predicted to include both the nonspecific thalamus and the hippocampus (Fig. 3c; Carpenter & Grossberg, 1987, 1993; Grossberg, 2013; Grossberg & Versace, 2008). In nSTART, the model hippocampus does include the crucial process of adaptively timed learning that can bridge temporal gaps of hundreds of milliseconds to support trace conditioning and memory consolidation. In a more general nSTART model that is capable of self-stabilizing its learned memories, the hippocampus would also be involved in the memory search process.

In an ART model that includes memory search, when a mismatch occurs, the orienting system is activated and generates nonspecific arousal signals to the attentional system that rapidly reset the active recognition categories that have been reading out the poorly matching top-down expectations (Fig. 3c). The cause of the mismatch is hereby removed, thereby freeing the bottom-up filter to activate a different recognition category (Fig. 3d). This cycle of mismatch, arousal, and reset can repeat, thereby initiating a memory search, or hypothesis testing cycle, for a better-matching category. If no adequate match with a recognition category exists, say because the bottom-up input represents an unfamiliar experience, then the search process automatically activates an as yet uncommitted population of cells, with which to learn a new recognition category to represent the novel information.

All the learning and search processes that ART predicted have received support from behavioral, ERP, anatomical, neurophysiological, and/or neuropharmacological data, which are reviewed in the ART articles listed above; see, in particular, Grossberg (2013). Indeed, the role of the hippocampus in novelty detection has been known for many years (Deadwyler, West, & Lynch, 1979; Deadwyler et al., 1981; Vinogradova, 1975). In particular, the hippocampal CA1 and CA3 regions have been shown to be involved in a process of comparison between a prior conditioned stimulus and a current stimulus by rats in a non-spatial auditory task, the continuous non-matching-to-sample task (Sakurai, 1990). During performance of the task, single unit activity was recorded from several areas: CA1 and CA3, dentate gyrus (DG), entorhinal cortex, subicular complex, motor cortex (MC), prefrontal cortex, and dorsomedial thalamus. Go and No-Go responses indicated, respectively, whether the current tone was perceived as the same as (match) or different from (non-match) the preceding tone. Since about half of the units from the MC, CA1, CA3, and DG had increments of activity immediately prior to a Go response, these regions were implicated in motor or decisional aspects of making a match response. On non-match trials, units were also found in CA1 and CA3 with activity correlated to a correct No-Go response. Corroborating the function of the hippocampus in recognition memory, but not in storing the memories themselves, Otto and Eichenbaum (1992) reported that CA1 cells compare cortical representations of current perceptual processes to previous representations stored in parahippocampal and neocortical structures to detect mismatch in an odor-guided task. They noted that “the hippocampus maintains neither active nor passive memory representations” (p. 332).

Grossberg and Versace (2008) have proposed how the nonspecific thalamus can also be activated by novel events and trigger hypothesis testing. In their Synchronous Matching ART (SMART) model, a predictive error can lead to a mismatch within the nucleus basalis of Meynert, which releases acetylcholine broadly in the neocortex, leading to an increase in vigilance and a memory search for a better matching category. Palma, Grossberg, and Versace (2012a) and Palma, Versace, and Grossberg (2012b) further model how acetylcholine-modulated processes work, and explain a wide range of data using their modeling synthesis.

### CogEM and MOTIVATOR models

Recognition categories can be activated when objects are experienced, but do not reflect the emotional or motivational value of these objects. Such a recognition category can, however, be associated through reinforcement learning with one or more drive representations, which are brain sites that represent internal drive states and emotions. Activation of a drive representation by a recognition category can trigger emotional reactions and incentive motivational feedback to recognition categories, thereby amplifying valued recognition categories with motivated attention as part of a cognitive-emotional resonance between the inferotemporal cortex, amygdala, and orbitofrontal cortex. When a recognition category is chosen in this way, it can trigger choice and release of actions that realize valued goals in a context-sensitive way.

Such internal drive states and motivational decisions are incorporated into nSTART using mechanisms from the second model, called the Cognitive-Emotional-Motor, or CogEM, model. CogEM simulates the learning of cognitive-emotional associations, notably associations that link external objects and events in the world to internal feelings and emotions that give these objects and events value (Fig. 3a and b). These emotions also activate the motivational pathways that energize actions aimed at acquiring or manipulating objects or events to satisfy them.

The CogEM model clarifies interactions between two types of homologous circuits: one circuit includes interactions between the thalamus, sensory cortex, and amygdala; the other circuit includes interactions between the sensory cortex, orbitofrontal cortex, and amygdala. The nSTART model (Fig. 2) simulates cortico-cortico-amygdalar interactions. At the present level of simplification, the same activation and learning dynamics could also simulate interactions between thalamus, sensory cortices, and the amygdala. In particular, the CogEM model proposes how emotional centers of the brain, such as the amygdala, interact with sensory and prefrontal cortices – notably the orbitofrontal cortex – to generate affective states, attend to motivationally salient sensory events, and elicit motivated behaviors. Neurophysiological data provide increasing support for the predicted role of interactions between the amygdala and orbitofrontal cortex in focusing motivated attention on cell populations that can select learned responses which have previously succeeded in acquiring valued goal objects (Baxter et al., 2000; Rolls, 1998, 2000; Schoenbaum, Setlow, Saddoris, & Gallagher, 2003).

In ART, resonant states can develop within sensory and cognitive feedback loops. Resonance can also occur within CogEM circuits between sensory and cognitive representations of the external world and emotional representations of what is valued by the individual. Activating the (sensory cortex)-(amygdala)-(prefrontal cortex) feedback loop between cognitive and emotional centers is predicted to generate a cognitive-emotional resonance that can support conscious awareness of events happening in the world and how we feel about them. This resonance tends to focus attention selectively upon objects and events that promise to satisfy emotional needs. Such a resonance, when it is temporally extended to also include the hippocampus, as described below, helps to explain how trace conditioning occurs, as well as the link between conditioning and consciousness that has been experimentally reported.

**Fig. 4:**
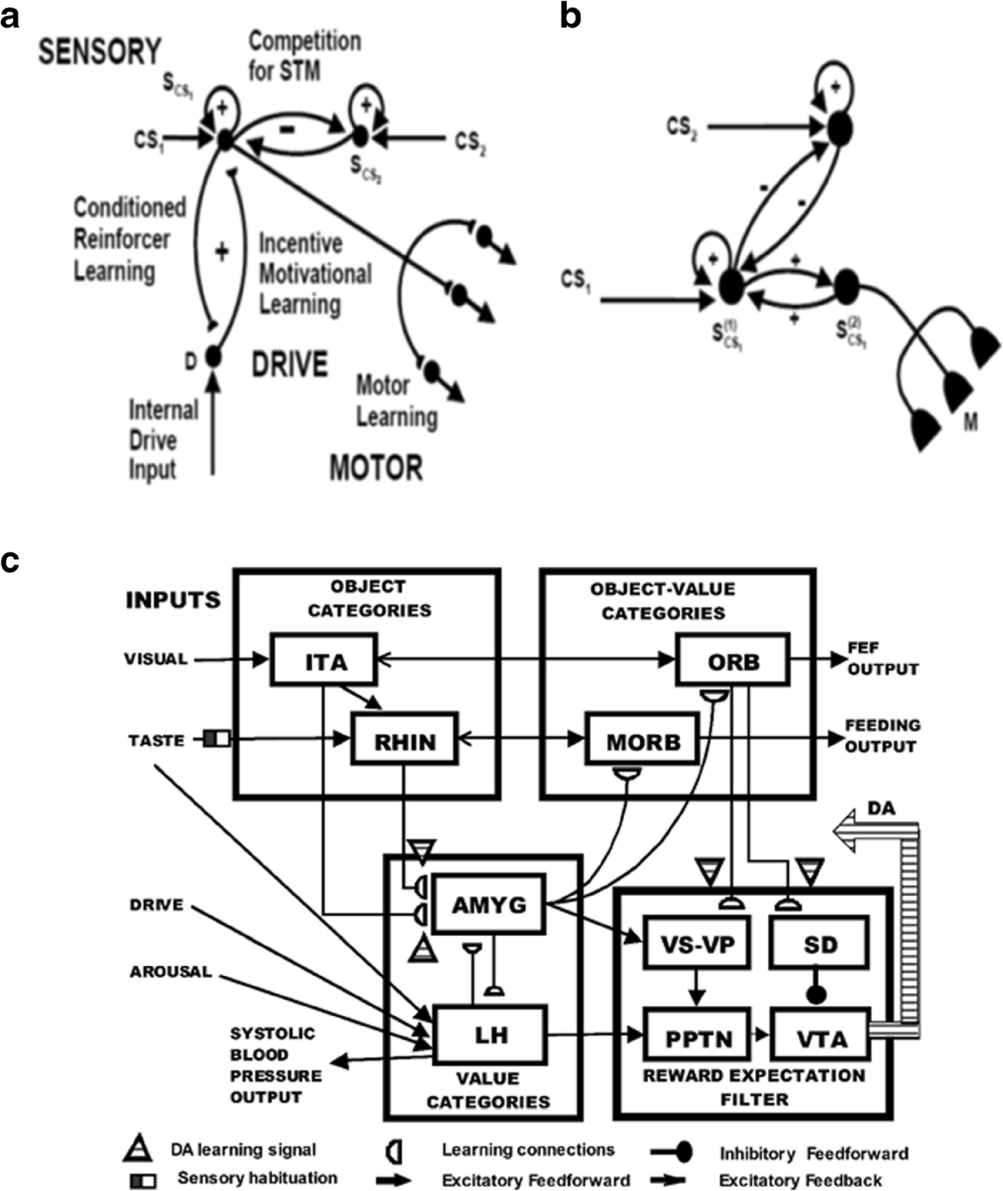
(**a**) The simplest Cognitive-Emotional-Motor (CogEM) model: Three types of interacting representations (sensory, S; drive, D; and motor, M) that control three types of learning (conditioned reinforcer, incentive motivational, and motor) help to explain many reinforcement learning data. (**b**) In order to work well, a sensory representation S must have (at least) two successive stages, S^(1)^ and S^(2)^, so that sensory events cannot release actions that are motivationally inappropriate. The two successive stages of a sensory representation S are interpreted to be in the appropriate sensory cortex (corresponds to S^(1)^) and the prefrontal cortex, notably the orbitofrontal cortex (corresponds to S^(2)^). The prefrontal stage requires motivational support from a drive representation D such as amygdala, to be fully effective, in the form of feedback from the incentive motivational learning pathway. Amydgala inputs to prefrontal cortex cause feedback from prefrontal cortex to sensory cortex that selectively amplifies and focuses attention upon motivationally relevant sensory events, and thereby “attentionally blocks” irrelevant cues. [Reprinted with permission from Grossberg and Seidman (2006).] (**c**) The amygdala and basal ganglia work together, embodying complementary functions, to provide motivational support, focus attention, and release contextually appropriate actions to achieve valued goals. For example, the basal ganglia substantia nigra pars compacta (SNc) releases Now Print learning signals in response to unexpected rewards or punishments, whereas the amygdala generates incentive motivational signals that support the attainment of expected valued goal objects. The MOTIVATOR model circuit diagram shows cognitive-emotional interactions between higher-order sensory cortices and an evaluative neuraxis composed of the hypothalamus, amygdala, basal ganglia, and orbitofrontal cortex [Reprinted with permission from Dranias et al. (2008)]

Figure 4a and b summarize the CogEM hypothesis that (at least) three types of internal representation interact during classical conditioning and other reinforcement learning paradigms: sensory cortical representations S, drive representations D, and motor representations M. These representations, and the learning that they support, are incorporated into the nSTART circuit (Fig. 2).

*Sensory representations* S temporarily store internal representations of sensory events in short-term and working memory. *Drive representations* D are sites where reinforcing and homeostatic, or drive, cues converge to activate emotional responses. *Motor representations* M control the read-out of actions. In particular, the S representations are thalamo-cortical or cortico-cortical representations of external events, including the object recognition categories that are learned by inferotemporal and prefrontal cortical interactions (Desimone, 1991, 1998; Gochin, Miller, Gross, & Gerstein, 1991; Harries & Perrett, 1991; Mishkin, Ungerleider, & Macko, 1983; Ungerleider & Mishkin, 1982), and that are modeled by ART. Sensory representations temporarily store internal representations of sensory events, such as conditioned stimuli (CS) and unconditioned stimuli (US), in short-term memory via recurrent on-center off-surround networks that tend to conserve their total activity while they contrast-normalize, contrast-enhance, and store their input patterns in short-term memory (Fig. 4a and b).

**Fig. 5:**
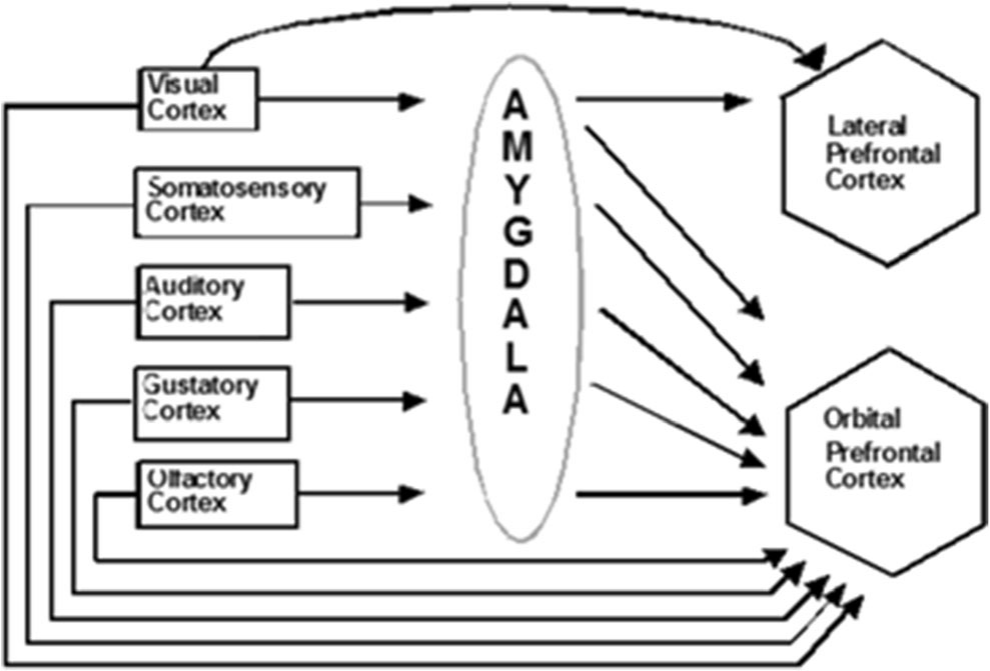
Orbital prefrontal cortex receives projections from the sensory cortices (visual, somatosensory, auditory, gustatory, and olfactory) and from the amygdala, which also receives inputs from the same sensory cortices. These anatomical stages correspond to the model CogEM stages in Fig. 4 [Reprinted with permission from Barbas (1995)]

The D representations include hypothalamic and amygdala circuits (Figs. 2 and 5) at which reinforcing and homeostatic, or drive, cues converge to generate emotional reactions and motivational decisions (Aggleton, 1993; Bower, 1981; Davis, 1994; Gloor et al., 1982; Halgren, Walter, Cherlow, & Crandall, 1978; LeDoux, 1993). The M representations include cortical and cerebellar circuits that control discrete adaptive responses (Evarts, 1973; Ito, 1984; Kalaska, Cohen, Hyde, & Prud’homme, 1989; Thompson, 1988). More complete models of the internal structure of these several types of representations have been presented elsewhere (e.g., Brown, Bullock, & Grossberg, 2004; Bullock, Cisek, & Grossberg, 1998; Carpenter & Grossberg, 1991; Contreras-Vidal, Grossberg, & Bullock, 1997; Dranias, Grossberg, & Bullock, 2008; Fiala, Grossberg, & Bullock, 1996; Gnadt & Grossberg, 2008; Grossberg, 1987; Grossberg, Bullock & Dranias, 2008; Grossberg & Merrill, 1996; Grossberg & Schmajuk, 1987; Raizada & Grossberg, 2003), and can be incorporated into future elaborations of nSTART without undermining any of the current model's conclusions.

nSTART does not incorporate the basal ganglia to simulate its targeted data, even though the basal ganglia and amygdala work together to provide motivational support, focus attention, and release contextually appropriate actions to achieve valued goals (Flores & Diserhoft, 2009). The MOTIVATOR model (Dranias et al., 2008; Grossberg et al., 2008) begins to explain how this interaction happens (Fig. 4c), notably how the amygdala and basal ganglia may play complementary roles during cognitive-emotional learning and motivated goal-oriented behaviors (Grossberg, 2000a). MOTIVATOR describes cognitive-emotional interactions between higher-order sensory cortices and an evaluative neuraxis composed of the hypothalamus, amygdala, basal ganglia, and orbitofrontal cortex. Given a conditioned stimulus (CS), the model amygdala and lateral hypothalamus interact to calculate the expected current value of the subjective outcome that the CS predicts, constrained by the current state of deprivation or satiation. As in the CogEM model, the amygdala relays the expected value information to orbitofrontal cells that receive inputs from anterior inferotemporal cells, and medial orbitofrontal cells that receive inputs from rhinal cortex. The activations of these orbitofrontal cells code the subjective values of objects. These values guide behavioral choices.

The model basal ganglia detect errors in CS-specific predictions of the value and timing of rewards. Excitatory inputs from the pedunculopontine nucleus interact with timed inhibitory inputs from model striosomes in the ventral striatum to regulate dopamine burst and dip responses from cells in the substantia nigra pars compacta and ventral tegmental area. Learning in cortical and striatal regions is strongly modulated by dopamine. The MOTIVATOR model is used to address tasks that examine food-specific satiety, Pavlovian conditioning, reinforcer devaluation, and simultaneous visual discrimination. Model simulations successfully reproduce discharge dynamics of known cell types, including signals that predict saccadic reaction times and CS-dependent changes in systolic blood pressure. In the nSTART model, these basal ganglia interactions are not needed to simulate the targeted data, hence will not be further discussed.

Even without basal ganglia dynamics, the CogEM model has successfully learned to control motivated behaviors in mobile robots (e.g., Baloch & Waxman, 1991; Chang & Gaudiano, 1998; Gaudiano & Chang, 1997; Gaudiano, Zalama, Chang, & Lopez-Coronado, 1996).

Three types of learning take place among the CogEM sensory, drive, and motor representations (Fig. 4a). *Conditioned reinforcer learning* enables sensory events to activate emotional reactions at drive representations. *Incentive motivational learning* enables emotions to generate a motivational set that biases the system to process cognitive information consistent with that emotion. *Motor learning* allows sensory and cognitive representations to generate actions. nSTART simulates both conditioned reinforcer learning, from thalamus to amygdala, or from sensory cortex to amygdala, as well as incentive motivational learning, from amygdala to sensory cortex, or from amygdala, to orbitofrontal cortex (Fig. 2). Instead of explicitly modeling motor learning circuits in the cerebellum, nSTART uses CR cortical and amygdala inputs to the pontine nucleus as indicators of the timing and strength of conditioned motor outputs (Freeman & Muckler, 2003; Kalmbach et al., 2009; Siegel et al., 2012; Woodruff-Pak & Disterhoft, 2007).

During classical conditioning, a CS activates its sensory representation S before the drive representation D is activated by an unconditioned simulus (US), or other previously conditioned reinforcer CSs. If it is appropriately timed, such pairing causes learning at the adaptive weights within the S → D pathway. The ability of the CS to subsequently activate D via this learned pathway is one of its key properties as a conditioned reinforcer. As these S → D associations are being formed, incentive motivational learning within the D → S incentive motivational pathway also occurs, due to the same pairing of CS and US. Incentive motivational learning enables an activated drive representation D to prime, or modulate, the sensory representations S of all cues, including the CSs, that have consistently been correlated with it. That is how activating D generates a “motivational set”: it primes all of the sensory and cognitive representations that have been associated with that drive in the past. These incentive motivational signals are a type of motivationally-biased attention. The S → M motor, or habit, learning enables the sensorimotor maps, vectors, and gains that are involved in sensory-motor control to be adaptively calibrated, thereby enabling a CS to read-out correctly calibrated movements as a CR.

Taken together, these processes control aspects of the learning and recognition of sensory and cognitive memories, which are often classified as part of the declarative memory system (Mishkin, 1982, 1993; Squire & Cohen, 1984); and the performance of learned motor skills, which are often classified as part of the procedural memory system (Gilbert & Thatch, 1977; Ito, 1984; Thompson, 1988).

Once both conditioned reinforcer and incentive motivational learning have taken place, a CS can activate a (sensory cortex)-(amygdala)-(orbitofrontal cortex)-(sensory cortex) feedback circuit (Figs. 2 and 4c). This circuit supports a cognitive-emotional resonance that leads to core consciousness and “the feeling of what happens” (Damasio, 1999), while it enables the brain to rapidly focus motivated attention on motivationally salient objects and events. This is the first behavioral competence that was mentioned above in the *Overview and scope*section. This feedback circuit could also, however, without further processing, immediately activate motor responses, thereby leading to premature responding in many situations.

We show below that this amygdala-based process is effective during delay conditioning, where the CS and US overlap in time, but not during trace conditioning, where the CS terminates before the US begins, at least not without the benefit of the adaptively timed learning mechanisms that are described in the next section. Thus, although the CogEM model can realize the first behavioral competence that is summarized above, it cannot realize the second and third competences, which involve bridging temporal gaps between CS, US, and conditioned responses (as discussed above). Mechanisms that realize the second and third behavioral competences enable the brain to learn during trace conditioning.

It is also important to acknowledge that, as reviewed above, the amygdala may have a time-limited role during aversive conditioning (Lee & Kim, 2004). As the association of eyeblink CS-US becomes more consolidated through the strengthening of direct thalamo-cortical and cortico-cortical learned associations, the role of the amygdala may become less critical.

### Spectral Timing model and hippocampal time cells

The third model, called the Spectral Timing model, clarifies how the brain learns adaptively timed responses in order to acquire rewards and other goal objects that are delayed in time, as occurs during trace conditioning. Spectral timing enables the model to bridge an ISI, or temporal gap, of hundreds of milliseconds, or even seconds, between the CS offset and US onset. This learning mechanism has been called *spectral timing* because a “spectrum” of cells respond at different, but overlapping, times and can together generate a population response for which adaptively timed cell responses become maximal at, or near, the time when the US is expected (Grossberg & Merrill, 1992, 1996; Grossberg & Schmajuk, 1989), as has been shown in neurophysiological experiments about adaptively timed conditioning in the hippocampus (Berger & Thompson, 1978; Nowak & Berger, 1992; see also Tieu et al., 1999).

Each cell in such a spectrum reaches its maximum activity at different times. If the cell responds later, then its activity duration is broader in time, a property that is called a Weber law, or scalar timing, property (Gibbon, 1977). Recent neurophysiological data about “time cells” in the hippocampus have supported the Spectral Timing model prediction of a spectrum of cells with different peak activity times that obey a Weber law. Indeed, such a Weber law property was salient in the data of MacDonald et al. (2011), who wrote: “…the mean peak firing rate for each time cell occurred at sequential moments, and the overlap among firing periods from even these small ensembles of time cells bridges the entire delay. Notably, the spread of the firing period for each neuron increased with the peak firing time…” (p. 3). MacDonald et al. (2011) have hereby provided direct neurophysiological support for the prediction of spectral timing model cells (“small ensembles of time cells”) that obey the Weber law property (“spread of the firing period…increased with the peak firing time”).

To generate the adaptively timed population response, each cell's activity is multiplied, or gated, by an adaptive weight before the memory-gated activity adds to the population response. During conditioning, each weight is amplified or suppressed to the extent to which its activity does, or does not, overlap times at which the US occurs; that is, times around the ISI between CS and US. Learning has the effect of amplifying signals from cells for which timing matches the ISI, at least partially. Most cell activity intervals do not match the ISI perfectly. However, after such learning, the sum of the gated signals from all the cells – that is, its population response – is well-timed to the ISI, and typically peaks at or near the expected time of US onset. This sort of adaptive timing endows the nSTART model with the ability to learn associations between events that are separated in time, notably between a CS and US during trace conditioning.

Evidence for adaptive timing has been found during many different types of reinforcement learning. For example, classical conditioning is optimal at a range of inter-stimulus intervals between the CS and US that are characteristic of the task, species, and age, and is typically attenuated at zero ISI and long ISIs. Within an operative range, learned responses are timed to match the statistics of the learning environment (e.g., Smith, 1968).

Although the amygdala has been identified as a primary site in the expression of emotion and stimulus-reward associations (Aggleton, 1993), as summarized in Figs. 2 and 5, the hippocampal formation has been implicated in the adaptively timed processing of cognitive-emotional interactions. For example, Thompson et al. (1987) distinguished two types of learning that go on during conditioning of the rabbit Nictitating Membrane Response: adaptively timed “conditioned fear” learning that is linked to the hippocampus, and adaptively timed “learning of the discrete adaptive response” that is linked to the cerebellum. In particular, neurophysiological evidence has been reported for adaptive timing in entorhinal cortex activation of hippocampal dentate and CA3 pyramidal cells (Berger & Thompson, 1978; Nowak & Berger, 1992) to which the more recently reported “time cells” presumably contribute.

Spectral timing has been used to model challenging behavioral, neurophysiological, and anatomical data about several parts of the brain: the hippocampus to maintain motivated attention on goals for an adaptively timed interval (Grossberg & Merrill, 1992, 1996; cf. Friedman, Bressler, Garner, & Ziv, 2000), the cerebellum to read out adaptively timed movements (Fiala, Grossberg, & Bullock, 1996; Ito, 1984), and the basal ganglia to release dopamine bursts and dips that drive new associative learning in multiple brain regions in response to unexpectedly timed rewards and non-rewards (Brown, Bullock, & Grossberg, 1999, 2004; Schultz, 1998; Schultz et al., 1992).

### Distinguishing expected and unexpected disconfirmations

**Fig. 6:**
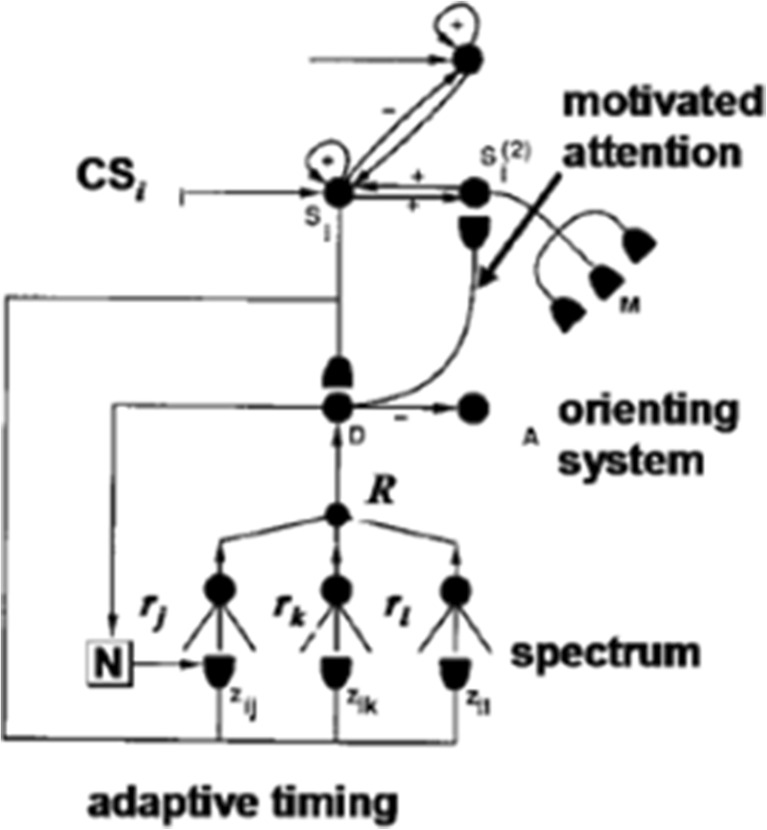
In the START model, conditioning, attention, and timing are integrated. Adaptively timed hippocampal signals *R* maintain motivated attention via a cortico-hippocampal-cortical feedback pathway, at the same time that they inhibit activation of orienting system circuits *A* via an amygdala drive representation *D*. The orienting system is also assumed to occur in the hippocampus. The adaptively timed signal is learned at a spectrum of cells whose activities respond at different rates *r*_*j*_ and are gated by different adaptive weights *z*_*ij*_ *.* A transient Now Print learning signal *N* drives learned changes in these adaptive weights. In the nSTART model, the hippocampal feedback circuit operates in parallel to the amygdala, rather than through it [Reprinted with permission from Grossberg and Merrill (1992)]

Adaptive timing is essential for animals that actively explore and learn about their environment, since rewards and other goals are often delayed in time relative to the actions that are aimed at acquiring them. The brain needs to be dynamically buffered, or protected against, reacting prematurely before a delayed reward can be received. The Spectral Timing model accomplishes this by predicting how the brain distinguishes *expected non-occurrences,* also called *expected disconfirmations*, of reward, which should not be allowed to interfere with acquiring a delayed reward, from *unexpected non-occurrences*, also called *unexpected disconfirmations*, of reward, which can trigger the usual consequences of predictive failure, including reset of working memory, attention shifts, emotional rebounds, and the release of exploratory behaviors. In the nSTART model, and the START model before it, spectral timing circuits generate adaptively timed hippocampal responses that can bridge temporal gaps between CS and US and provide motivated attention to maintain activation of the hippocampus and neocortex between those temporal gaps (Figs. 2 and 6).

What spares an animal from erroneously reacting to expected non-occurrences of reward as predictive failures? Why does an animal not immediately become so frustrated by the non-occurrence of such a reward that it prematurely shifts its attentional focus and releases exploratory behavior aimed at finding the desired reward somewhere else, leading to relentless exploration for immediate gratification? Alternatively, if the animal does wait, but the reward does not appear at the expected time, then how does the animal then react to the unexpected non-occurrence of the reward by becoming frustrated, resetting its working memory, shifting its attention, and releasing exploratory behavior?

Any solution to this problem needs to account for the fact that the process of registering ART-like sensory matches or mismatches is not itself inhibited (Fig. 3): if the reward happened to appear earlier than expected, the animal could still perceive it and release consummatory responses. Instead, the *effects* of these sensory mismatches upon reinforcement, attention, and exploration are somehow inhibited, or *gated off*. That is, a primary role of such an adaptive timing mechanism seems to be to inhibit, or gate, the mismatch-mediated arousal process whereby a disconfirmed expectation would otherwise activate widespread signals that could activate negatively reinforcing frustrating emotional responses that drive extinction of previous consummatory behavior, reset working memory, shift attention, and release exploratory behavior.

The START model unifies networks for spectrally timed learning and the differential processing of expected versus unexpected non-occurrences, or disconfirmations (Fig. 6). In START, learning from sensory cortex to amygdala in S_*i*_ → D pathways is supplemented by a parallel S_*i*_ → H hippocampal pathway. This parallel pathway embodies a spectral timing circuit. The spectral timing circuit supports adaptively timed learning that can bridge temporal gaps between cues and reinforcers, as occurs during trace conditioning. As shown in Fig. 6, both of these learned pathways can generate an inhibitory output signal to the orienting system A. As described within ART (Fig. 3c), the orienting system is activated by novelty-sensitive mismatch events. Such a mismatch can trigger a burst of nonspecific arousal that is capable of resetting the currently active recognition categories that caused the mismatch, while triggering opponent emotional reactions, attention shifts, and exploratory behavioral responses. The inhibitory pathway from D to A in Fig. 6 prevents the orienting system from causing these consequences in response to expected disconfirmations, but not to unexpected disconfirmations (Grossberg & Merrill, 1992, 1996). In particular, read-out from the hippocampal adaptive timing circuit activates D which, in turn, inhibits A. At the same time, adaptively timed incentive motivational signals to the prefrontal cortex (pathway D → S_*i*_^(2)^ in Fig. 6) are supported by adaptively timed output signals from the hippocampus that help to maintain motivated attention, and a cognitive-emotional resonance for a task-appropriate duration.

Thus, in the START model, two complementary pathways are proposed to control spectrally-timed behavior: one excites adaptively-timed motivated attention and responding, and the other inhibits orienting responses in response to expected disconfirmations. Adaptively-timed motivated attention is mediated through an inferotemporal-amygdala-orbitofrontal positive feedback loop in which conditioned reinforcer learning and incentive motivational learning work together to rapidly focus attention upon the most salient cues, while blocking recognition of other cues via lateral inhibition (see Figs. 5 and 6). The hippocampal adaptive timing circuit works in parallel to maintain activity in this positive feedback loop and thereby focus motivated attention on salient cues for a duration that matches environmental contingences.

### nSTART model

The nSTART model builds upon, extends, and unifies the ART, CogEM, and START models in several ways to explain data about normal and abnormal learning and memory. First, nSTART incorporates a simplified model hippocampus and adaptively timed learning within the model's thalamo-hippocampal and cortico-hippocampal connections (Fig. 2). Second, nSTART incorporates a simplified version of ART category learning in its bottom-up cortico-cortical connections. Third, learning in these connections, and in the model's hippocampo-cortical connections, is modulated by a simple embodiment of BDNF. Fourth, the sensory cortical and orbitofrontal cortical processing stages habituate in an activity-dependent way, a property that has previously been used to model other cortical development and learning processes, such as the development of visual cortical area V1 (e.g., Grossberg & Seitz, 2003; Olson & Grossberg, 1998).

The nSTART model focuses on amygdala and hippocampal interactions with the sensory cortex and orbitofrontal cortex during conditioning (Figs. 2 and 6), with the hippocampus required to support learning and memory consolidation, especially during learning experiences such as trace conditioning wherein a temporal gap between the associated stimuli needs to be bridged, as described above. Consolidation is enabled, in the brain and in the model, by a self-organizing process whereby active neurons and specific neural connections are reinforced and strengthened through positive feedback.

BDNF-mediated hippocampal activation is proposed to maintain and enhance cortico-cortical resonances that strengthen and stabilize partial learning based on previously experienced bottom-up sensory inputs. This partial learning occurs during conditioning trials within the bottom-up adaptive filters that activate learned recognition categories, and within the corresponding top-down expectations. After the consolidation process strengthens these pathways, the hippocampus is no longer required for performance of CRs, but rather the prefrontal cortex takes on a critical role in generating successful performance of the CR in concert with the associated thalamic sensory input (Takehara et al., 2003) and amygdala-driven motivational support. Since amygdala and prefrontal cortex provide input to the pontine nuclei, their collective activity there reflects the salience of the CS in generating a trace CR (Siegel et al., 2012; Siegel et al., 2015). The prefrontal cortex interacts with the cerebellum via the pontine nucleus to directly mediate adaptively timed conditioned responses (Weiss & Disterhoft, 2011; Woodruff-Pak & Disterhoft, 2007). A detailed biochemical model of how the cerebellum learns to control adaptively timed conditioned responses is developed in Fiala, Grossberg, and Bullock (1996), with the Ca^++^-modulated metabotropic glutamate receptor (mGluR) system playing a critical role in enabling temporal gaps to be bridged via a spectral timing circuit.

### Linking consciousness, conditioning, and consolidation

The nSTART model traces the link between consciousness and conditioning to cognitive-emotional resonances that are sustained long enough to support consciousness. Such cognitive-emotional resonances maintain *core consciousness* (Damasio, 1999) and the ability to make responses, somatosensory responses in the case of eyeblink conditioning, that depend on interactions between the sensory cortex and orbitofrontal cortex, or thalamus and medial prefrontal cortex (Powell & Churchwell, 2002 **).** The nSTART model proposes that, when the hippocampus is removed, and with it the capacity to sustain a temporally prolonged cognitive-emotional resonance and adaptively timed focusing of motivated attention upon cognitively relevant information, then core consciousness and performance may be impaired. The model hereby explains how interactions among the thalamus, hippocampus, amygdala, and cortex may support the conscious awareness that is needed for trace conditioning, but not delay conditioning (Clark & Squire, 1998).

As explained by the model, memory consolidation during trace conditioning builds upon cooperative interactions among several different neural pathways in which learning takes place during trace conditioning trials. Consider the case of the circuits in Figs. 4 and 5, for example. A property of the CogEM model, which is supported by neurophysiological data, as summarized below, is that the (sensory cortex)→(orbitofrontal cortex) pathway, by itself, is not able to initiate efficient conditioning. Motivational support is needed as well. How this is proposed to occur is illustrated by considering what would happen if the sensory cortex and prefrontal cortex were lumped together, as in Fig. 4a. Then, after a reinforcing cue activated a sensory representation S, it could activate a motor representation M at the same time that it also sent conditioned reinforcer signals to a drive representation D such as the amygdala. As a result, a motor response could be initiated before the sensory representation received incentive motivational feedback to determine whether the sensory cue *should* generate a response at that time. For example, eating behavior might be initiated before the network could determine if it was hungry.

This deficiency is corrected by interactions between a sensory cortex and its prefrontal, notably orbitofrontal, cortical projection, as in Fig. 4b and its anatomical interpretation in Fig. 5. Here, the various sensory cortices play the role of the first cortical stage S_CS_^(1)^ of the sensory representations, the orbitofrontal cortex plays the role of the second cortical stage S_CS_^(2)^ of the sensory representations, and the amygdala and related structures play the role of the drive representations D. This two-stage sensory representation overcomes the problem just mentioned by assuming that each orbitofrontal cell obeys a *polyvalent* constraint whereby it can fire vigorously only if it receives input from its sensory cortex *and* from a motivational source such as a drive representation. This polyvalent constraint on the model prefrontal cortex prevents this region from triggering an action until it gets incentive feedback from a motivationally-consistent drive representation (Grossberg, 1971, 1982). More specifically, presentation of a given cue, or CS, activates the first stage S_CS_^(1)^ of its sensory representation (in sensory cortex) in Fig. 4b. This activation is stored in short-term memory using positive feedback pathways from the sensory representation to itself. The stored activity generates output signals to all the drive representations with which the sensory representation is linked, as well as to the second stage S_CS_^(2)^ of the sensory representation (in prefrontal cortex). The second stage S_CS_^(2)^ obeys the polyvalent constraint: It cannot fire while the CS is stored in short-term memory unless it receives converging signals from the first sensory stage (via the S_CS_^(1)^ → S_CS_^(2)^ pathway) and from a drive representation (via the S_CS_^(1)^ → D → S_CS_^(2)^ pathway).

Early in conditioning, a CS can activate its representation S_CS_^(1)^ in the sensory cortex, but cannot vigorously activate its representation S_CS_^(2)^ in the orbitofrontal cortex, or a drive representation D in the amygdala. A US can, however, activate D. When the CS and US are paired appropriately through time, the conditioned reinforcer adaptive weights in the S_CS_^(1)^ → D pathway can be strengthened. The converging CS-activated inputs from S_CS_^(1)^ and US-activated inputs from D at S_CS_^(2)^ also enable the adaptive weights in the incentive motivational pathway D → S_CS_^(2)^ to be strengthened. After conditioning, during retention testing when only the CS is presented, the two pathways S_CS_^(1)^ → S_CS_^(2)^ and S_CS_^(1)^ → D → S_CS_^(2)^ can supply enough converging input to fire the orbitofrontal representation S_CS_^(2)^ without the help of the US.

These properties are consistent with the following anatomical interpretation. The amygdala and related structures have been identified in both animals and humans to be a brain region that is involved in learning and eliciting memories of experiences with strong emotional significance (Aggleton, 1993; Davis, 1994; Gloor et al., 1982; Halgren, Walter, Cherlow, & Crandall, 1978; LeDoux, 1993). The orbitofrontal cortex is known to be a major projection area of the ventral or object-processing cortical visual stream (Barbas, 1995, 2007; Fulton, 1950; Fuster, 1989; Rolls, 1998; Wilson, Scalaidhem, & Goldman-Rakic, 1993). Cells in the orbitofrontal cortex are sensitive to the reward associations of sensory cues, as well as to how satiated the corresponding drive is at any time (e.g., Mishkin & Aggleton, 1981; Rolls, 1998, 2000). The feedback between the prefrontal and sensory cortical stages may be interpreted as an example of the ubiquitous positive feedback that occurs between cortical regions including prefrontal and sensory cortices (Felleman & Van Essen, 1991; Höistad & Barbas, 2008; Macchi & Rinvik, 1976; Sillito, Jones, Gerstein, & West, 1994; Tsumoto, Creutzfeldt, & Legéndy, 1978; van Essen & Maunsell, 1983). In CogEM, it provides a top-down ART attentional priming signal that obeys the ART Matching Rule. Finally, the CogEM, and nSTART, models are consistent with data suggesting that the ventral prefrontal cortex and the amygdala are involved in the process by which responses are selected on the basis of their emotional valence and success in achieving rewards (Damasio, Tranel, & Damasio, 1991; Passingham, 1997). In particular, Fuster (1989) has concluded from studies of monkeys that the orbitofrontal cortex helps to suppress inappropriate responses. These monkey data are consistent with clinical evidence that patients with injury to orbitofrontal cortex tend to behave in an inappropriate manner (Blumer & Benson, 1975; Liddle, 1994).

### Bridging the temporal gap: The hippocampus does this, not the amygdala

The need to regulate orbitofrontal outputs using drive information puts into sharp relief the problem that the brain needs to solve in order to be capable of trace conditioning, or indeed of any learning wherein there is a temporal gap between the stimuli that need to be associated: If the amygdala cannot bridge the temporal gap between CS and US during trace conditioning, what can? If there were no structure capable of bridging that gap, then either the motivational appropriateness of responding would be sacrificed, or the ability to learn across temporal gaps. As briefly noted above, the nSTART model proposes how the brain solves this problem by using the hippocampus to bridge the temporal gap, using spectrally timed learning and BDNF processes in connections from thalamus and sensory cortex to the hippocampus, combined with learned incentive motivational processes and BDNF in connections from the hippocampus to the neocortex (Fig. 2).

Initially, during trace conditioning, the ISI between the CS and US is too large to be bridged by either the direct (sensory cortex)→(orbitofrontal cortex) pathway or by the indirect (sensory cortex)→(amygdala)→(orbitofrontal cortex) pathway. In other words, by the time the US becomes active, CS-activated signals from the sensory cortex to the amygdala and the orbitofrontal cortex have significantly decayed, so that they cannot strongly drive associative learning between simultaneously active CS and US representations. In contrast, in the manner explicated by the model, the greater persistence afforded by hippocampal adaptive timing enables CS-activated signals via the hippocampus to bridge this ISI. Then, when paired with the US, which can activate its own sensory cortical and orbitofrontal cortical representations, CS-activated associations can begin to form in the (sensory cortex)→(hippocampus)→(orbitofrontal cortex) pathway, and can support feedback from orbitofrontal cortex to the CS representation in sensory cortex, thereby enabling a sustained cognitive-emotional resonance that can support conscious awareness. Model hippocampal neurotrophins extend this temporal interval and enhance the strength of these effects. Once both the sensory cortex and orbitofrontal cortex are simultaneously active, associations can also start to form directly from the CS-activated object category representation in the sensory cortex to the orbitofrontal cortex, thereby consolidating the learned categorical memory that associates an object category with an object-value category. As these direct connections consolidate, the hippocampus becomes less important in controlling behaviors that are read out from orbitofrontal cortical sites.

After partial conditioning gets learning started in associated thalamo-cortical and cortico-cortical pathways, during the memory consolidation process, hippocampal adaptively timed circuits, and even beyond that, BDNF activity, persist and support resonating cortico-cortical and cortico-hippocampo-cortical activity. The polyvalent constraint on the firing of orbitofrontal cells is therefore achieved even after learning trials cease. Without hippocampal support after partial conditioning, this cannot occur. The model suggests that this is why early, but not late, hippocampal lesions interfere with the formation and consolidation of conditioned responses.

### Model description

#### nSTART model overview

**Fig. 7:**
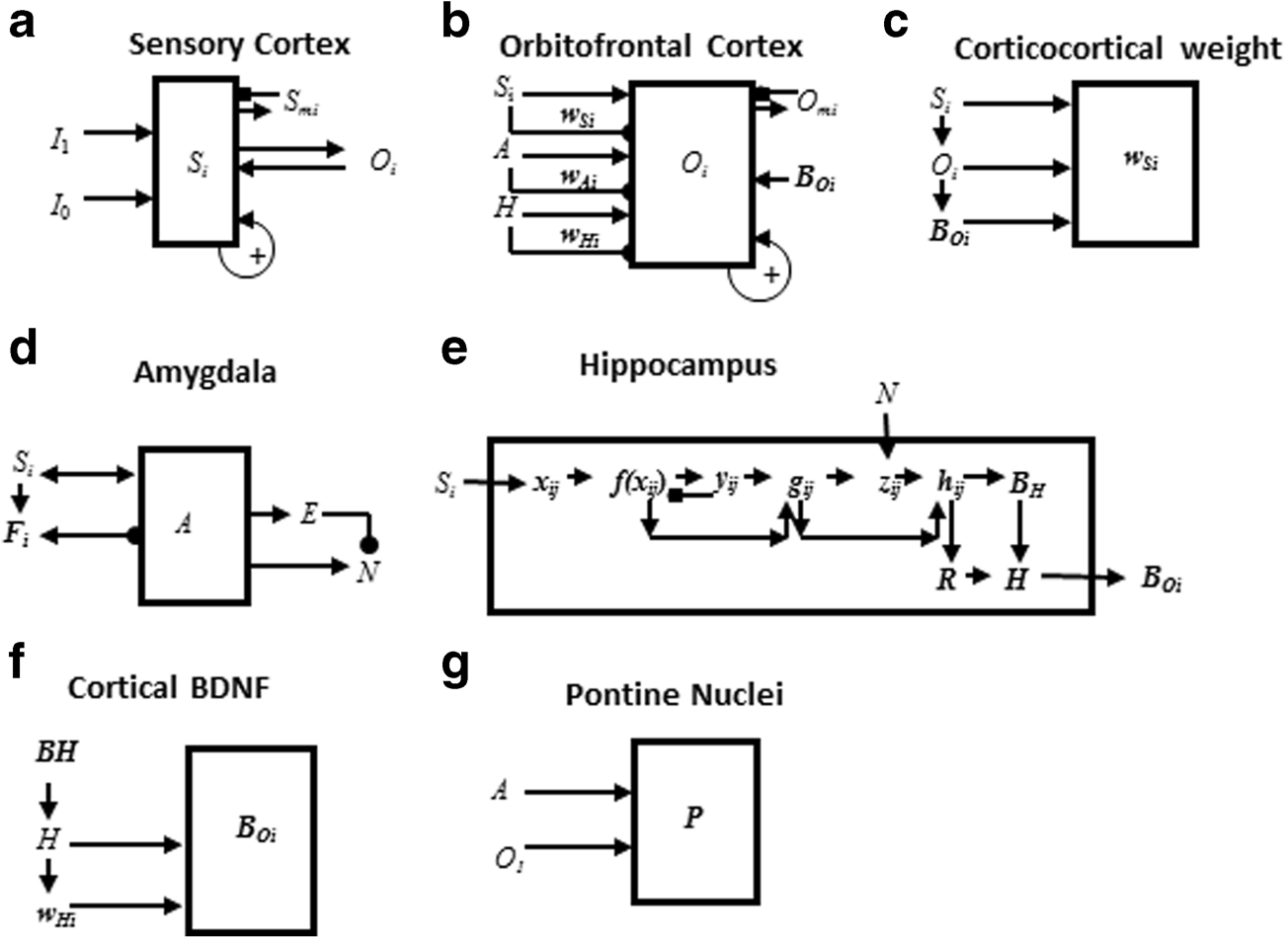
The processing steps for a conditioning trial in the nSTART model are illustrated. Conditioned variables that represent learning are not reset to zero between trials in order to simulate inter-trial learning. These include adaptive weights *w*_*Si*_, *w*_*Ai*_, *w*_*Hi*_, *F*_*i*_, and *z*_*ij*_; and hippocampal and orbitofrontal brain-derived neurotrophic factor (BDNF) *B*_*H*_ and *B*_*Oi*_, respectively. (**a**) External stimuli, *I*_***i,***_ activate sensory representations in the sensory cortex *S*_*i*_ via the thalamus *T*_*i*_. Orbitofrontal cortical activity *O*_*i*_ generates a top-down excitatory feedback signal back to *S*_*i*_. The total excitatory signal, including this positive feedback, is gated by the habituative transmitter gate *S*_*mi*_. (**b**) Excitatory inputs to orbitofrontal cortex from sensory cortex (*S*_*i*_), amygdala (*A*), and hippocampus (*H*) are gated by learned presynaptic weights (*w*_*Si*_, *w*_*Ai*_, and *w*_*Hi*_, respectively). An example of this processing is shown in Fig. 7c. Orbitofrontal BDNF (*B*_*Oi*_) extends the duration of *O*_*i*_ activity. The total excitatory signal, including positive feedback, is gated by the habituative transmitter gate *O*_*mi*_. (**c**) The learned weight *w*_*Si*_ from sensory cortex to orbitofrontal cortex is modulated by orbitofrontal and BDNF signals. (**d**) Amygdala (*A*) receives inputs from sensory cortex (*S*_*i*_) that are gated by conditioned reinforcer adaptive weights (*F*_*i*_). The transient Now Print signal (*N*) that drives the learning of adaptively timed hippocampal responses is the difference between the excitatory signal from amygdala (*A*) and an inhibitory signal from a feedforward amygdala-activated inhibitory interneuron (*E*), which time-averages amygdala activity. (**e**) Sensory cortical (*S*_*i*_) inputs to hippocampus (*H*) learn to adaptively time (*z*_*ij*_) the inter-stimulus interval (ISI) using the Now Print signal (*N*) to drive learning within a spectral timing circuit. The cells in the spectral timing circuit react to sensory cortical (*S*_*i*_) inputs at 20 different rates that are subscripted with *j*. The resulting activations (*x*_*ij*_) generate sigmoidal output signals (*f*(*x*_*ij*_)). These outputs are multiplied by their habituative transmitter gates (*y*_*ij*_) to produce an activation spectrum (*g*_*ij*_) which determines the rate at which the adaptive weights (*z*_*ij*_) learn from *N*. The *z*_*ij*_ multiply the *g*_*ij*_ to generate net outputs *h*_*ij*_ that are added to generate an adaptively timed population input (*R*) to hippocampus (*H*). R also regulates hippocampal BDNF (*B*_*H*_), which further extends hippocampal activity through time. *H* also supports production of orbitofronal BDNF (*B*_*Oi*_). (**f**) Hippocampal BDNF (*B*_*H*_) is an indirect promoter of the production of cortical BDNF (*B*_*Ci*_) through its excitatory effect on the activity *H*. (g) Pontine nuclei (*P*) are excited by amygdala (*A*) and orbitofrontal cortex (*O*) and are the model’s final common pathway for generating a CR. These processing components are combined in Fig. 18

The nSTART model is here described in terms of the processing stages that are activated during a conditioning trial, and the functional role of each stage is explained. Fig. 2 illustrates the model as a macrocircuit. Figure 7 shows a set of diagrams that summarize the processing steps and relationships among the model variables. Below they are combined to form a complete circuit diagram (Fig. 18) for which mathematical equations and parameters are also specified. Model parameters have the same values for all simulations except where modifications have been made to simulate lesions or different US levels.

**Fig. 8:**
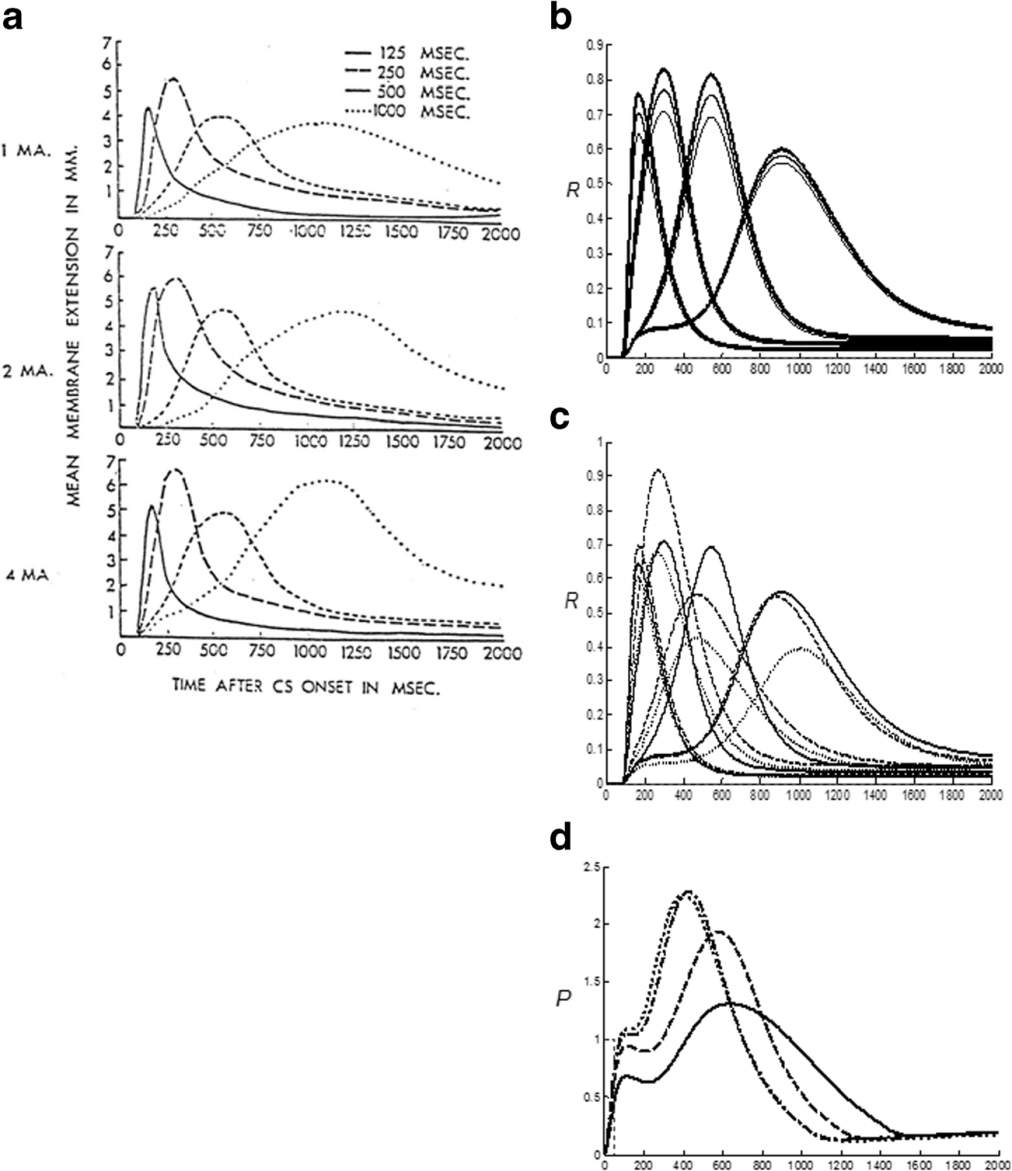
(**a**) Data showing trace conditioning data at multiple inter-stimulus intervals (ISIs) for different unconditioned stimulus (US) levels (Smith, 1968). (**b**) Simulation of Smith data by nSTART model is based on 20 acquisition trials per ISI for time = 1 to 2,000 ms, US level =1 (solid line), 2 (thicker solid line), and 4 (thickest solid line). The hippocampal output signal *R* (Eq. 17) is plotted for a retention test trial in response to the conditioned stimulus (CS) alone. Simulating qualitative properties of the data, peak amplitude of each curve is near its associated ISI of 125, 250, 500, and 1,000 ms, respectively. The model is sensitive to US intensity. (**c**) A comparison of the normal simulation of the Smith data in (**b**) using US level =1 (solid line), with simulation of two abnormal treatments: with no hippocampal brain-derived neurotrophic factor (BDNF) (dashed-line) and with no hippocampal BDNF and no cortical BDNF (dotted-line). Short ISIs show an increase in amplitude, longer ISIs show a decrease. (**d**) Activity in the pontine nuclei (*P*) for a retention test in response to the CS only: ISI = 125 ms (dotted line), ISI = 250 ms (dotted-dashed line), ISI = 500 ms (dashed line), ISI = 1,000 ms (solid line). The CS input is shown as a vertical dashed bar starting at a CS onset at 1 ms. Short ISIs (125 ms and 250 ms) do not exhibit typical pontine profiles; *in vivo*, very short ISIs are likely processed directly by the pons and its connection to the cerebellum. As the ISI becomes longer and a conditioned response (CR) is more reliant on the timed orbitofrontal connection to the pons, pontine activity matches the experimental data

**Fig. 9:**
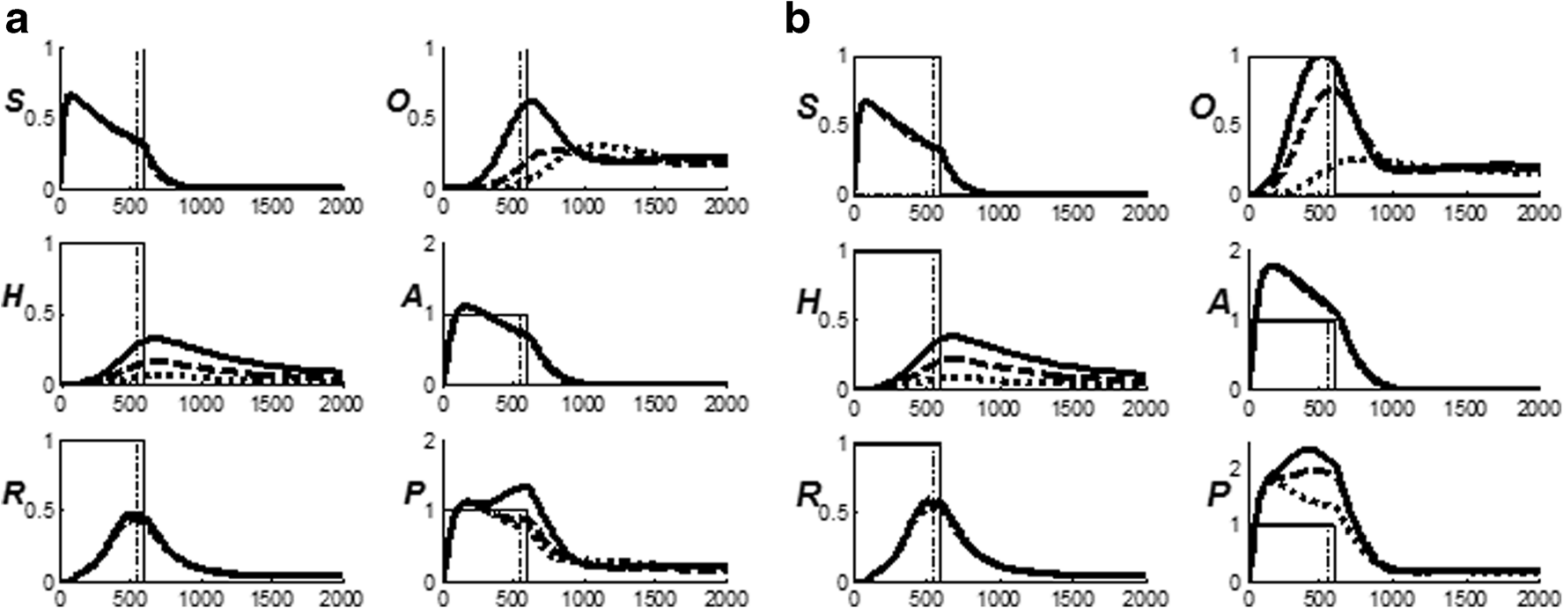
The hippocampus is not required for delay conditioning. (**a**) To simulate hippocampal lesions before any delay conditioning trials, the scalar *β*_*H*_ in the hippocampus excitation term in Eq. 16 was progressively decreased. There were five training trials with US onset at 550 ms, US duration = 50 ms, US offset at 600 ms, and US level = 1. The results show network activations in response to a CS after training: sensory cortex (*S*), orbitofrontal cortex (*O*), hippocampus (*H*), amygdala (*A*), hippocampal adaptive timing (*R*), and the pontine nuclei (*P*). The CS is represented by vertical solid lines, the US onset during training by a vertical dashed line (in delay conditioning, the CS offset and the US offset coincide). Delay conditioning shows little change in pontine activity in the normal (solid line) versus 50 % (dashed line) and 80 % (dotted line) lesions. (**b**) Ten learning trials, instead of the five trials in (**a**), yield better learning, including at the orbitofrontal cortex

**Fig. 10:**
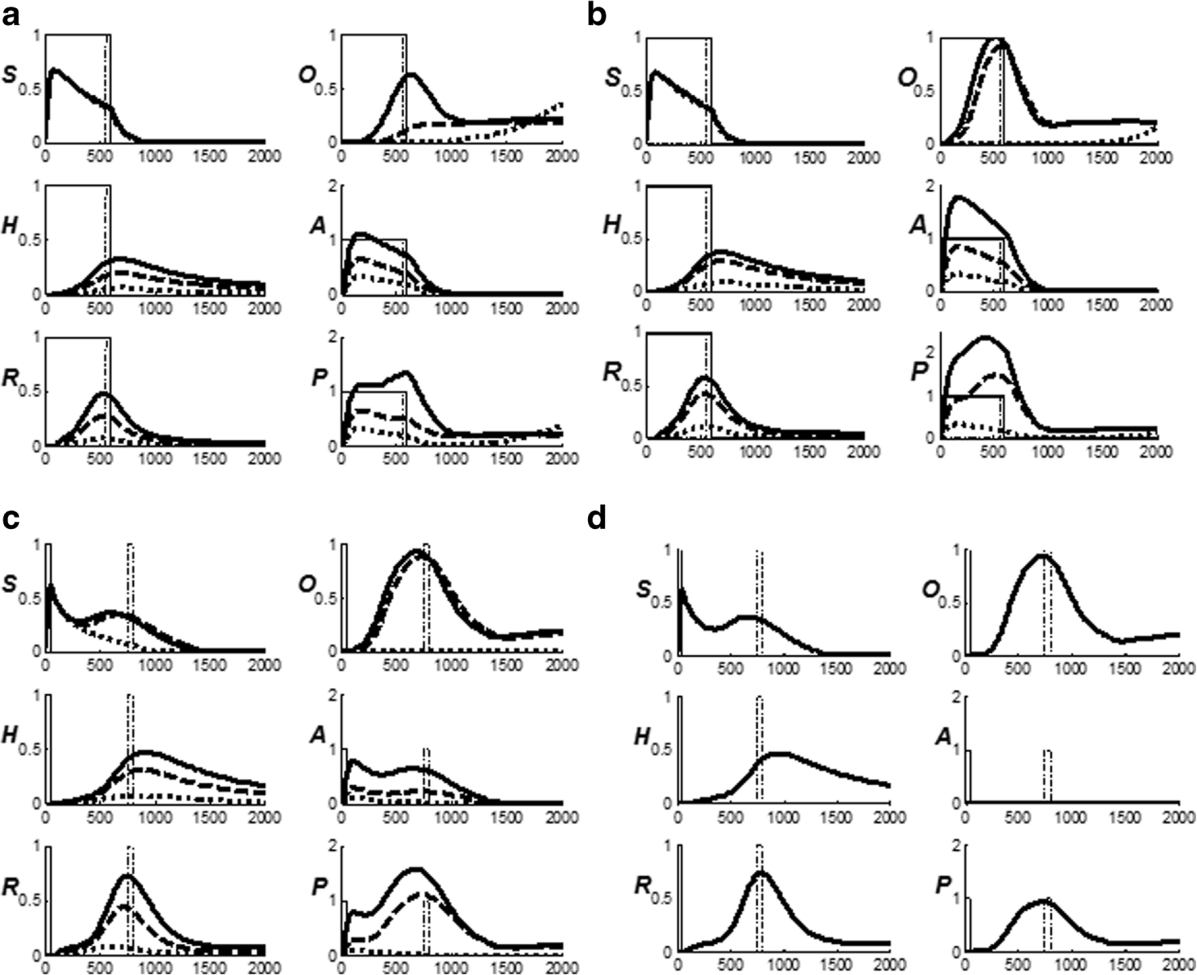
Simulations of amygdala lesions demonstrate that the amygdala is required for optimal acquisition but not for successful retention. (**a**) To simulate partial lesions of the amygdala before any training trials occur in delay conditioning (five training trials; unconditioned stimulus (US) onset at 550 ms, US duration = 50 ms, US offset at 600 ms, US level = 1), scalar *β*_*A*_ in the amygdala excitation term in Eq. 14 was progressively decreased. The results based on the conditioned stimulus (CS)-only presentation during retention testing are presented on a single graph of the variables for sensory cortex (*S*), orbitofrontal cortex (*O*), hippocampus (*H*), amygdala (*A*), hippocampal adaptive timing (*R*), and pontine nuclei (*P*): normal (solid line), 25 % decrease (dashed line), and 50 % decrease (dotted line). These graphs show a marker for the US presented in training for reference only (vertical dashed lines). The CS is also represented (vertical solid lines). Accurate conditioned response (CR) peak amplitude timing as measured by R remained consistent in all cases as *in vivo* but require additional training for improved responses (see Fig. 10b). The activity profiles of the pontine nuclei vary with the strength and timing of cortical activity to effect a CR. *In vivo* they are supplemented by learning in the cerebellum, where an adaptively-timed association is made between signals from the tone CS pathway from auditory nuclei to the pons, and from the pons via mossy fiber projections to the cerebellum, where they are trained by signals from the reflex US pathway from the trigeminal to inferior olive nuclei and then via climbing fibers to the cerebellum (Christian & Thompson, 2003; Fiala, Grossberg, & Bullock, 1996). (**b**) Simulation after ten delay conditioning training trials after partial lesions of the amygdala. All other input parameters and output variables are the same as in Fig. 10a. The CR peak amplitude improved as measured by R. Again, the activity profiles of the pontine nuclei vary with the strength and timing of cortical activity. (**c**) Simulation of partial lesions of the amygdala before any training trials occur in trace conditioning (20 training trials, US onset at 750 ms, US duration = 50 ms, US level = 1) show that both the CR amplitude and timing as measured by *R* and *P* are negatively impacted: normal (solid line), 25 % decrease (dashed line), and 50 % decrease (dotted line). The activity profiles of the pontine nuclei (*P*) reflect the experimental data that amygdala is important in trace conditioning. (**d**) Trace conditioning with amygdala (*A*) ablated 100 % after 20 acquisition trials but just before the retention test. On retention test with CS only, normal activity profiles for CS and US in sensory cortex (*S*) and orbitofrontal cortex (*O*) support normal adaptively-timed response in hippocampus (*R*), indicating a time-limited involvement of the amygdala during acquisition. The activity profile of the pontine nuclei (*P*) also supports the simulation of the data that amygdala involvement is time-limited

**Figure:**
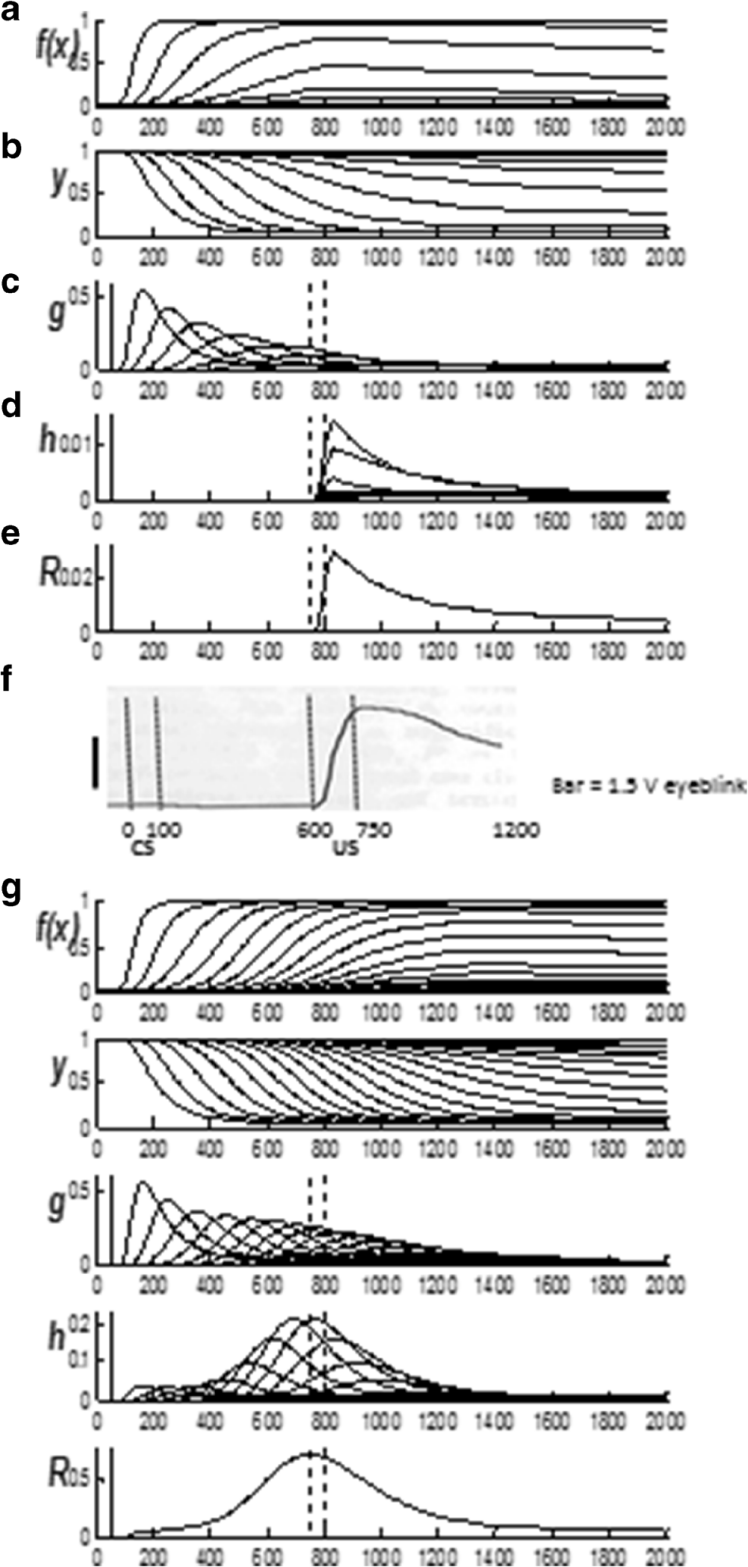

**Fig. 12:**
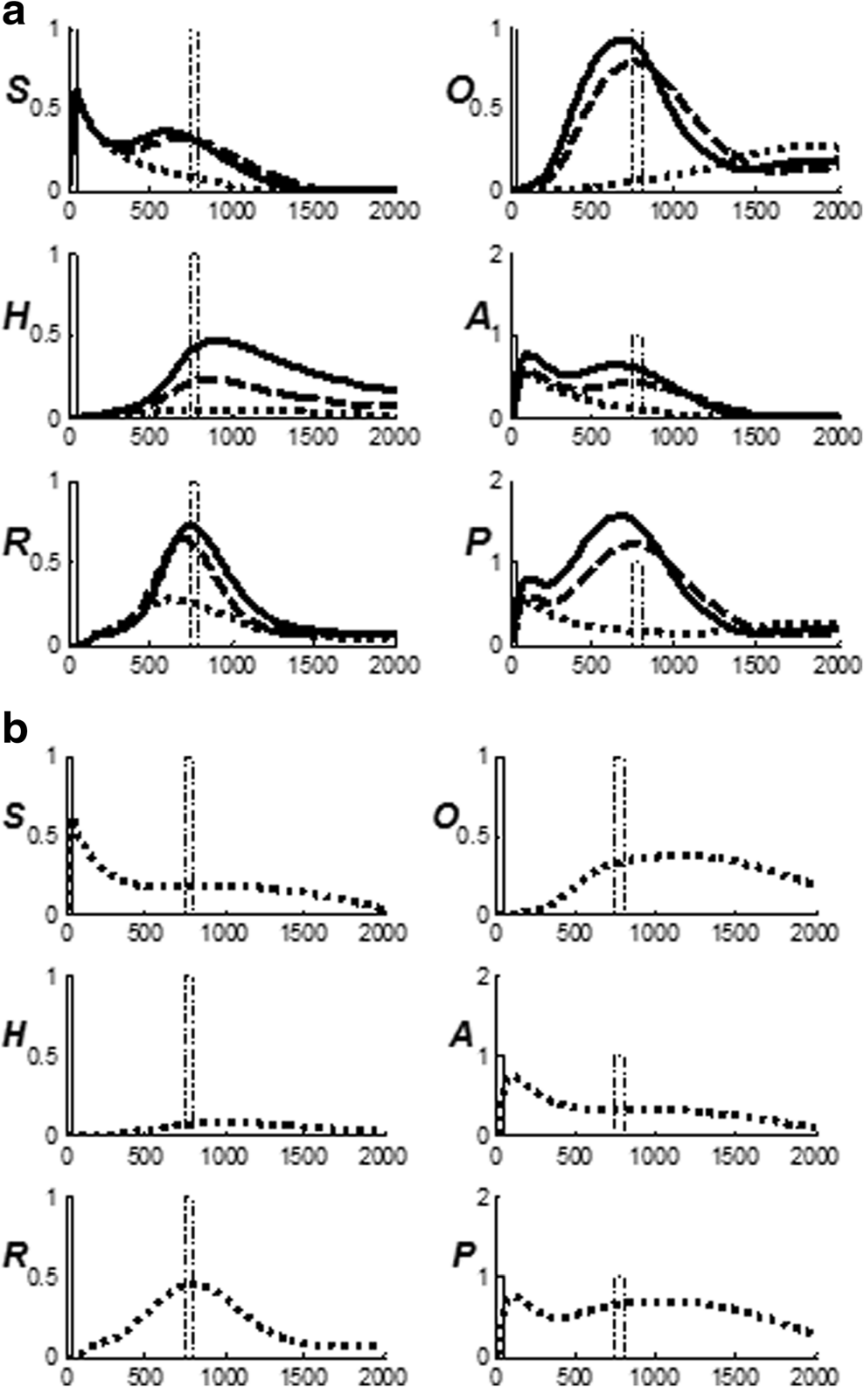
Optimal trace conditioning depends on adequate hippocampus function. (**a**) To simulate partial lesions of the hippocampus before any training trials occur in trace conditioning, scalar *β*_*H*_ in the hippocampal excitation term in Eq. 16 was progressively decreased. This was followed by 20 training trials, with unconditioned stimulus (US) onset at 750 ms, US duration = 50 ms, and US amplitude = 1. The results of retention testing are shown for the activities of sensory cortex (*S*), orbitofrontal cortex (*O*), hippocampus (*H*), amygdala (*A*), hippocampal adaptive timing (*R*), and the pontine nuclei (*P*). These graphs show a marker for the US presented in training for reference only (vertical dashed lines). The conditioned stimulus (CS) is also represented (vertical solid lines). Compared with normal retention testing results after 20 acquisition trials results (solid line), a 50 % decrease (dashed line) gave a small reduction in conditioned response (CR) peak amplitude and retained good timing while an 80 % decrease (dotted line) caused deficits in both amplitude and timing. (**b**) While extended training (60 trials rather than 20) with 80 % ablation shows minor improvement in the amplitude and timing of *R*, the amplitude and timing of *P* remain too small to support a normal CR. An intact hippocampus is thus required for efficient trace conditioning

**Figure:**
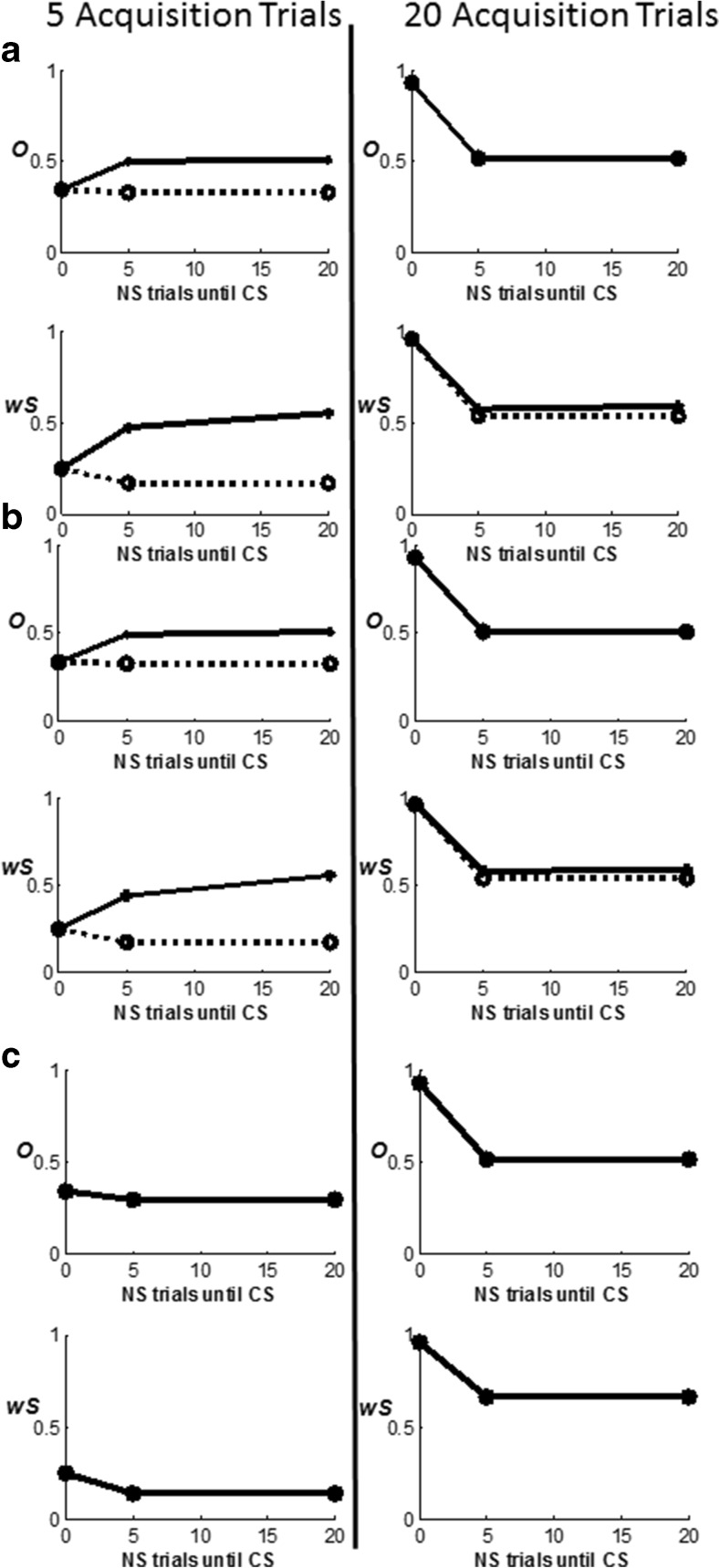

**Fig. 14:**
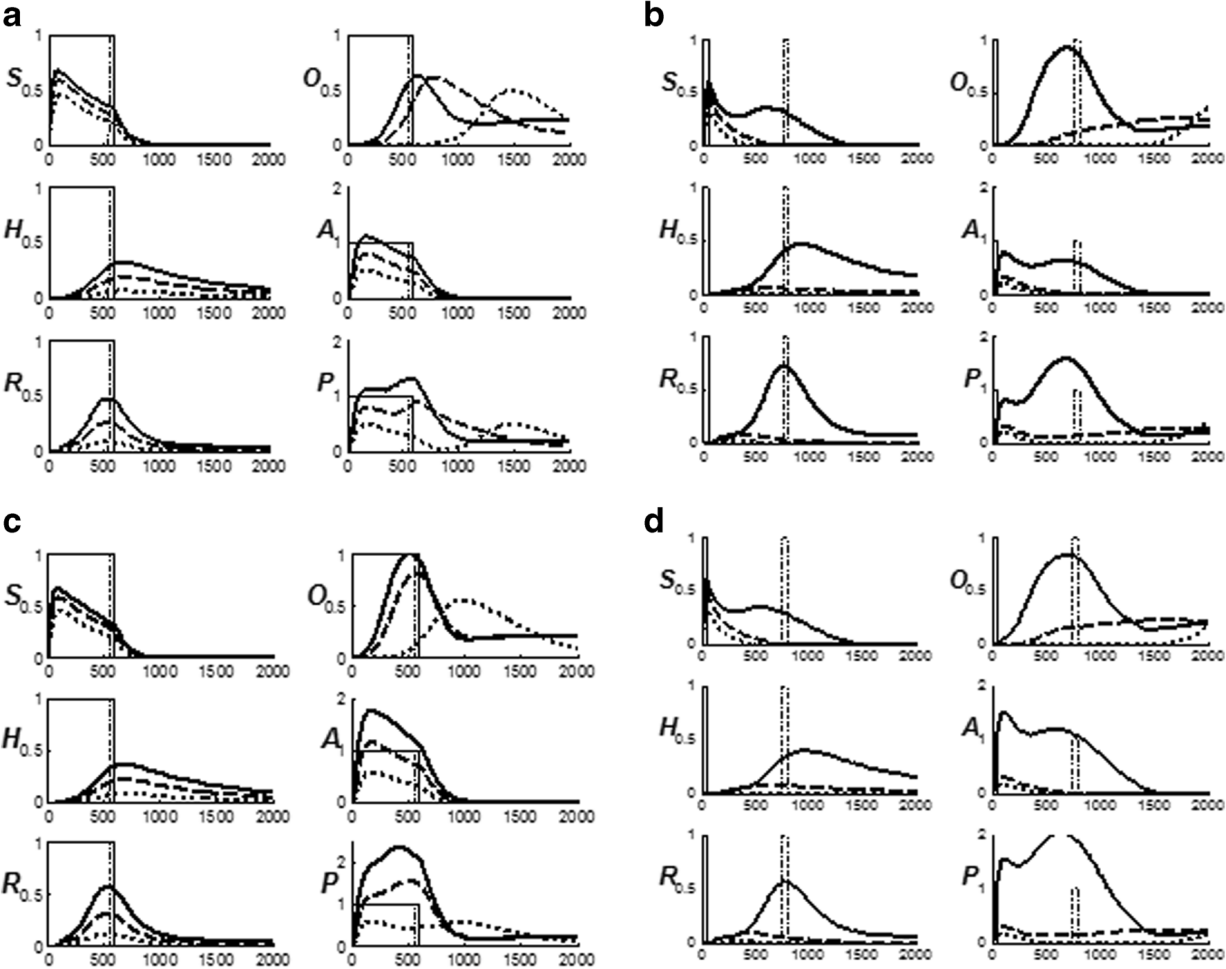
Simulations of lesions of the thalamus, with equivalent effects on sensory cortex, demonstrate that the sensory cortex is required for optimal acquisition and retention in both delay and trace conditioning. To simulate partial lesions of the sensory cortex before any training trials occur, scalar *β*_*S*_ in the sensory cortex (Eq. 2) was progressively decreased: normal = solid line, 25 % decrease = dashed line, and 50 % decrease = dotted line. The results of retention testing by conditioned stimulus (CS) presentation are shown for sensory cortex (*S*), orbitofrontal cortex (*O*), hippocampus (*H*), amygdala (*A*), hippocampal adaptive timing (*R*), and the pontine nuclei (*P*). Vertical dashed lines mark the time of unconditioned stimulus (US) presentation during training, but not recall, trials. Vertical solid lines mark the onset and offset of the CS during training trials. Lesions to the sensory cortex weaken learning as a function of the conditioning paradigm and the extent of the lesion, with a special focus on O and P. (**a**) Recall after five training trials of delay conditioning in all three cases. (**b**) Worse trace conditioning was seen in the lesioned cases, even after 20 training trials, than in the corresponding delay conditioning cases in (**a**). (**c**) Doubling the number of training trials during delay conditioning to ten training trials improved performance in all three cases. (**d**) Doubling the number of training trials during trace conditioning to 40 trials improved performance in the no-lesion case, but had a negligible effect in the two lesioned cases

**Fig. 15:**
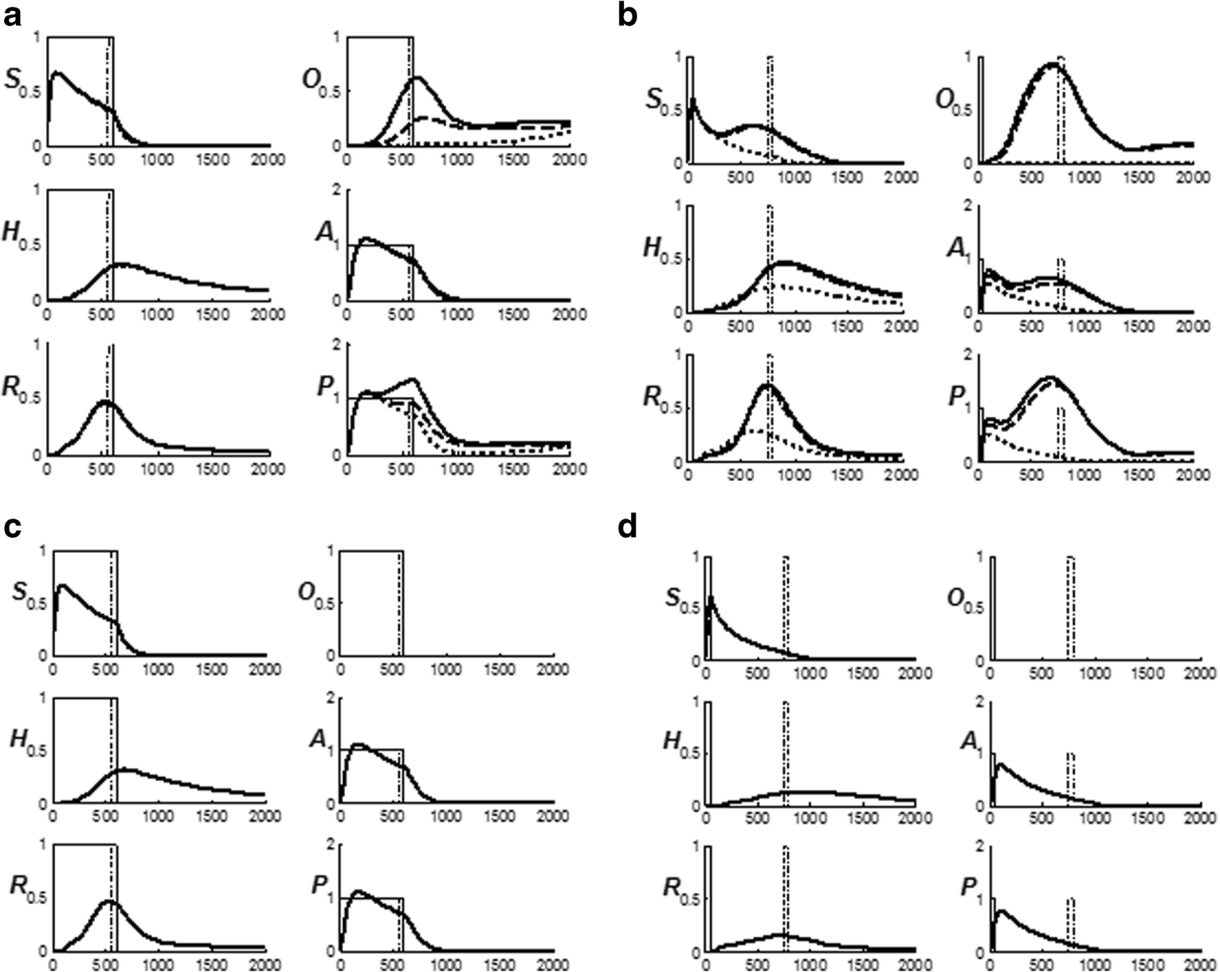
Pre-training orbitofrontal cortical lesions do not impair delay conditioning as much as trace conditioning. Scalar *β*_*O*_ in the orbitofrontal cortex (Eq. 7) was progressively decreased to simulate a lesion. In (**a**) and (**b**), the unlesioned normal case = solid line, 5 % lesion = dashed line, and 10 % lesion = dotted line. The conditioned stimulus (CS) and unconditioned stimulus (US) inputs were chosen as in Fig. 14. The results of retention testing due to CS presentation are shown by graphing the activities of sensory cortex (*S*), orbitofrontal cortex (*O*), hippocampus (*H*), amygdala (*A*), hippocampal adaptive timing (*R*) and pontine nuclei (*P*): (**a**) Delay conditioning with five acquisition trials. (**b**) Trace conditioning with 20 acquisition trials. (**c**) Complete lesions after delay conditioning with five acquisition trials do not impact the ability to perform the conditioned response (CR) as reflected in *R* and *P* amplitudes, although timing of P is impaired. (**d**) Complete orbitofrontal lesions after trace conditioning with 20 acquisition trials greatly reduce the ability to perform the CR as reflected in collapsed *R* and *P* amplitudes, and a failure of *P* timing. Thus orbitofrontal cortex is required for performance after trace conditioning in the data and the model

**Figure:**
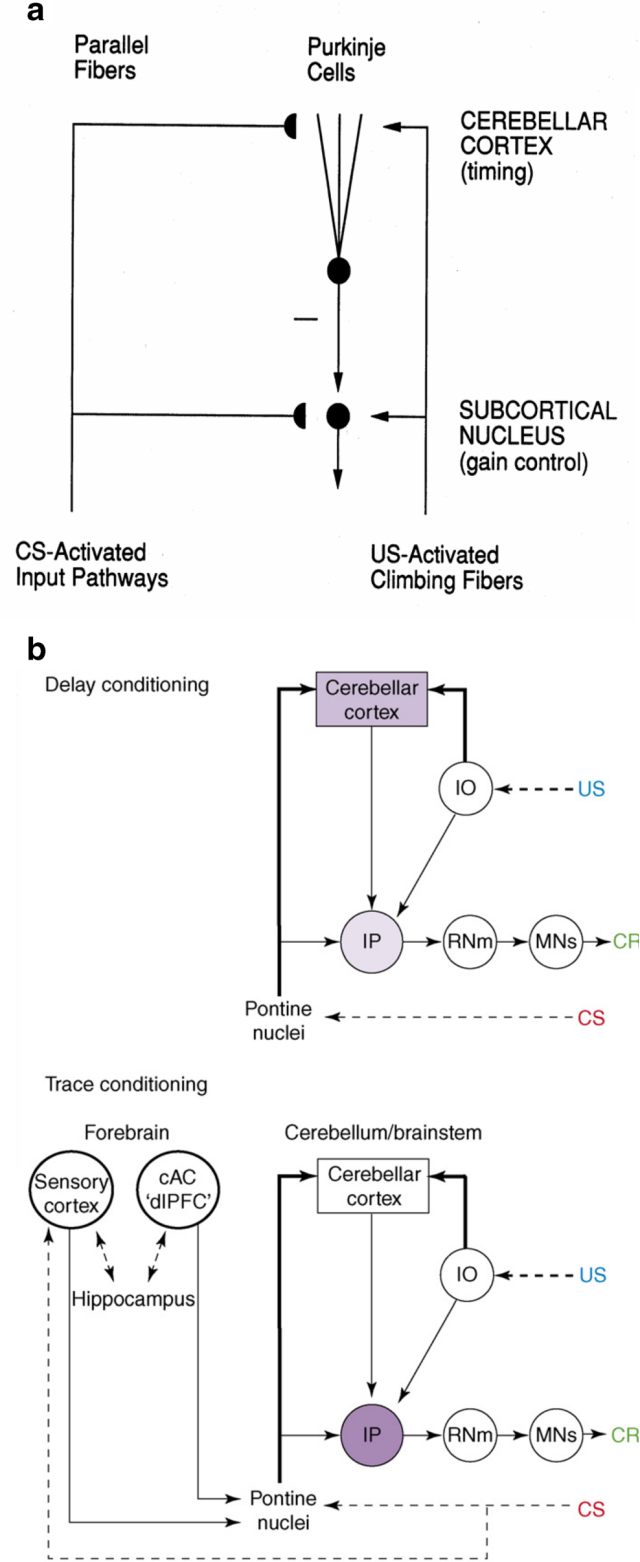

**Fig. 17:**
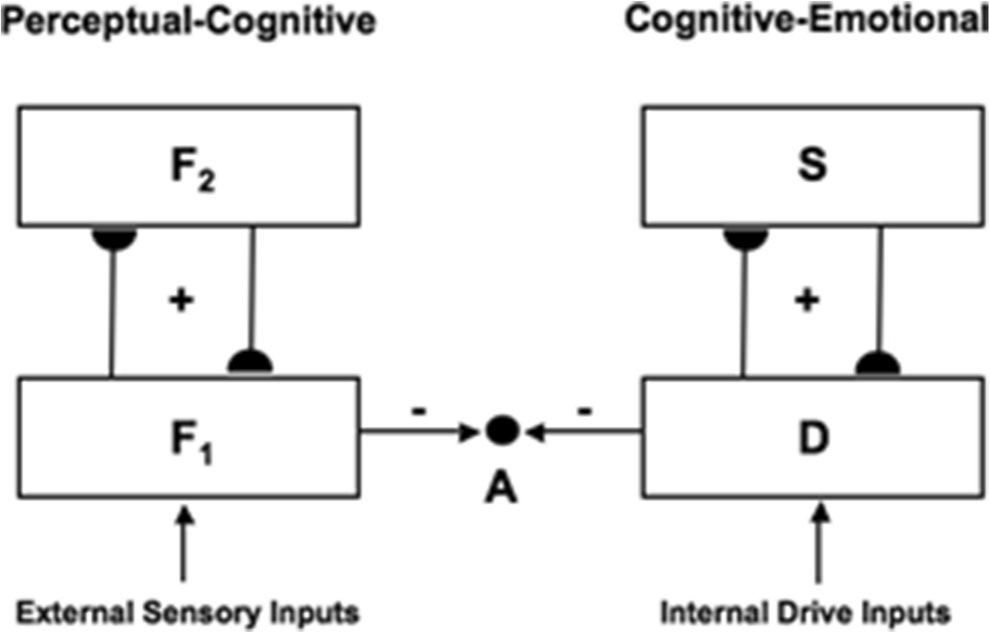
In the START model framework, ART category learning circuits and Spectral Timing circuits can both inhibit the orienting system: When a good enough match occurs between a feature pattern at level *F*_1_ and the top-down expectation from the category level *F*_2_, inhibition can occur of the orienting system *A*, thereby preventing a memory search. If inhibition from the cognitive-emotional sensory-drive (*S* − *D*) resonance that is supported by hippocampal adaptive timing also inhibits *A*, then the orienting system again cannot fire until the adaptively timed signal is removed. The former mechanism clarifies how hippocampal novelty potentials fade away as thalamo-cortical and cortico-cortical category learning consolidates. The latter mechanism clarifies how orienting responses are inhibited during expected disconfirmations

**Fig. 18:**
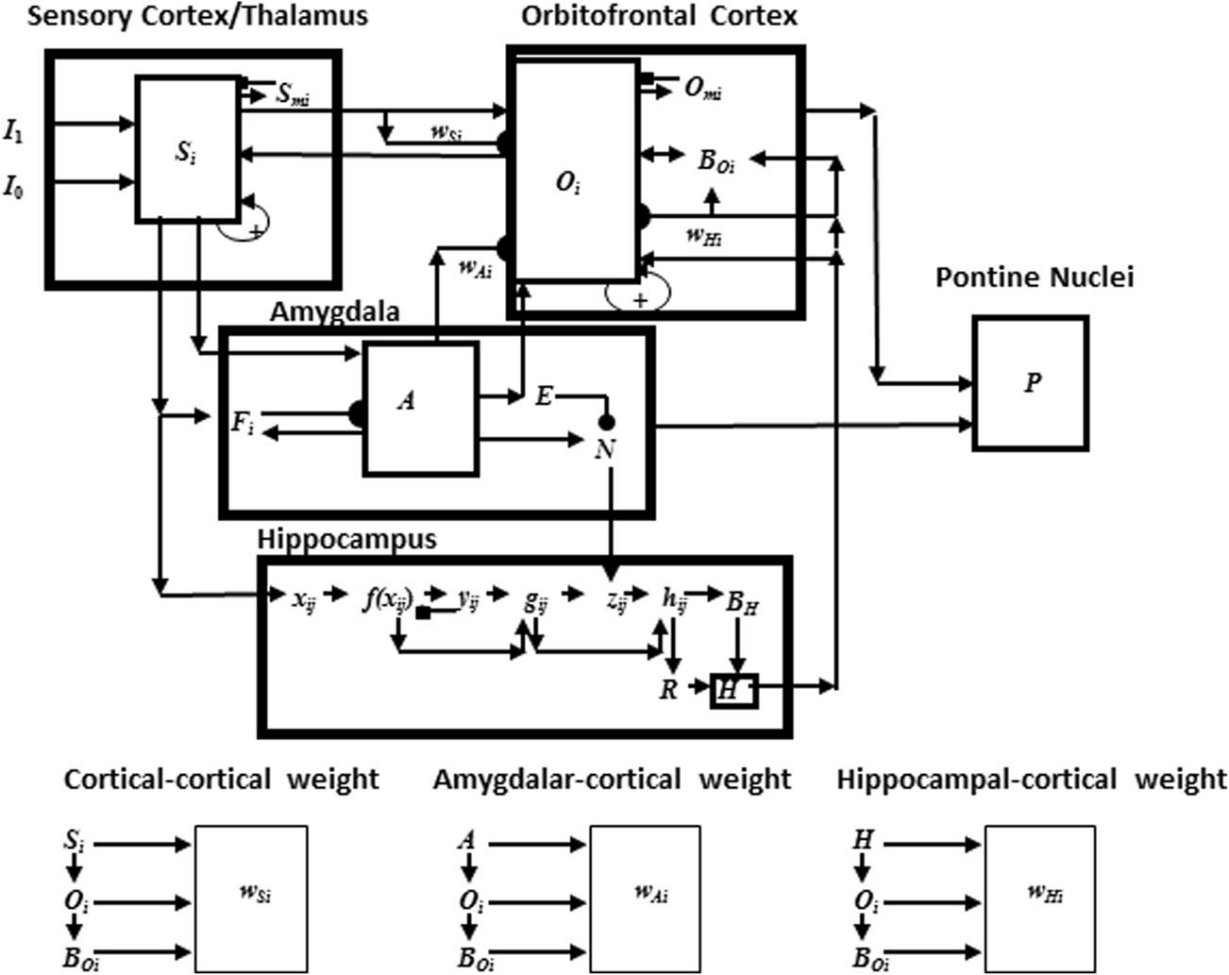
Interacting thalamic, prefrontal cortical, amygdala, and hippocampal processing circuits control adaptively timed responses in conditioning acquisition and maintenance. The circuit diagram is a composite of the macrocircuit structure given in Fig. 2 and the processing detail given in Fig. 7. The text contains the mathematical definitions of the circuit variables

For each trial, conditioning variables are simulated from 1 to 2,000 ms. Three types of trials simulate the learning of conditioning contingencies: acquisition or training (CS-US pairing), retention or testing (CS only), and no stimulus (neither CS nor US) in order to extend the time between the last training trial and the testing trial. Between any two trials, process variables are either reset to initial values, or not, depending on their functional role. There are two types of process variables: one for intra-trial process dynamics (these variables are reset for each trial), and one for inter-trial cumulative learning (these variables are not reset for each trial). Cumulative learning variables are identified below in the discussion of the functional role of each process. See Table 2 for a list of all variables.

#### Sensory cortex and thalamus

##### Sensory cortical dynamics

The dynamics of sensory cortex were simulated (Fig. 2). Thalamic activity was set equal to the resultant sensory cortical activity, for computational simplicity. CS and US inputs are labeled *I*_1_ and *I*_0_, respectively. Input *I*_*i*_ activates the *i* ^*th*^sensory cortical cell, *i =* 0 or 1. The inputs are turned on and off through time by presentation and termination of a CS input (*I*_1_) or US input (*I*_0_), and are defined by a saturating function *I* = *f*(*σ*) = 16*σ*/(1+3*σ*) of an external stimulus intensity *σ*.

Sensory cortex cell activities *S*_*i*_ compete for a limited capacity of activation via a recurrent on-center off-surround network of cells that obey membrane, or shunting equations. (see Eqs. 1 and 2 below). These recurrent interactions use a nonlinear signal function (see Eq. 4) that contrast-enhances network activity patterns and sustains the contrast-enhanced activities in short-term memory after the input pattern ends. In addition to the bottom-up input *I*_*i*_ and the recurrent on-center interactions, excitatory inputs include a top-down attentional signal *O*_*i*_ from object-value categories in the orbitofrontal cortex. This feedback pathway closes a bottom-up/top-down feedback loop between sensory cortex and orbitofrontal cortex and gain-amplifies cortico-cortical activity (see Eq. 7).

A habituative transmitter gate *S*_*mi*_ multiplies the total excitatory input and is inactivated by it in an activity-dependent way, thereby preventing unlimited perseverative activation of the cortico-cortical excitatory feedback loop (see Eq. 6). This gate can be realized in several ways, one being a presynaptic chemical transmitter that is released by axonal signals, and the other a postsynaptic membrane current. The orbitofrontal cortical cells have an analogous habituative process (see Eq. 13). When all these processes interact, a brief input can trigger sustained cortical activity via the recurrent on-center, modulated by orbitofrontal attentional feedback, until it habituates in an activity-dependent way, or is reset by recurrent competitive interactions.

##### Signal functions in the recurrent on-center off-surround network

In order to suppress noise in the system and contrast enhance cell activity, the signal function *f*_*S*_(*S*_*i*_) in the recurrent on-center off-surround network is faster-than-linear (Grossberg, 1973, 1980), with a firing threshold that is larger than the passive equilibrium point, and grows linearly with cell activity above threshold (see Eq. 4).

##### Habituative transmitter gates

The habituative transmitter gate at each sensory cortical cell accumulates at a constant rate up to a maximum value, and is inactivated at a rate proportional to the size of the excitatory signal that it gates, multiplied by the amount of available transmitter (see Eq. 6; Abbott et al., 1997; Grossberg, 1968b, 1972b, 1980).

#### Orbitofrontal cortex, category learning, and incentive motivational learning

##### Orbitofrontal cortical dynamics

Sensory cortical activity *S*_1_ can generate excitatory signals to cells with orbitofrontal cortical activity *O*_1_. As in the sensory cortex, orbitofrontal cortical cells compete via a recurrent on-center off-surround network the cells of which obey the membrane, or shunting, equations of physiology. These recurrent dynamics enable orbitofrontal cortical activity to contrast-normalize and contrast-enhance its inputs, and for cell activities that win the competition to persist in short-term memory after inputs terminate. Finally, again as in the model sensory cortex, the total excitatory input to prefrontal cortical cells can habituate in an activity-dependent way (see Eq. 13).

##### Cortical category learning and incentive motivational learning

Adaptive weights *w*_*S*1_ exist in the pathway from CS-activated sensory cortex to orbitofrontal cortex, and may be strengthened by the conditioning process. These adaptive weight changes constitute the model's category learning process, and are critical events that enable conditioned responding to occur after sufficient memory consolidation occurs, so that hippocampal support is no longer required.

Before conditioning occurs, when a CS is presented it can activate its sensory representation, and sends signals to its orbitofrontal representation, the amygdala, and the hippocampus. However, before conditioning occurs, these signals cannot vigorously activate other regions of the model network. When the US occurs, it can activate its own sensory and orbitofrontal cortical representations, as well as the amygdala and hippocampus. Incentive motivational signals from the amygdala and hippocampus can then be broadcast nonspecifically to many orbitofrontal cortical cells, including those that receive signals from the CS. The hippocampal incentive motivational signals last longer than the amygdala signals because of their capacity for adaptively-timed responding across long ISIs, as will be noted below. Only those orbitofrontal cortical cells that receive a simultaneous combination of CS-activated and US-activated signals can start to vigorously fire.

When *O*_1_ becomes active at the same time that signals from *S*_1_, are active, the adaptive weight *w*_*S*1_ in the corresponding category learning pathway to orbitofrontal cortex (see Eq. 9) can grow. Category learning enables a CS to activate an orbitofrontal representation that can release conditioned responses further downstream. As in the START model, the sensory cortex (see Eq. 2), amygdala (Eq. 14), and hippocampus (Eq. 16) all play a role in this cortico-cortical category learning process, during which incentive motivational learning from both the amygdala and the hippocampus to the orbitofrontal cortex also takes place, with adaptive weights *w*_*Ai*_ and *w*_*Hi*_ in the corresponding pathways.

After being gated by its adaptive weight *w*_*S*1_, a sensory cortical input to an orbitofrontal cell is multiplicatively modulated, or gated, by the sum of amygdala, hippocampal, and BDNF incentive motivational signals (*A*, *H* and *B*_*O,*_ respectively). As noted above, when these converging signals are sufficiently large at the beginning of conditioning, *O*_1_ can become active, so all three types of adaptive weights abutting the prefrontal cortical cell, from sensory cortex, amygdala, and hippocampus (*w*_*Si*_, *w*_*Ai*_, *w*_*Hi*_), can be conditioned if their input sources are also active at these times (see Fig. 7b and c). In situations where the ISI is large, as during trace conditioning, the incentive motivational signal from the hippocampus may be large, even if the signal from the amygdala is not.

As explained below, the hippocampus can maintain its activity for an adaptively-timed duration that can span a long trace interval. In addition, BDNF at the hippocampus *B*_*H*_ and orbitofrontal cortex *B*_*Oi*_ can sustain prefrontal cortical activity for an even longer duration. This action of BDNF captures in a simplified way how BDNF-modulated hippocampal bursting is maintained during memory consolidation.

These adaptive weights all obey an *outstar learning* law (Grossberg, 1968a, 1969, 1980). In the incentive motivational pathways from amygdala and hippocampus, learning is gated on and off by a sampling signal that grows with amygdala or hippocampal activity, plus BDNF activity (see Eqs. 10 and 11). When the sampling signal is on, it determines the rate at which the corresponding adaptive weight time-averages activity *O*_1_, thereby combining both Hebbian and anti-Hebbian learning properties.

##### Orbitofrontal BDNF

Orbitofrontal BDNF *B*_*Oi*_ (see Eq. 12) slowly time-averages the level of hippocampal activity *H*, and thereby extends its duration. Hereby this BDNF process helps to maintain cortical activity across an extended CS-US temporal gap during trace conditioning, and thus to support the consolidation of cortico-cortical category learning.

##### Habituative transmitter gates

As described above, the habituative transmitter gate at each cortical cell prevents unlimited perseverative activation of orbitofrontal cortical cells via their positive feedback loops. As before, such a habituative transmitter gate accumulates at a constant rate up to a maximum value, and is inactivated at a rate proportional to the size of the excitatory signal that it gates, multiplied by the amount of available transmitter (see Eq. 5).

#### Amygdala and conditioned reinforcer learning

##### Amygdala drive representation dynamics

The amygdala has a complex cytotonic architecture that represents emotional states and generates incentive motivational signals (Aggleton & Saunders, 2000). The amygdala is simplified in nSTART to enable conditioned reinforcer learning and incentive motivation learning to occur, as in the CogEM and START models (see Fig. 6). In the nSTART model, a single drive representation of amygdala activity *A* (see Eq. 14) is activated by the sum of excitatory inputs from sensory cortex *S*_*i*_ that are gated by conditioned reinforcer adaptive weights.

##### Conditioned reinforcer learning

These adaptive weights determine how well sensory cortex can activate *A*. Conditioned reinforcer learning is a key step in converting a conditioned stimulus into a conditioned reinforcer that can activate the amygdala. Together with incentive motivational learning in the pathway from the amygdala to the orbitofrontal cortex, a sensory cortical input can stimulate the amygdala which, in turn, can provide motivational support to fire orbitofrontal cortical cells (Fig. 2).

The CS cannot strongly excite the drive representation activity *A* before conditioning takes place. During conditioning, the US can directly activate *A* via its sensory representation. Pairing of CS-activated signals from the sensory cortex to the amgydala with those of the US to the amygdala causes conditioned reinforcer learning in the adaptive weights within the sensory cortex-to-amygdala pathways.

As in the case of incentive motivational learning, the learning law that is used for conditioned reinforcer learning is an *outstar learning* law (see Eq. 15) whereby a sensory cortical representation can sample and learn a spatial pattern of conditioned reinforcer adaptive weights across multiple drive representations. The current model simulations only consider such learning at a single drive representation.

#### Hippocampus and adaptive timing

##### Adaptively-timed hippocampal learning

As noted above, the hippocampus receives adaptively timed inputs that can maintain its activity for a duration that can span the trace interval. The hippocampus can hereby provide its own incentive motivational pathway to orbitofrontal cortical cells in cases when the amygdala cannot. In addition, BDNF at the model hippocampus and prefrontal cortex can sustain prefrontal cortical activity for an even longer duration. The adaptively timed “spectral timing” process spans several processing steps.

##### Adaptively-timed hippocampal activity

The adaptively timed signal *R* and the hippocampal BDNF signal *B*_*H*_ together maintain activity of the model hippocampus (see Eq. 16) across trace conditioning intervals, and also during periods after partial conditioning when no further external inputs are presented. In these latter periods, sustained hippocampal activity provides the incentive motivational signals that support memory consolidation of cortico-cortical category learning.

Figure 7f shows the functional relationships between hippocampal BDNF (*B*_*H*_), hippocampal activity (*H*), the hippocampal-to-orbitofrontal learned weight (*w*_*Hi*_), and the hippocampal-to-orbitofrontal stimulation of cortical BDNF (*B*_*Oi*_) production.

##### Adaptively-timed population output signal

The adaptively timed input from the sensory cortex to the hippocampus is the population output $R={\displaystyle \sum_{i,j}{h}_{ij}}$of spectrally-timed and learning-gated signals *h*_*ij*_ = 8*f*(*x*_*ij*_)*y*_*ij*_ *z*_*ij*_ (see Eq. 17). The individual signals *h*_*ij*_ are not well timed, but the population response *R* is, and its activity peaks around the *ISI*. Adaptively timed learning is thus an emergent property of this entire population of cell sites.

##### Activation spectrum

The components of the adaptively timed signal *R* are defined as follows: First, a population of hippocampal cell sites with activities *x*_*ij*_ (see Eq. 20) reacts to the excitatory input signal from sensory cortex at a spectrum of rates, ranging from fast to slow, that span the different ISIs to be learned. Activity *x*_*ij*_ generates a sigmoidal output signal *f*(*x*_*ij*_) to the next processing stage.

##### Habituative transmitter spectrum

Each signal *f*(*x*_*ij*_) is gated by with a habituative transmitter gate *y*_*ij*_ (see Eq. 22) that is similar in structure and function to the habituative transmitter gates described above. The different rates at which each spectral activity *f*(*x*_*ij*_) responds causes the corresponding habituative transmitter *y*_*ij*_ to habituate at a different rate. Habituative transmitter *y*_*ij*_ multiplies, or gates, the corresponding signal *f*(*x*_*ij*_) to generate a net output signal *g*_*ij*_ (see Eq. 23).

##### Gated signal spectrum and time cells

Multiplication of the increasing *f*(*x*_*ij*_) with the decreasing *y*_*ij*_ generates a unimodal curve *g*_*ij*_ = *f*(*x*_*ij*_)*z*_*ij*_ through time. Each *g*_*ij*_ peaks at a different time, and curves that peak at later times have broader activation profiles through time (see Fig. 11c), thereby realizing a *Weber law* property. Predicted properties of these cell responses were reported in neurophysiological data about hippocampal *time cells* (MacDonald et al., 2011). The Spectral Timing model predicts how such time cells may be used both to bridge the long ISIs that occur during trace conditioning, and to learn adaptively timed output signals that match the timing of experienced ISIs during delay or trace conditioning. This learning is proposed to occur in the following way.

##### Spectral learning law

To generate the adaptively-timed response *R*, each signal *g*_*ij*_ is multiplied, or gated, by a long-term memory (LTM) trace *z*_*ij*_ (see Eq. 24). In addition, *g*_*ij*_ helps to control learning by *z*_*ij*_: When *g*_*ij*_ is positive, *z*_*ij*_ can approach the value of a Now Print learning signal *N* at a rate proportional to *g*_*ij*_. Each *z*_*ij*_ thus changes by an amount that reflects the degree to which the curves *g*_*ij*_ and *N*, which represent sensory and reinforcement values, respectively, are simultaneously large. If *g*_*ij*_ is large while *N* is large, then *z*_*ij*_ will increase. If *g*_*ij*_ is large while *N* is small, then *z*_*ij*_ will decrease. Thus, adaptively timed learning selectively amplifies those *z*_*ij*_ whose sampling signals *g*_*ij*_ are on when *N* is on. Since the *z*_*ij*_ represent adaptively timed learned traces that persist across trials, they are not reset to initial values between trials but rather are cumulative across trials.

Signal *N* is activated transiently by increments in amygdala activity, and is thus active at times when the amygdala receives either US or conditioned CS inputs. A direct excitatory output signal from amygdala (see Eq. 14) and an inhibitory signal from an amygdala-activated inhibitory interneuron *E* (Eq. 26) combine to compute *N* (Eq. 25); see also Fig. 7d. In response to larger inputs *A*, *N* increases in amplitude, but not significantly in duration. Thus, learning rate can change without undermining learned timing.

##### Doubly-gated signal spectrum

The adaptive weight *z*_*ij*_ gates the sampling signal *g*_*ij*_ to generate a twice-gated output signal *h*_*ij*_ = 8*f*(*x*_*ij*_)*y*_*ij*_ *z*_*ij*_ from each of the differently timed cell sites (Eq. 18); see also Fig. 11d. Comparison of *h*_*ij*_ with *g*_*ij*_ in Fig. 11d shows how the population response $R={\displaystyle \sum_{i,j}{h}_{ij}}$ learns to match the ISI.

##### Hippocampal BDNF

*R* causes production and release of hippocampal BDNF *B*_*H*_ (see Eq. 27). Sustained BDNF activity helps to maintain hippocampal activity even longer than *R* can, and thus its incentive motivational support to orbitofrontal cortex across the CS-US ISI intervals during trace conditioning and memory consolidation (Fig. 7e).

#### The pontine nuclei

##### Final common path for conditioned output

Projections from the amygdala and orbitofrontal cortex input to the pontine nuclei (Fig. 7g). Pontine activity *P* controls output signals that generate a CR (Kalmbach et. al., 2009; Siegel et al., 2012; Woodruff-Pak & Disterhoft, 2007; see Eq. 28).

## Results

### Summary of six key simulation measures

Using a single set of model parameters, except for a variable US intensity, the following measurements are used to simulate the experimental data. Where there is an intact or partial hippocampus in the simulation, the adaptively timed signal within the hippocampus, *R*, is used to illustrate how the hippocampus reflects CR-timed performance, as seen in many experimental data (Berger, 1984; Schmaltz & Theios, 1972; Smith, 1968; Thompson, 1988). Orbitofrontal cortical activity, *O*, is reported since it is involved in activating downstream conditioned motor outputs (Kalmbach et al. 2009a, 2009b; Siegel et al., 2012; Woodruff-Pak & Disterhoft, 2007); and is a critical site of long-term memory consolidation in the model (see Eq. 7). In addition, the activity of the pontine nuclei *P* (see Eq. 28) is reported in all cases because it serves as a common output path for CR (Kalmbach et al. 2009a, 2009b; Siegel, et al., 2012; Woodruff-Pak & Disterhoft, 2007). To understand how CR activity is generated in the pons, the activity profiles of the sensory cortex (*S*), amygdala (*A*), and hippocampus (*H*) are also reported.

### Simulation of normal trace conditioning

Figure 8a shows behavioral data for normal trace conditioning during rabbit nictitating membrane conditioning for multiple ISIs in response to different US levels (Smith, 1968). These data exhibit the Weber law property whereby smaller ISIs generate earlier response peaks with narrower variances. The data also generally show the typical inverted-U envelope through time at each US intensity level for each ISI curve, as well as collectively for different ISI values. Finally, the data show that, whereas conditioned response timing is only sensitive to the ISI, response amplitude is also sensitive to US intensity (1, 2, and 4 MA).

Under the learning conditions in the Smith (1968) experiments, where a living animal has much more complex knowledge, motivation, and attentional distractions than in a computational model like nSTART, 110 trials, on each of 10 consecutive days, were completed to obtain the given CR data, which are smoothed averages of the individual trials. Smith noted that his data of “average topographies present a somewhat distorted picture of individual CRs…the later peak of the averaged response appeared to be later than the mean of the individual responses” (Smith, 1968, p.683; see Fig. 8a).

Figure 8b shows how hippocampal adaptive timing *R* in nSTART simulates these properties of normal conditioning on a recall trial, in response to the CS alone, after 20 prior learning trials for each ISI in response to three different US amplitudes. The peak activities and timing of both the cortex and the pontine nuclei (Fig. 8d) reflect the properties of the adaptively timed hippocampal output to them.

When orbitofrontal BDNF *B*_*O*1_ is eliminated after acquisition trials in model simulations, adaptive timing is impacted more negatively for longer ISIs (Fig. 8c). This learning impairment is due to a weakened cortico-cortico-hippocampal feedback loop, which is critical in trace conditioning.

nSTART is robust in that, with a single set of parameters, it can learn long ISIs better under normal conditions with additional learning trials; for example, the retention test output for ISI = 1,000 after 20 and 40 acquisition trials shows that peak *R* amplitude and timing changed from 0.5616 at 911 ms to 0.5393 at 949 ms, respectively. The activity profiles of the pontine nuclei are consistent with these results: *P* peak amplitude and timing changed from 1.311 at 639 ms, at 20 trials, to 1.689 at 601 ms, at 40 trials. These peak timings are within the effective 400-ms signaling window that has been found experimentally (Kalmbach et al. 2009a, 2009b; Siegel, et al., 2012; Woodruff-Pak & Disterhoft, 2007).

### Delay conditioning with and without hippocampus

A comparison of simulations of delay conditioning after five training trials with and without hippocampal lesions (see *H* in Fig. 9) and indicates that an intact model hippocampus is not required for delay conditioning (see *P* in Fig. 9a), as also occurs typically in the data (see Table 1). The involvement of the amygdala in each case (normal, 50 % partial ablation, and 80 % partial ablation) is apparent when their peak activities are compared. While *in vivo* the cerebellum typically is able to learn delay conditioning without forebrain processing, the model illustrates how the amygdala may motivationally support a parallel input channel to the pontine activity found in normal delay conditioning.

**Table 1:** The specific impact to learning and memory of the conditioned response by lesions of the hippocampus, cortex, amygdala, and thalamus is related to the phase of conditioning in which the lesions occur. Representative studies on rats, rabbits, and humans used various experimental preparations and performance criteria yet show patterns of effects on the acquisition and retention of a conditioned response (CR) for delay and trace paradigms based on the age of the memory (degree of consolidation)

Lesions of the hippocampus	Before conditioning	Early after conditioning	Late after conditioning
Delay paradigm	*CR acquisition: YES* Berger 1984 Chen et al. 1995 Daum et al. 1996 Ivkovich & Thompson 1997 Lee & Kim 2004 Port et al. 1986 Schmaltz & Theios 1972 Shors et al. 1992 Solomon & Moore 1975 Weizenkratz & Warrington 1979	*CR retention: YES* Akase et al. 1989 Orr & Berger 1985 Port et al. 1986 *CR retention: NO* *(long ISI)* Beylin et al. 2001	*CR retention: YES* Akase et al. 1989
Trace paradigm	*CR acquisition: NO* Anagnostaras et al. 1999 Berry & Thompson 1979 Clark & Squire 1998 Garrud et al. 1984 Gabrieli et al. 1995 Ivkovich & Thompson 1997 James et al. 1987 Kaneko & Thompson 1997 Kim et al. 1995 Little et al. 1984 McGlinchey-Berroth et al. 1997 Orr & Berger 1985 Flores & Disterhoft 2009 Schmajuk et al. 1994 Schmaltz & Theios 1972 Solomon & Moore 1975 Solomon et al. 1990 Weiss & Thompson 1991a&b Woodruff-Pak 2001	*CR retention: NO* Kim et al. 1995 Moyer et al. 1990 Takehara et al. 2003 *CR retention: YES* *(short ISI)* Walker & Steinmetz 2008	*CR retention: YES* Kim et al. 1995 Takehara et al. 2003
Lesions of the cortex	Before conditioning	Early after conditioning	Late after conditioning
Delay paradigm	*CR acquisition: YES* Mauk & Thompson 1987 McLaughlin et al. 2002 Oakley & Russell 1972 Takehara et al. 2003 Yeo et al. 1984	*CR retention: YES* Oakley & Steele Russell 1972 Takehara et al. 2003 Yeo et al. 1984	*CR retention: YES* Oakley & Steele Russell 1972 Takehara et al. 2003 Yeo et al. 1984
Trace paradigm	*CR acquisition: YES* Frankland & Bobtempi 2005 McLaughlin et al. 2002 *(short ISI)* Oakley & Steele Russell 1972 Simon et al. 2005 Takehara et al. 2003 Yeo et al. 1984 *CR acquisition: impaired* Kronforst & Disterhoft 1998 McLaughlin et al. 2002 *(long ISI)* Weible et al. 2000	*CR retention: YES* Frankland & Bobtempi 2005 Oakley & Steele Russell 1972 Simon et al. 2005 Takehara et al. 2003 Yeo et al. 1984	*CR retention: NO* Frankland & Bobtempi 2005 Oakley & Steele Russell 1972 Powell et al. 2001 Simon et al. 2005 Takehara et al. 2003 Yeo et al. 1984
Lesions of the amygdala	Before conditioning	Early after conditioning	Late after conditioning
Delay paradigm	*CR acquisition: YES but decelerated* Bechara et al. 1995 Blankenship et al 2005 Lee & Kim 2004	*CR retention: YES but impaired* Lee & Kim 2004 McGaugh 2002	*CR retention: YES* Lee & Kim 2004
Trace paradigm	--Data not found *Predict CR acquisition: YES but decelerated*	--Data not found *Predict CR retention*: *YES* Büchel et al. 1999 Chau & Galvez 2012	--Data not found *Predict CR retention*: *YES* Büchel et al. 1999 Chau & Galvez 2012
Lesions of the thalamus	Before conditioning	Early after conditioning	Late after conditioning
Delay paradigm	*CR acquisition: YES but decelerated* Buchanan & Thompson 1990 Halverson & Freeman 2006	--Data not found *Predict CR retention- Yes, but impaired*	--Data not found *Predict CR retention- Yes, but impaired*
Trace paradigm	*CR acquisition: YES but decelerated* Halverson, Poremba, & Freeman 2008 Powell & Churchwell 2002	--Data not found *Predict CR retention- Yes, but impaired*	--Data not found *Predict CR retention- Yes, but impaired*

This effect is enhanced after ten training trials (Fig. 9b). *In vivo*, output pathways like the pontine pathway are supplemented by adaptively timed cerebellar response learning, which would strengthen these tendencies.

Experimental data when the ISI is relatively long, for example 1,500 ms in rats, do show deficits in the initial timing and amplitude of the CR, and in the time to acquire the CR, when the hippocampus is damaged. These experimenters (Beylin et al., 2001) counted any response within 500 ms of US onset as a CR. We do not simulate this finding due to the variability of these results. They can, however, be qualitatively explained if the sensory cortical responses habituate at later times when the CS is sustained for such long durations. Then an at least partial temporal gap would be created between internal CS activations and US onset. This kind of result could then be explained using the same mechanisms that are used to explain deficits during trace conditioning after hippocampal damage.

### Delay and trace conditioning with and without amygdala

Simulations of amygdala lesions are also consistent with experimental data (graphs labeled *A* in Fig. 10). Delay conditioning with partial and complete amygdala lesions demonstrate the experimental finding (Lee & Kim, 2004) that the amygdala is required for optimal acquisition and retention of the CR, as reflected in the simulated hippocampal response amplitude for adaptive timing (*R*)*,* the orbitofrontal cortical response amplitude (*O*), and especially the pontine response amplitude (*P*). To simulate partial lesions of the amygdala in delay conditioning, the gain of the excitatory inputs from the sensory cortex to the amygdala (Eq. 14, parameter *β*_*A*_) is lowered from the baseline value of 40 to 30, and then to 20. When the growth rate is thus attenuated, there is normal timing in delay conditioning but with a smaller peak amplitude in the amygdala, and also in the hippocampus, which depends upon amygdala-triggered Now Print signals to train the temporal distribution of spectrally timed hippocampal learning (Fig. 10a). The lower peak amplitude reflects the fact that *in vivo* there is slower and weaker learning of the adaptively timed response. The experimental finding that 4–5 more days of training rats with amygdala lesions can support learning of the CR (Lee & Kim, 2004) may also include support from extra-amygdala circuits. Additional training also improves learning in the model (Fig. 10b). However, when the amygdala is completely ablated before training, there is no hippocampal response. The cortical and pontine peak amplitudes show similar results.

The dynamics of the nSTART cortico-cortico-hippocampal loop explains how aversive conditioning can occur with partial amygdala lesions. Activity in the model orbitofrontal cortex, based in part on hippocampal and amygdala inputs (Eq. 7), continues to support adaptively timed learning via its input to sensory cortex (Eq. 2), and sensory cortical input to the hippocampal activation spectrum (Eq. 19) supports adaptively timed learning (Eq. 17). For this to occur, there has to be enough amygdala input to generate a Now Print signal that shapes the adaptively timed response through learning. *In vivo*, other circuits are also involved that are outside the scope of the nSTART model (see Fig. 2), such as cerebellum, hypothalamus, and basal ganglia, but their responses are not rate-limiting in simulating the main effects above.

The amygdala is required for delay conditioning acquisition, but not for its expression. The cortico-cortico-cerebellar circuit can execute the timed response after learning. Simulations of complete amygdala lesions (outputs of Eq. 14 for amygdala and Eq. 15 for conditioned reinforcement are both zero) show that there is no CR learned if the lesion is made pre-training, but an acquired CR is retained if the lesion is made post-training (Fig. 10d), in agreement with some experimental data (Lee & Kim, 2004; Sosina, 1992) but not all (McGaugh, 2002; Siegel, et al., 2015). Furthermore, while Büchel et al. (1999) had reported decelerated trace conditioning when amygdala lesions were made before training, simulation of a 50 % partial lesion of the amygdala before trace conditioning followed by a retention test after 60 training trials (US onset at 750 ms, US level = 1) still shows severe impairments compared with 20 training trials. Perhaps the lesion is so large that recovery may not be possible at all (Siegel, et al., 2015).

In particular, the amygdala has been found to be unnecessary for fear conditioning acquisition in Pavlovian experimental paradigms in which the aversive US is so negative that autonomic reflex pathways may control the learning (Lehman et al., 2000; Vazdarjanova & McGaugh, 1998). However, in appetitive learning and instrumental conditioning, the amygdala is always required for acquisition (Cahill & McGaugh, 1990; McGaugh, 2002). This latter property is explained by the model hypothesis that conditioned reinforcer learning and incentive motivational learning both involve the amygdala, and provide positive attentional feedback that supports the rapid category learning required to enable the CS to elicit a CR via the orbitofrontal cortex (Fig. 2). Within the dynamics of the nSTART model, this kind of amygdala-mediated motivated attention supports the acquisition of delay and trace conditioning by strengthening adaptively timed attentional shifts based on learned cues. After conditioning, both delay and trace CRs may be mediated more completely by fast cortico-cortical activation of recognition categories via learned cortical weights that serve to activate the adaptively-timed cerebellar motor response without continued need for involvement of the amygdala or the hippocampus.

The nSTART model predicts that, if both amygdala and hippocampus are ablated before or after delay conditioning, then the amygdala lesion most influences delay conditioning, as above. If both amygdala and hippocampus are ablated before trace conditioning, then the model proposes how the hippocampal damage prevents the CR from being learned, because the required cortico-cortical connections that establish long-term memory trace could not be formed using spectral timing as a temporal bridge. Finally, if both amygdala and hippocampus are ablated long enough after trace conditioning ends, then the model predicts that strong learned cortico-cortical associations will already have formed.

Such cortico-cortical learning, supported by amygdala and hippocampus, is a primary form of memory consolidation in the model, but this form of consolidation does *not* imply that the “same information” is transferred from associative links that involve amygdala and hippocampus to cortico-cortical associations. In addition, the mechanism for memory consolidation that is simulated by nSTART does not propose that memory engrams are quickly learned by the hippocampus and then slowly transferred to the neocortex, as some have proposed, a proposal that seems beset with fundamental difficulties. Rather, nSTART demonstrates how hippocampal endogenous activation capable of bridging the temporal gap can energize the strengthening and consolidation of cortico-cortical pathways that are the same pathways that were partially learned before consolidation begins.

For simplicity, the nSTART model lumps amygdala and hypothalamus together, and thus does not simulate how spared hypothalamic connections might enable responding after an amygdala lesion. The MOTIVATOR model (Fig. 4c; Dranias, Grossberg, & Bullock, 2008; Grossberg, Bullock, & Dranias, 2008) explicitly simulates hypothalamic, amygdala, and basal ganglia contributions to conditioning and motivated performance that are consistent with the current results, and that can be incorporated without undermining the current results in a future extended model.

### Trace conditioning with and without hippocampus

Data from early, intermediate, and late stages of normal trace conditioning trace acquisition trials (McEchron & Disterhoft, 1997; Kim et al., 1995; Takehara et al., 2003) were simulated. In the nSTART model, learning to adaptively time a response to a stimulus is the result of an adaptively timed spectrum of cells. Figure 11a–e show the spectral activity and output during the simulation after the initial acquisition trial. This process unfolds as follows (see Fig. 7 for diagrams of network processing steps and Fig. 18 below for a complete circuit diagram).

As described in above, the signals *f*(*x*_*ij*_) are generated by the activities *x*_*ij*_(*t*) of the j^th^ spectral cell (or cell population) (*i,j*) in response to the *i*th input I_*i*_ (Eqs. 19–21, and Fig. 11a). Each *x*_*ij*_ responds at a different rate *r*_*j*_ to *I*_*i*_. In particular, we use *i* = 1 to represent the CS and *i* = 0 to represent the US. Thus, *f*(*x*_*1j*_) signals are generated by the CS. They cause the release of chemical transmitters *y*_1*j*_(*t*) that habituate, or are inactivated, at a rate proportional to their driving signals *f*(*x*_*1j*_) (Eq. 19, and Fig. 11b). The transmitters interact with, or gate, their respective signals to generate gated sampling signals *g*_*1j*_ that are products of *f*(*x*_*1j*_) and *y*_*1j*_ (Fig. 11c). These sampling signals *g*_*1j*_ are the differently timed responses of cell sites that together form the basis for spectrally timed learning.

Learning of the association between CS and US occurs at each spectral cell site only when its *g*_*1j*_ is positive. Thus, each *g*_*1j*_ samples learning of US activity that is correlated with it. Both the timing and rate of learning by the adaptive timing weights *z*_*1j*_ (Eq. 24) covary with the size of the corresponding *g*_*1j*_. Due to the fact that the various *g*_*1j*_ have their peak activities at different times, each site is maximally sensitive to learning correlations with different delays between CS and US.

The signals *g*_*1j*_ give rise to adaptively timed outputs *h*_*ij*_ = 8*g*_*ij*_ *z*_*ij*_ wherein the signals *g*_*1j*_ are multiplied, or gated, by their adaptive weights *z*_*1j*_ (Fig. 11d). When the adaptively weighted signals for all spectral components are added together, they form a total population output *R* that is adaptively timed to peak at, or near, the expected time of US onset. Thus, spectral timing is a property of an entire population of pathways that respond at different rates, not one of which, by itself, adequately represents accurate ISI timing. The hippocampal response after the initial acquisition trial is shown in Fig. 11e. Figure 11f shows data of McEchron and Disterhoft (1997) that exhibits similar timing from early acquisition trials. Figure 11g shows simulation output from the retention test after 20 acquisition trials; cf., Fig. 8.

The simulation of the property that trace conditioning depends on an intact hippocampus is shown in Fig. 12. The model proposes how a neurotrophic cascade from hippocampus to cortex supports learning of an associative connection between sensory cortex and orbitofrontal cortex in response to CS and US pairing during trace conditioning (Eq. 9). Unless there is enough time to build the cortico-cortical synaptic connections required to consolidate memory, both the timing and amplitude of learning rapidly degrade, as in anterograde amnesia.

Figure 12a summarizes simulations of how various levels of hippocampal ablation (normal: solid line; 50 % ablation: dashed line; 80 % ablation: dotted line) cause progressively weaker responses that also become premature after sufficient ablation. These effects are due to the elimination of many, but not all, of the adaptively timed hippocampal cell responses that, taken together, span the ISI, as shown in Figs. 11a–e. The duration of this spectral activity is also a key to understanding the role of the hippocampus in trace conditioning and consciousness. Even in the case of an 80 % lesion, Fig. 12b shows that extended training yields some improvement in the timing and amplitude of response indicators for adaptive timing within the hippocampus (*R*) and the pontine nuclei (*P*).

The nSTART prediction of when and how the hippocampus is involved in cortical learning was described above and is illustrated by the simulation results in Fig. 13. Figure 13a simulates the property that the establishment of a long-term memory as a result of trace conditioning requires a critical consolidation period with a normally functioning hippocampus. Figure 13a (first row) compares effects of early hippocampal ablation with delayed hippocampal ablation on orbitofrontal peak amplitude, which provides one measure of the strength of the CR. In the partially trained case with five acquisition trials (first row, left column), a reduction in cortical activity results if the hippocampal ablation is made early (dotted line), immediately after acquisition and before the consolidation period, during which there are no stimulus (NS) trials before the CS, as compared with the activity that is attained after a late ablation (solid line), which is made after the NS trials and just before CS. In contrast, in the fully trained case after 20 acquisition trials (first row, right column), no impairment ensues. There is no difference in orbitofrontal activity between early hippocampal ablation (dotted line) and late hippocampal ablation (solid line) because cortico-cortical connections have already become sufficiently large before the ablation occurs. These simulations are in agreement with experimental data (Kim et al., 1995; McEchron & Disterhoft, 1997; Moyer et al., 1990; Takehara et al., 2003).

The adaptive weights from sensory cortex to orbitofrontal cortex for each of the cases in Fig. 13a (first row) are shown in Fig. 13a (second row). In particular, the lower two graphs show cortico-cortical adaptive weights that covary with the orbitofrontal cortical activity for each scenario. After partial training with five acquisition trials, early hippocampal ablation prevents an increase in adaptive weight because a critical source of incentive motivational support from the hippocampus is removed before the weight can reach an asymptote (Fig. 13a, second row, left column, dotted line). Late hippocampal ablation (Fig. 13a, left column, solid line) enables weight learning to benefit from this support. After 20 trials of training to asymptote, hippocampal support is no longer needed (Fig. 13a, second row, right column).

It should, however, be emphasized that activation of sensory cortex will continue to activate both the orbitofrontal cortex and hippocampus after learning is complete. This kind of memory consolidation does not imply that the “memory trace” moves from hippocampus to orbitofrontal cortex (cf., Nadel & Moscovitch, 1997).

When hippocampal BDNF is eliminated after acquisition trials (Fig. 13b), the simulation results are largely unchanged. However, when both hippocampal and orbitofrontal BDNF are removed after acquisition trials in the partially trained case (Fig. 13c, left column), there are the same deleterious effects on orbitofrontal activity (Fig. 13c, left column, first row) and on cortico-cortical weights (Fig. 13c, left column, second row) for both the early and late ablation treatments, due to the lack of orbitofrontal BDNF support for consolidation. In the fully trained case (Fig. 13c, right column), removal of hippocampal and orbitofrontal BDNF during early and late ablation treatments yield similar orbitofrontal activities (Fig. 13c, right column, first row) and cortico-cortical weights (Fig. 13c, right column, second row) because consolidation has already occurred. Measures of pontine activity in the model also support this analysis since they are driven by cortical input.

### Delay and trace conditioning with and without thalamus or sensory cortex

Thalamic lesions negatively affect many types of learning since the thalamus is the gateway to perception and higher-levels of emotional and cognitive processing. Experimental data on thalamic lesions before delay or trace conditioning slow acquisition to some degree (Buchman & Thompson, 1990; Powell & Churchwell, 2002). However, the deficit is greater in trace conditioning than in delay conditioning, since there are then alternate paths available for auditory CS representations to the cerebellum.

The model predicts that lesions to the thalamus, with an equivalent effect on sensory cortex, that are made after delay or trace conditioning would also impair retention for two reasons: (1) disruption of stimulus input processing, and (2) damage to the pathways that support cortico-cortical learning of the association between CS and US, which also serve to control CR performance in the post-consolidation stage of learning. Figure 14 shows that general CR acquisition is impaired in proportion to the extent of the lesion, as reflected in the simulated hippocampal response amplitude (*R*)*,* orbitofrontal cortex (*O*), and pontine nuclei (*P*). The simulations show that, as *in vivo* for thalamic lesions, the disruption to trace conditioning (Fig. 14b) is more severe than disruption to delay conditioning (Fig. 14a). Extended training (doubling the number of training trials) improves performance for delay conditioning (Fig. 14c) but causes little improvement for trace conditioning in the lesion cases, although it does cause improvement in the no lesion case (Fig. 14d).

#### Conditioning, consciousness, and amnesia

The link between consciousness and conditioning (Clark, Manns, & Squire, 2002) is clarified by contrasting what happens during delay versus trace conditioning in normal and amnesic subjects. The nSTART model requires a sustained interaction of sensory cortex, orbitofrontal cortex, and hippocampus to achieve trace conditioning. From his clinical data from brain-damaged patients, Damasio (1999, pp. 157–158, 195ff, 265) heuristically derived a CogEM-type model and noted that conscious awareness of “the feeling of what happens” relies on a sustained feedback interaction. The nSTART model (Fig. 2) builds on the START model (Grossberg and Merrill, 1992, 1996) to explain this sort of data with its prediction that this sort of conscious awareness is supported by a sustained, adaptively timed, cognitive-emotional resonance, which is mechanized as a temporal-amygdala-orbitofrontal resonance that is supported by hippocampal feedback. This specific resonance specializes the ART prediction that “all conscious states are resonant states” (Grossberg, 1999). This explanation clarifies why trace conditioning is facilitated by conscious awareness but delay conditioning is not, why a normal subject may not be consciously aware of delay conditioning, and why amnesics with bilateral hippocampal lesions perform like unaware controls on delay and trace conditioning.

In particular, the emotional path via amygdala operates more quickly than the cognitive path of self-awareness via hippocampus. Furthermore, during delay conditioning, adaptively-timed responding can be controlled through the cerebellum, so the hippocampus is not a critical component of successful delay conditioning and, thus, neither is awareness.

Recent experiments have supported the CogEM prediction (Grossberg, 1975, 1984) that emotional responses are part of an attentive cognitive-emotional resonance, and that amygdala activity may be influenced by factors such as stimulus valence, attentional load, competing cognitive task demands, and ambiguity (Pessoa, Padmala, & Morland, 2005; Pessoa, Japee, & Ungerleider, 2000). These experimental results are, moreover, consistent with the hypothesis that a sustained cortico-cortico-hippocampal resonance supports consciousness, since parallel hippocampal and amygdala activations occur during normal conditioning. Indeed, adaptively-timed hippocampally timed cognitive-emotional resonances are predicted to help prevent premature reset by the attentional focus on a valued goal object expected disconfirmations by task-irrelevant cues (Grossberg & Merrill, 1992, 1996). A hippocampal role is also consistent with the facts that lesions to the amygdala slow acquisition of delay conditioning, but do not impact already acquired responses (Lee & Kim, 2004) and that, although amygdala plays a key role in associative learning, researchers also note that: “circuitry within the amygdala (AM) or a closely related structure is necessary for some aspects of the formation, maintenance, or expression of these CRs” (Choi & Brown, 2003, p. 8713).

### Anterograde and retrograde amnesia

The model clarifies data related to the production of retrograde amnesia due to ablation of the medial prefrontal cortex before, during, or after completion of the consolidation process. Whereas the hippocampus is necessary for the acquisition and consolidation of trace conditioning – the lack thereof causes anterograde amnesia and recent retrograde amnesia (Clark, Broadbent, Zola, & Squire, 2002; Clark & Squire, 1998; Gabrieli et al., 1995; McGlinchey-Berroth et al., 1997; but see also Bayley, Frascino, & Squire, 2005) – the medial prefrontal cortex is necessary for the retention of a high percentage of CRs after trace conditioning occurs in normal subjects. In agreement with data (Kronforst-Collins & Disterhoft, 1998), the simulated CR that results when the orbitofrontal cortex is ablated before or after 20 trace conditioning trials shows impaired timing and amplitude in the pontine nuclei responses (Fig. 15b and d, respectively). Takehara et al. (2003) analyzed this phenomenon as a failure to retain or retrieve memory of the associated adaptive response, and not a simple failure of adaptive timing, because the ablation in their experiments did not affect CR timing. In the nSTART model, the notion that the orbitofrontal cortex provides a critical pathway that helps to read-out the conditioned response via connections to the pontine nuclei is consistent with this retrieval interpretation. In addition, since direct damage to motor cortex does not impair trace eyeblink conditioning (Ivkovich & Thompson, 1997), an alternative interpretation that a motor circuit has failed is not supported.

In the nSTART model, orbitofrontal cortical ablation also interferes with the ability of the CS to sustain the learned cortico-cortical resonance that results in an adaptively timed response profile of the CR in the hippocampus. Indeed, anterograde amnesia may also result if new memories cannot be consolidated due to cortical insult that prevents, or greatly weakens, such a resonance (see Fig. 13c). Figure 15a and c show that, when the model orbitofrontal cortex is ablated before or after five delay conditioning trials, the CR is not negatively affected, which fits data showing that delay conditioning does not require conscious awareness of the stimulus contingencies (Clark & Squire, 1998; Manns, Clark & Squire, 2001) and that amnesics can learn delay conditioning, but not trace conditioning (Clark, et al., 2001).

The intact hippocampus may also support sustained conscious resonance during normal delay conditioning, but it is not required for the ISI durations in the cited studies: “…those conditioning tasks that require the integrity of the hippocampus are the same tasks that aware participants can acquire and unaware participants cannot…” (Clark & Squire, 2004, p. 1467). In particular, for these ISIs, there may not have been enough time to generate a fully developed conscious cognitive-emotional resonance.

These simulation results display the temporal properties of hippocampal and cortical involvement in normal learning involving declarative memory. Amnesia data properties, such as the loss of recent memory, the inability to form new memory, or the loss of remote memory, are consistent with these dynamics in terms of the age of the memory when processing becomes abnormal: with hippocampal injury, new memories rapidly perish while old memories persist; with cortical injury (Fig. 13), new memories might be formed with support from other structures, depending on what cortical structures were damaged, while old memories that critically depend on the cortex perish. Cortical injury may involve the lack of activity in ablated areas, or hyperactivity in the remaining functioning cells (Li, Bandrowski, & Prince, 2005). In any case, the magnitude of the learning deficit depends on locations and scope of damage. Specific effects of interruption on learning and memory – that is, the type of amnesia – are dependent on the task, the stage of learning, and the specific brain area that is deficient, among other variables. The current model illustrates how lesions of several different brain areas, at different times before, during, or after the course of learning, can differentially contribute to this complex pattern of behavioral deficits.

In summary, the nSTART model simulates and qualitatively explains key data patterns concerning how thalamic, prefrontal cortical, amygdala, and hippocampal lesions may influence learning and memory. These data patterns are summarized in Table 1, including, for example, the hallmark hippocampal activity profiles over time during delay conditioning (Berger et al., 1980) and trace conditioning (McEchron & Disterhoft, 1997), the role of hippocampal and cortical lesions in influencing acquisition and retention of recently learned versus remotely learned eyeblink responses (Kim et al., 1995; Takehara et al., 2003), and the ability of amnesic individuals to do delay conditioning, but not trace conditioning, along with corresponding differences in conscious awareness (Clark et al., 2001).

Additional data support the conclusion that the hippocampus is typically essential during acquisition of trace conditioning, while the neocortex is needed for normal retention. In particular, research in discriminative avoidance conditioning found that hippocampal control of thalamo-cortical excitatory volleys determined timing of CR output during acquisition; otherwise, signals from anterior ventral thalamic nuclei and feedback from cingulate cortex area 29 determined timing of CR output during maintenance of learning (Gabreil, Sparenborg, & Stolar, 1987). These data support the facts that, while recent Nictitating Membrane Response (NMR) learning involving the trace conditioning paradigm is severely impaired by hippocampal lesions, its acquisition is resistant to cortical lesions. Conversely, NMR trace conditioning retention is not impaired by hippocampal lesions, but it is impaired by cortical lesions (Frankland & Bontempi, 2005; Oakley & Steele Russell, 1972; Simon, Knuckley, Churchwell, & Powell, 2005; Takehara et al., 2003; Yeo, Hardiman, Moore, & Steele Russell, 1984). In cases where the ISI is relatively short, the hippocampus is not required to support acquisition of the CR (Beylin et al., 2001), corresponding to nSTART short-term memory circuits the persistent activities of which in both sensory cortical and amygdala representations are capable of bridging short temporal gaps.

The nSTART model proposes how the hippocampus consolidates learning of thalamo-cortical and cortico-cortical associations by using the same adaptively-timed pathways by which the hippocampus learns to adaptively time the appropriate duration of motivated attention in a task-selective manner (Grossberg & Merrill, 1992, 1996). By means of a consolidation process that is driven by BDNF-mediated endogenous hippocampal bursting, which *in vivo* is also driven by continual periodic septal input (Smythe et al., 1992), and BDNF modulation of local, activity-dependent circuits (Schuman, 1999; Thoenen, 1995; Tyler et al., 2002), these associations are stored and recalled in cortico-hippocampal, hippocampo-cortical and cortico-cortical pathways (Sakurai, 1990), as demonstrated through nSTART computer simulations of the corresponding model pathways and mechanisms.

The fact that amygdala is not required after consolidation of Pavlovian conditioning does not contradict the claim of the CogEM model that amygdala is required for reinforcement learning for CR acquisition and performance. The polyvalent constraint on CogEM during learning is not required for performance in the consolidated case of aversive conditioning because the cortico-cortical connection along with extra-amygdala circuits, such as those involving volitional signals from the basal ganglia, would be sufficient to support performance. Indeed, Chang, Grossberg, and Cao (2014) have shown how such a convergence between cortico-cortical and basal ganglia volitional signals can initiate a directed search for a desired goal object in a cluttered scene, thereby illustrating how the Where’s Waldo problem may be solved.

## Discussion

### Five different types of learning interact during conditioning and memory consolidation

The nSTART model proposes that at least five different types of learning typically occur in parallel to ensure that associations can be formed and consolidated across temporal gaps, as occurs during trace conditioning (Fig. 2). As described above, the nSTART model includes: *CS category learning* via thalamo-cortical and cortico-cortical circuits, *conditioned reinforcement learning* via thalamo-amygdala and sensory cortical-amygdala circuits, *incentive motivational learning* via amygdala-orbitofrontal cortical circuits, and *adaptively-timed learning of motivated attention* via sensory cortical-hippocampal-orbitofrontal cortical circuits. There is also *adaptively-timed learning of motor responses* via the cerebellum (Figure 16), but this is not simulated in the current study. The key brain structures and processes explicitly represented in the nSTART model are summarized in Table 2.

**Table 2:** nSTART: system equations, variables, and parameters

System equation	Variable	Value
(2) Sensory Cortical Dynamics	*S*_*i*_	Initial value = 0
	*β*_*S*_	25
	Conditioned Stimulus	*I*_*1*_ = 1
	Unconditioned Stimulus	*I*_*0*_ = 1, 2 or 4
	*f*_*S*_(*S*_*i*_)	See Equation 4
	*O*_*i*_	See Equation 7
	*S*_*mi*_	See Equation 6
(3) Thalamic Dynamics	*T*_*i*_	*S*_*i*_; See Equation 2
(4) Signal Functions in the Recurrent On-Center Off-Surround Network	*f*_*S*_(*S*_*i*_)	Initial value = 0
		max(*S*_*i*_ ─ 0.02)
(5) Habituative Transmitter Gates	*N*_*mi*_	Initial value = 1
		For sensory cortex (*S*_*mi*_), see Equation 6. For prefrontal cortex (*O*_*mi*_), see Equation 13.
(6) Habituative Transmitter Gates: Sensory Cortex	*S*_*mi*_	Initial value = 1
		See Equation 2
(7) Corticocortical Category Learning	*O*_*i*_	Initial value = 0
	*β*_*O*_	12.5
	*f*_*S*_(*S*_*i*_)	See Equation 4
	*w*_*Si*_, *w*_*Ai*_, *w*_*Hi*_	See Equation 5
	*A*	See Equation 14
	*H*	See Equation 16
	*B*_*Oi*_	See Equation 12
	*O*_*mi*_	See Equation 13
(8) Prefrontal Cortical Dynamics: Conditioned Weights at Cortical Synapse (*M* = *S* (sensory cortex), *A* (amygdala) and *H* (hippocampus))	*w*_*Mi*_	Initial values = 0.01 No inter-trial reset.
	*f*_*M*_(*M*)	If *M=S,* see Equation 9; if *M=A,* see Equation 10; if *M=H,* see Equation 11.
	*B*_*Oi*_	See Equation 12
	*O*_*i*_	See Equation 7
(9) Prefrontal Cortical Dynamics: Conditioned Weights at Cortical Synapse for sensory cortex)	*w*_*Si*_	Initial value = 0.01
	*f*_*S*_(*S*_*i*_)	See Equation 4
	*B*_*Oi*_	See Equation 12
	*O*_*i*_	See Equation 7
(10) Prefrontal Cortical Dynamics: Conditioned Weights at Cortical Synapse for Amygdala	*w*_*Ai*_	Initial value = 0.01
	*A*	See Equation 14
	*B*_*Oi*_	See Equation 12
	*O*_*i*_	See Equation 7
(11) Prefrontal Cortical Dynamics: Conditioned Weights at Cortical Synapse for Hippocampus	*w*_*Hi*_	Initial value = 0.01
	*H*	See Equation (16)
	*B*_*Oi*_	See Equation 12
	*O*_*i*_	See Equation 7
(12) Cortical BDNF	*B*_*Oi*_	Initial value = 0 No inter-trial reset.
	*H*	See Equation 16
	*w*_*Hi*_	See Equation 11
(13) Habituative Transmitter Gates: Prefrontal Cortex	*O*_*mi*_	Initial value = 1
		See Equation 7
(14) Amygdala Drive Representation Dynamics	*A*	Initial value = 0
	*β*_*A*_	40
	*f*_*S*_(*S*_*i*_)	See Equation 4
	*F*_*i*_	See Equation 15
(15) Conditioned Reinforcer Learning	*F*_*i*_	constant value *F*_*0*_ = 0.50, Initial value *F*_*1*_ = 0.05. No inter-trial reset.
	*f*_*S*_(*S*_*i*_)	See Equation 4
	*A*	See Equation 8
(16) Adaptively-Timed Hippocampal Activity	*H*	Initial value = 0
	*β*_*H*_	5
	*R*	See Equation 19
	*B*_*H*_	See Equation 27
(17) Adaptively-Timed Population Output Signal	*R*	
	*h*_*ij*_	See Equation 18
(18) Doubly Gated Signal Spectrum (timed responses)	*h*_*ij*_	Initial value = 0
	*f(x*_*ij*_ *)*	See Equation 19
	*y*_*ij*_	See Equation 22
	*z*_*ij*_	See Equation 24
(19) Sigmoidal Signal Processing	*f(x*_*ij*_ *)*	Initial value = 0
(20) Activation Spectrum	*x*_*ij*_	Initial value = 0
	*r*_*j*_	See Equation 21
	*f*_*S*_(*S*_*i*_)	See Equation 19
(21) Differential Rates of Spectral Timing	*r*_*j*_	Range from 0.016 to 0.171
	*j*	Vary from 1 to 20
(22) Habituative Transmitter Spectrum	*y*_*ij*_	Initial value = 1
	*f(x*_*ij*_ *)*	See Equation 19
(23) Gated Signal Spectrum	*g*_*ij*_	Initial value = 0
	*f(x*_*ij*_ *)*	See Equation 19
	*y*_*ij*_	See Equation 22
(24) Spectral Learning Law	*z*_*ij*_	Initial value = 0. No inter-trial reset.
	*g*_*ij*_	See Equation 23
	*N*	See Equation 25
(25) Now Print Signal	*N*	Initial value = 0
	*A*	See Equation 14
	*E*	See Equation 26
(26) Inhibitory Interneuron	*E*	Initial value = 0
(27) Hippocampal BDNF	*A*	See Equation 14
	*B*_*H*_	Initial value = 0. No inter-trial reset.
	*R*	See Equation 17
(28) Pontine Nuclei	*P*	Initial value = 0
	*A*	See Equation 14
	*O*_*1*_	See Equation 7

### Multiple hippocampal functions: Space, time, novelty, consolidation, and episodic learning

The nSTART model does not presume to summarize all the functional roles that are played by the hippocampus *in vivo*. The hippocampus is known to participate in multiple functions, including spatial navigation, adaptively-timed conditioning, novelty detection, and the consolidation of declarative (notably, episodic) learning and memory. The hippocampus hereby raises a general issue that is confronted whenever one tries to understand how a given brain region works: Why does each brain region support a particular combination of processes, rather than a different one? How do these processes interact in a way that makes functional sense of their anatomical propinquity? Related neural models have clarified how some of these other processes work, and why they are near one another anatomically. They are briefly reviewed in this section. The articles that develop these models include citations of many relevant experimental data.

In particular, these models indicate that more than one hippocampal process may be at work in parallel during memory consolidation. This expanded view of memory consolidation is clarified by model explanations of why novelty detection has been linked to the process of memory consolidation during the learning of recognition categories, whether or not this learning needs to bridge a long temporal gap. Adaptive Resonance Theory, or ART, proposes how a memory search can occur during the learning of recognition categories, and how a sufficiently big mismatch between learned top-down expectations and bottom-up feature patterns can activate the novelty-sensitive orienting system (Fig. 3), which includes the hippocampus, to drive a memory search for a better matching category. The size of such a mismatch registers how novel the current stimulus is when calibrated against active top-down expectations. ART explains how such memory searches lead to learning of a stable, or consolidated, recognition category that requires no further searches, and thus to the cessation of hippocampal novelty potentials (Figs. 3 and 17). After consolidation of a category is complete, presentation of a familiar object exemplar causes direct access to the globally best-matching category via thalamo-cortical and cortico-cortical pathways.

Carpenter and Grossberg (1993) and Grossberg (2013) have noted how these properties can qualitatively explain quite a few data about medial temporal amnesia when the model hippocampus is ablated, thereby eliminating memory search during the consolidation process. These properties include unlimited anterograde amnesia, limited retrograde amnesia, perseveration, difficulties in orienting to novel cues, a failure of recombinant context-sensitive processing, and differential learning by amnesics and normals on easy versus demanding categorization tasks.

Thus, in addition to the important role of *adaptively-timed* hippocampal responses in bridging temporal gaps when events to be associated are separated in time, the hippocampus is also part of the *novelty-sensitive memory search* system for consolidating thalamo-cortical and cortico-cortical category learning. Both of these processes are included in START model circuits (Fig. 6), but without the enhancements that have enabled nSTART to simulate challenging data about early versus late lesions of amygdala, hippocampus, and orbitofrontal cortex during delay and trace conditioning.

The adaptively*-*timed hippocampal circuits are part of a larger theory about why both spatial and temporal representations exist within the entorhinal-hippocampal system. Neural models have provided a unified explanation of how these spatial representations (Mhatre, Gorchetchnikov, & Grossberg, 2012; Grossberg & Pilly, 2012, 2014; Pilly & Grossberg, 2012, 2014) and temporal representations (Grossberg & Merrill, 1992, 1996; Grossberg & Schmajuk, 1989) may arise in the entorhinal-hippocampal system during development and adult learning, and how they interact with other brain regions to control navigational behaviors and episodic learning and memory. This explanation emphasizes the fundamental role of brain designs for learning, attention, and prediction, and along the way articulates a rigorous mechanistic sense in which the hippocampus is indeed a “cognitive map” (O’Keefe & Nadel, 1978). This learning perspective also leads to the prediction that the network laws that give rise to the apparently very different behavioral properties of space and time are controlled by mechanistically homologous brain mechanisms, thereby clarifying why these spatial and temporal representations both occur in the entorhinal-hippocampal system, and how they can thus more easily interact to control navigation and episodic memory.

The timing model in question is the Spectral Timing model that has been used to explain and simulate data about normal and abnormal delay and trace conditioning (Grossberg & Merrill, 1992, 1996; Grossberg & Schmajuk, 1989). Due to the computational homolog between spatial and temporal representations, the spatial model is called the Spectral Spacing model (Grossberg & Pilly, 2012, 2014). Both models learn to represent spatial and temporal properties of the environments that animals or humans experience (Gorchetchnikov & Grossberg, 2007).

In the case of the Spectral Spacing model, this learning leads to grid cell receptive fields of multiple spatial scales along the dorsoventral axis of the medial entorhinal cortex that cooperate to form hippocampal place cells that can represent large spaces. In the case of the Spectral Timing model, this learning enables “time cells” that response at multiple temporal scales to cooperate to represent large time intervals. As noted earlier, the Spectral Timing model predicted in the 1980s the properties of time cells that have been reported in the hippocampus during the past few years, notably their Weber law properties. In both the Spectral Spacing and Spectral Timing models, a spectrum of cell rates generates a spatial gradient of cells with different properties. In the case of the Spectral Spacing model, grid cells with increasing spatial scales are learned along the dorsoventral axis of the medial entorhinal cortex. In the case of the Spectral Timing model, time cells with increasing onset times and variances are generated. It has been shown how Spectral Timing can be achieved using properties of the metabotropic glutamate receptor (mGluR) system, which proposes a biochemical basis for the ability of these cells to span such long time intervals (Fiala, Grossberg, & Bullock, 1996). An open question is whether the Spectral Spacing model uses a similar mechanism, suitably specialized?

These homologous spatial and temporal mechanisms have been used to provide a unified theoretical explanation, and quantitative computer simulations, of a body of challenging behavioral and neurobiological data about both space and time that have no other unified explanation at this time, leading to the name *neural relativity* for this mechanistic homology. In particular, the current study proposes how at least some time cells may participate in memory consolidation that requires the ability of the hippocampus to bridge across temporal gaps between stimuli that are associated through conditioning.

The coexistence of spatial and temporal learning in the hippocampus may support its role in episodic learning and memory, since episodic memories typically combine both spatial and temporal information about particular autobiographical events (Eichenbaum & Lipton, 2008; Tulving, 1972). The nSTART model does not include spatial representations, or the prefrontal working memory and list chunking networks for temporary and long-term storage of sequential information, and thus does not attempt to explain data about episodic learning and memory. Activation of such spatially-dependent episodic memories may always require hippocampal spatial representations, so a restricted gradient of retrograde amnesia may not be expected after hippocampal lesions that eliminated them. As noted within the “multiple traces” proposal of how memory consolidation works (Nadel & Moscovitch, 1997, p. 222): “The most parsimonious account of the data would be to assume that the hippocampal complex and neocortex continue to be involved in both the storage and the retrieval of episodic memory traces throughout life.”

Episodic memories may depend upon knowledge of *sequences* of correlated object and spatial information, not just information about individual ones. This kind of sequential information is also important for carrying out context-sensitive searches for desired objects in scenes. For example, seeing a refrigerator and a stove at particular positions in a familiar kitchen may generate an expectation of seeing a sink at a different position. A large psychophysical database about contextual cueing (e.g., Brockmole et al., 2006; Chun, 2000; Chun & Jiang, 1998; Jiang & Wagner, 2004; Lleras & von Mühlenen, 2004; Olson & Chun, 2002) describes how both object and spatial information contribute to such expectations, while they drive efficient searches to discover and act upon desired goal objects. The ARTSCENE Search model (Huang & Grossberg, 2010) simulates how computation of spatial and object working memories, list chunks, and spatial and object priming signals may be accomplished using interactions between the perirhinal and parahippocampal cortices (Bar, Aminoff, & Schacter, 2008; Brown & Aggleton, 2001; Epstein, Parker, & Feiler, 2007; Murray & Richmond, 2001), prefrontal cortex, temporal cortex, and parietal cortex to simulate key psychophysical data from contextual cueing experiments. The nSTART, ARTSCENE Search, and Spectral Spacing models may in the future be fused to provide a foundation on which to build a more complete theory of episodic learning and memory.

### Alternative models of memory consolidation

The popular *unitary trace transfer hypothesis* assumes that there is a memory representation that is first stored in the hippocampus and then transferred to the neocortex to be consolidated (McClelland, McNaughton, & O’Reilly, 1995; Squire & Alverez, 1995). McClelland et al. (1995) thus propose “a separate learning system in the hippocampus and why knowledge originally stored in this system is incorporated in the neocortex only gradually” (p. 433). This hypothesis is justified by the assumption that the hippocampus can learn quickly, but the neocortex can only learn slowly, so the hippocampus is needed to first capture the memory and then that same memory representation is transferred to the more slowly learning neocortex. There are, however, fundamental conceptual and mechanistic problems with a unitary trace transfer hypothesis as presented by McClelland et al. (1995) that persist in more recent expositions (Atallah, Frank, & O’Reilly, 2004; O’Reilly & Rudy, 2000): a *representation* problem, a *learning rate* problem, and a *real-time learning* problem. These problems are illustrated by considering how the unitary trace hypothesis might explain how a normal person can see a movie once and remember it well enough to describe it later to a friend in considerable detail, even though the scenes flash by quickly.

The *representation problem* concerns the implicit claim that the hippocampus can represent and store all the remembered visual and auditory memories in the movie. There seems to be no experimental evidence, however, that the hippocampus contains such specialized perceptual representations. Moreover, if the hippocampus did contain all the perceptual representations that were needed to represent all visual and auditory memories, then what does the specialized perceptual circuitry of visual and auditory neocortex do? In this regard, the unitary trace modelers never simulate the perceptual contents of the memories that are assumed to be stored in hippocampus and transferred to neocortex.

The *learning rate problem* concerns the factual basis for the claim that the neocortex must learn slowly. In fact, there are numerous examples that fast perceptual and recognition learning can occur in the neocortex (e.g., Fahle, Edelman, & Poggio, 1995; Kraljic & Samuel, (2006); Sireteanu & Rettenbach, 1995, Stanley & Rubin, 2005; Wagman, Shockley, Reley, & Tervey, 2001). In addition, no evidence is presented by unitary trace transfer theorists that there are slower learning synapses in neocortex than hippocampus. Even one of the proponents of the slow cortical learning hypothesis has equivocated on this point: “data that appear to support the limited cortical learning view tend to be based on larger lesions of the medial temporal lobe…it is becoming clear that the cortex is capable of quite substantial learning on its own…” (O’Reilly & Rudy, 2000, p.395).

The *real-time learning problem* is admitted by the modelers but not solved. A model that has been used in unitary trace model simulations is back propagation. It is well-known that this model is not biologically plausible (e.g., Grossberg, 1988, Section 17). Back propagation must carry out *slow learning*. Its adaptive weights can change only slightly on each learning trial, thus requiring large numbers of acquisition trials to learn every item in its memory. If the learning rate is sped up, then the model can experience catastrophic forgetting. It is incapable of the kind of fast learning that is experienced while watching a movie or other rare but motivationally engaging series of events. It can only carry out supervised learning, which means that an explicit teacher provides external feedback about the correct response on every learning trial, unlike the *unsupervised learning* that is characteristic of many biological learning experiences, including watching a movie. Its learned weights are computed using an unrealistic *non-local weight transport m*echanism that has no analog in the brain. Finally, because of its slow learning requirement, it is important that the data that are being learned have *stationary statistical properties*, so that each weight gets enough exposure to these properties over many learning trials to enable enough weight growth to occur. In other words, the probabilities of sequential events do not change through time, unlike the world in which we live.

In order to manage these weaknesses of back propagation, McClelland et al. (1995) developed their model based on a process of *interleaved learning* which is said to occur when memories are slowly transferred from the hippocampus to the neocortex via incremental adjustments in the neocortical representations, while being supervised by hippocampal teaching signals. Various sets of parameter values were used to fit their model to each of four data sets with varying degrees of success. Nevertheless, the authors state that such “…interleaved learning systems… are not at all appropriate for the rapid acquisition of arbitrary associations between inputs and responses” (McClelland et al., 1995, p. 432); in other words, their proposed model cannot do learning in real time.

Similar explanatory limitations are faced by connectionist models such as the one proposed by Moustafa, et al. (2013) that does not simulate biophysical properties of neurons, does not use a model that describes the anatomical areas involved in delay and trace conditioning, and does not consider the consolidation process. In addition, this model assumes a non-existent direct connection from hippocampus to motor output.

Beyond the self-criticism offered by MeClelland et al. (1995), the unitary trace view of memory consolidation has come under criticism from various researchers on both theoretical and experimental grounds. McGaugh (2000) points to protein synthesis and various neurotransmitters as providers of endogenous modulation of consolidation. In his view, the supposition that the molecular and cellular machinery of consolidation memory works slowly is “clearly wrong” (p. 248). Rather, consolidation seems slow because on-going experience *modulates* memory strength. In McGaugh's view, the amygdala plays a central role in modulating memories and, thus, in memory consolidation. Lesions of the amygdala disrupt the influence of epinephrine and glucocorticoids from the adrenal gland and, therefore, the consolidation process. In this view, the time-limited role of the hippocampus is to serve as a locus in memory processing in a wider consolidation circuit that includes bidirectional cortico-hippocampal interactions. Nadel and Bohbot (2001) inferred a process of consolidation from retrograde amnesia, but do not see consolidation as a transfer of memory from the hippocampus to other areas. Rather, interactions between systems preserve their respective specializations. All of these heuristic proposals have points of contact within the nSTART model.

Building on the critique of McClelland et al. (1995) given in Grossberg & Merrill (1996), the nSTART model embodies a quite different proposal of hippocampal function than that of the MeClelland et al. (1995) model of consolidation. The nSTART model avoids the representation problem because neocortex and hippocampus learn different things. It avoids the learning rate problem because neocortex can learn as fast as sensory inputs and modulatory processes allow. It avoids the real-time learning problem because the fast real-time incremental learning that ART, CogEM, and START allow does not require unrealistic learning mechanisms such as interleaving, and works well in environments whose statistics can change unpredictably through time (Carpenter & Grossberg, 1991, 1993; Grossberg, 2003, 2007, 2013; Grossberg & Levine, 1987; Grossberg & Merrill, 1992, 1996; Grossberg & Schmajuk, 1987, 1989).

Additionally, the nSTART model proposes how three basic learning problems are solved: It enables fast motivated attention to be paid to salient objects and events using pathways to and from the amygdala that support conditioned reinforcer and incentive motivational learning (Figs. 2, 4, 5 and 6). It maintains motivated attention for an appropriate duration on salient objects and events using an adaptively-timed cortical-hippocampal-cortical circuit that also inhibits unwanted orienting reactions (Fig. 6). Finally, it prevents premature responses using adaptively-timed cerebellar motor learning (Figs. 2 and 16). Thus, the hippocampal influence on cortical learning is not just a transfer of the same memory trace, but rather the result of interactions between multiple types of learning. An enhanced understanding in nSTART of the role of neurotrophins in the creation and maintenance of memory and the role of attention in the generation of awareness and self-consciousness builds upon this analysis.

### Clinical relevance of BDNF

In line with recent work on the etiology and treatment of neurological diseases such as Alzheimer’s, Parkinson’s, Huntington’s, epilepsy, Rett’s syndrome, and neuropsychiatric disorders such as depression, bipolar, anxiety-related, schitzophrenia, and addiction (Autry & Monteggia, 2012; Hu & Russek, 2008), the nSTART model is consistent with clinical treatments for impaired cognitive function that implicate an important role for BDNF. In clinical applications, the deleterious effects on synaptic and behavioral plasticity associated with low-levels of BDNF may be reversed by exercise (Molteni et al., 2004), a finding with obvious relevance to educational intervention as well. Treatments that include cognitive and physical exercise have been shown to increase BDNF levels and to relieve symptoms (Cotman & Berchtold, 2002). In addition, BDNF levels, low in proportion to the severity of mania and depression, increase with clinical improvement using antidepressants and mood stabilizers (Post, 2007). However, too much excitation can cause problems and require therapies to down-regulate BDNF and related processes (Birnbaum et al., 2004; Koyama & Ikegaya, 2005).

### Mathematical equations and parameters

#### nSTART model overview

nSTART is a real-time neural network with multiple feedforward and feedback connections. On-center off-surround membrane, or shunting, equations with terms for spontaneous decay, input-driven excitation and inhibition, and recurrent excitation and inhibition represent a rate-based approximation to Hodgkin-Huxley dynamics. These equations were integrated over time using the Runge–Kutta 4 method for ODE numerical integration written in MatLab 12.1 running under the Windows 8 operating system on an Intel Quad Core microprocessor. The equations demonstrated the reported qualitative properties over a wide range of parameter choices. Final parameter selection was based on the goal of running all of the simulations using a single set of parameters. Figure 18 shows the mechanistic circuit diagram of the interacting nSTART pathways and processes that were illustrated in Figs. 2 and 7 and qualitatively described above. The equations are formally described below. Table 2 presents all system variables and their initial values as well as the parameters with their values.

The model was tested by simulating data from reinforcement learning experiments, notably classical conditioning experiments. To simplify the model, we use two types of input: *I*_*i*_, *i* ≥ 1, which turns on when the i^th^ CS, CS_*i*_, occurs, and *I*_0_, which turns on when a US occurs. *I*_*i*_ activates the *i* ^*t*h^ sensory representation *S*_*i*_. Another population of cells *A* represents a drive representation in the amygdala. It receives a combination of sensory, reinforcement, and homeostatic (or drive) stimuli. Reinforcement learning, emotional reactions, and motivated attention decisions are controlled by *A*. During conditioning, presentation of a CS (*I*_1_) before a US (*I*_0_) causes activation of sensory cortical activity *S*_*i*_ followed by activation of *A*. Such pairing strengthens the adaptive weight, or long-term memory trace, in the modifiable synapses from *S*_*i*_ to *A*, and converts *CS*_*i*_ into a conditioned reinforcer. Conditioned reinforcers hereby acquire the power to activate *A* via the conditioning process. These and other learning and performance processes of the nSTART model are defined by the following equations and parameters.

#### Sensory cortex and thalamus

##### Sensory cortical dynamics

Cell activity, or voltage *V*(*t*), *in vivo* can be represented by the membrane, or shunting, equation:

1 $$ C\frac{d}{dt}V=\left({V}^{+}-V\right){g}^{+}+\left({V}^{-}-V\right){g}^{-}+\left({V}^p-V\right){g}^p, $$

where *C* is capacitance; the constants *V* ^+^, *V* ^−^, and *V* ^*p*^are excitatory, inhibitory, and passive saturation points of *V*, respectively; and *g* ^+^, *g* ^−^, and *g* ^*p*^ are conductances that can be changed by inputs (Grossberg, 1968b; Hodgkin, 1964). In the model equations, *V* is replaced with a symbol that represents the activity of a particular cell (population) in the network. A basic processing unit in the model is a network of shunting neurons that interact within a feedforward and/or feedback on-center off-surround network whose shunting dynamics contrast-normalize its cell activities (Grossberg, 1973, 1980). These networks also have a total activity with an upper bound that tends to be independent of the number of active cells.

The activity *S*_*i*_ of the i^th^ sensory cortical cell (population) obeys:

2 $$ \frac{d}{dt}{S}_i=-15{S}_i+{\beta}_S\left(1-{S}_i\right)\left({I}_i+{f}_S\left({S}_i\right)\left(1+{O}_i\right)\right){S}_{mi}-15{S}_i{\displaystyle \sum_{k\ne i}}\;{f}_S\left({S}_k\right)\left(1+{O}_k\right). $$

The inputs *I*_*i*_ are turned on and off by presentation and termination of a CS input (*I*_1_) or US input (*I*_0_) over time. Term − 15*S*_*i*_ describes passive decay of activity *S*_*i*_. Term *β*_*S*_(1 − *S*_*i*_)(*I*_*i*_ + *f*_*S*_(*S*_*i*_)(1 + *O*_*i*_))*S*_*mi*_ describes excitatory interactions in response to input *I*_*i*_, notably the recurrent on-center excitatory feedback signal *f*_*S*_(*S*_*i*_) from population *S*_*i*_ to itself (Eq. 4), the top-down modulatory attentional input *O*_*i*_ from orbitofrontal cortex, and the habituative transmitter *S*_*mi*_ that depresses these excitatory interactions in an activity-dependent way (Eq. 6). Excitation is scaled by parameter *β*_*S*_. Due to the shunting term *β*_*S*_(1 − *S*_*i*_) in*β*_*S*_(1 − *S*_*i*_)(*I*_*i*_ + *f*_*S*_(*S*_*i*_)(1 + *O*_*i*_))*S*_*mi*_, activity *S*_*i*_ can continue to grow until it reaches the excitatory saturation point, which is set to 1 in Eq. 2. Term $-15{S}_i{\displaystyle \sum_{k\ne i}}\;{f}_S\left({S}_k\right)\left(1+{O}_k\right)$ describes lateral inhibition of *S*_*i*_ by competitive feedback signals *f*_*S*_(*S*_*k*_) from the off-surround of other sensory cortical activities *S*_*k*_, *k* ≠ *i*, modulated by the corresponding top-down orbitofrontal signal *O*_*k*_. Due to the excitatory feedback signals, a brief CS input (*I*_1_) gives rise to a sustained STM activity *S*_*i*_ which can remain sensitive to the balance of signals across the network due to its shunting off-surround, notably by competition from activation in response to the US input (*I*_0_).

The dynamics of (sensory cortical)-to-(orbitofrontal cortical) circuits are modeled (Fig. 2). For simplicity, activity levels of thalamus (*T*_*i*_) and sensory cortex (*S*_*i*_) are lumped into a single representation:

3 $$ {T}_i\equiv {S}_i. $$

With this convention in mind, simulation results may interchangeably mention thalamo-cortical or cortico-cortical connectivity, as required by a given context.

##### Signal functions in recurrent on-center off-surround shunting network

The signal function *f*_*S*_(*S*_*k*_) in Eq. 2 is a particularly simple faster-than-linear signal function, one that is half-wave-rectified, and then linear above an output threshold: (Grossberg, 1973):

4 $$ {f}_S\left({S}_k\right)={\left[{S}_i-0.02\right]}^{+} \equiv max\left({S}_i-0.02,0\right), $$

where 0.02 is the threshold value that must be exceeded for the signal to become positive. Faster-than-linear signal functions tend to suppress noise while contrast-enhancing the most active cell activity and making winner-take-all choices in networks such as (Eq. 2), as proved in Grossberg (1973).

##### Habituative transmitter gates

Habituative transmitters such as *S*_*mi*_ in (Eq. 2) tend to obey equations of the following general form (Grossberg 1968b, 1972, 1980):

5 $$ \frac{d}{dt}{N}_{mi}=0.5\left(1-{N}_{mi}\right)-2.5{f}_N\left({N}_i\right){N}_{mi}. $$

The amount of neurotransmitter *N*_*mi*_ in (Eq. 5) accumulates, scaled by a factor of 0.5, up to a limit of 1 due to the accumulation term 1 − *N*_*mi*_, and is inactivated, or habituates, by the gated release term − 2.5*f*_*N*_(*N*_*i*_)*N*_*mi*_, whereby *N*_*mi*_ is inactivated by mass action at a rate proportional to the product of an excitatory signal*f*_*N*_(*N*_*i*_) from either sensory cortex (Eq. 2) or orbitofrontal cortex (Eq. 7), and the amount *N*_*mi*_ of available transmitter. These modulators are similar to those in the habituative transmitter spectrum for hippocampal cells (Eq. 22).

In particular, *S*_*mi*_ in (Eq. 2) obeys:

6 $$ \frac{d}{dt}{S}_{mi}=0.5\left(1-{S}_{mi}\right)-2.5\left({I}_i+{f}_S\left({S}_i\right)\left(1+{O}_i\right)\right){S}_{mi}. $$

*S*_*mi*_ accumulates up to a limit of 1 due to the accumulation term 0.5(1 − *S*_*mi*_), and is inactivated by mass action at a rate proportional to the product of (*I*_*i*_ + *f*_*S*_(*S*_*i*_)(1 + *O*_*i*_), the excitatory term in Eq. 2 that the transmitter gates, and the amount of available transmitter *S*_*mi*_. A similar transmitter equation acts within orbitofrontal cortex (Eq. 13).

#### Orbitofrontal cortex, category learning, and incentive motivational learning

##### Orbitofrontal cortical dynamics

The activity *O*_*i*_ of the i^th^ orbitofrontal cortical cell (population) obeys:

7 $$ \frac{d}{dt}{O}_i=-10{O}_i+{\beta}_O\left(2-{O}_i\right)\left(\left({f}_S\left({S}_i\right)+0.03\right)0.0625{w}_{Si}\left(A{w}_{Ai}+10H{w}_{Hi}+800{B}_{Ci}\right)+0.75{O}_i\right){O}_{mi}-10{O}_i{\displaystyle \sum_{k\ne i}}{O}_k $$

In (7), a phasic input from sensory cortex (*f*_*S*_(*S*_*i*_), Eq. 2), plus a tonic activity of 0.03 (see *f*_*S*_(*S*_*i*_) + 0.03), is modulated by inputs from the amygdala (*A*, Eq. 14), hippocampus (*H*, Eq. 16), and orbitofrontal BDNF (*B*_*Oi*_, Eq. 12). In addition, a recurrent self-excitatory feedback signal (*O*_*i*_) supports persistence of orbitofrontal activity after the external sensory input is turned off and *f*_*S*_(*S*_*i*_) decays to 0. As in Eq. 2, there is a passive decay term − 10*O*_*i*_, an excitatory shunting on-center term *β*_*O*_(2 − *O*_*i*_)((*f*_*S*_(*S*_*i*_) + 0.03)0.0625*w*_*Si*_(*Aw*_*Ai*_ + 10*Hw*_*Hi*_ + 800*B*_*Oi*_) + 0.75*O*_*i*_)*O*_*mi*_ that can increase up to 2, its saturation point, an activity-dependent habituative transmitter gate *O*_*mi*_ of excitatory cortical interactions (Eq. 7), and a shunting off-surround inhibitory term $-10{O}_i{\displaystyle \sum_{k\ne i}}\;{o}_k$ that enables contrast normalization. Adaptive weights, or LTM traces, *w*_*Si*_, *w*_*Ai*_, and *w*_*Hi*_ (see Eqs. 8, 9, 10, and 11) gate the inputs *f*_*S*_(*S*_*i*_), *A*, and *H*, respectively. An excitatory gain of 10 multiplies *H* and of 800 multiplies *B*_*Oi*_.

##### Cortical category learning and incentive motivational learning

The learned adaptive weights to the orbitofrontal cortex all obey an outstar learning law (Grossberg, 1980), as described above. The weights from amygdala and hippocampus (*w*_*Ai*_ and *w*_*Hi*_, respectively) supply incentive motivational support for cortico-cortical category learning by *w*_*Si*_. All weights obey the general form:

8 $$ \frac{d}{dt}{w}_{Mi}=4\left({f}_M\left({M}_i\right)+{B}_{Oi}\right)\left(-{w}_{Mi}+2{O}_i\right), $$

where *M* = *S*, *A*, or *H*, depending on the context.

Learned adaptive weights from sensory cortex to orbitofrontal cortex obey:

9 $$ \frac{d}{dt}{w}_{Si}=4\left({f}_S\left({S}_i\right)+{B}_{Oi}\right)\left(-{w}_{Si}+2{O}_i\right), $$

where learning is gated on and off by a sampling signal *f*_*s*_(*S*_*i*_) + *B*_*Oi*_ that is the sum of the sensory cortical signal *f*_*S*_(*S*_*i*_) (Eq. 4), and the orbitofrontal BDNF*B*_*Oi*_ (Eq. 12).The sampling signal’s size determines the rate at which weight *w*_*Si*_ approaches twice the orbitofrontal activity *O*_*i*_ (Eq. 7) via term − *w*_*Si*_ + 2*O*_*i*_.

Learned adaptive weights from amygdala to orbitofrontal cortex obey:

10 $$ \frac{d}{dt}{w}_{Ai}=4\left(0.1A+{B}_{Oi}\right)\left(-{w}_{Ai}+2{O}_i\right) $$

and from hippocampus to orbitofrontal cortex obey:

11 $$ \frac{d}{dt}{w}_{Hi}=4\left(0.5H+{B}_{Oi}\right)\left(-{w}_{Hi}+2{O}_i\right). $$

##### Orbitofrontal BDNF

Orbitofrontal BDNF *B*_*Oi*_ is time-averages hippocampal signals *H* that are gated by learned weights *w*_*Hi*_ with an excitatory gain 3.125:

12 $$ \frac{d}{dt}{B}_{Oi}=-{B}_{Oi}+3.125H{w}_{Hi}. $$

##### Habituative transmitter gates in orbitofrontal cortex

Activity-dependent habituative neurotransmitters, or postsynaptic sites, *O*_*mi*_ that influence orbitofrontal cortical activity obey a specialized version of (Eq. 5):

13 $$ \frac{d}{dt}{O}_{mi}=0.5\left(1-{O}_{mi}\right)-2.5\left(\left({f}_S\left({S}_i\right)+0.03\right)0.0625{w}_{Si}\left(A{w}_{Ai}+10H{w}_{Hi}+800{B}_{Ci}\right)+0.75{O}_i\right){O}_{mi}, $$

that accumulates to a maximum value of 1 at rate 0.5 via term 0.5(1 − *O*_*mi*_), and habituates, or is inactivated, at rate − 2.5((*f*_*S*_(*S*_*i*_) + 0.03)0.0625*w*_*Si*_(*Aw*_*Ai*_ + 10*Hw*_*Hi*_ + 800*B*_*Ci*_) + 0.75*O*_*i*_) by the on-center input term in (Eq. 7).

#### Amygdala and conditioned reinforcer learning

##### Amygdala drive representation dynamics

The amygdala activity *A* of the drive representation obeys:

14 $$ \frac{d}{dt}A=-20A+{\beta}_A\left(10-A\right){\displaystyle \sum_i}\;{f}_S\left({S}_i\right){F}_i. $$

Activity *A* passively decays via term − 20*A*. Term ${\beta}_A\left(10-A\right){\displaystyle \sum_i}\;{f}_S\left({S}_i\right){F}_i$ describes the sum of excitatory signals*f*_*S*_(*S*_*i*_)from the i^th^ sensory representation to *A*, gated by the conditioned reinforcer adaptive weights *F*_*i*_ (Eq. 15). This sum can increase *A* until it reaches the saturation term 10 that is determined by term (10 − *A*). Adaptive weight*F*_*i*_ determines how well *S*_*i*_ can activate *A*, and thus the extent to which the *i* ^th^ CS has become a conditioned reinforcer through learning. Because *F*_*i*_ multiplies*f*_*S*_(*S*_*i*_), a large *S*_*i*_ will have a negligible effect on *A* if *F*_*i*_ is small, and a large effect on *A* if *F*_*i*_ is large. The US LTM trace *F*_0_ is fixed at a relatively large value to enable the US to activate *A* via *S*_0_and to thereby drive conditioned reinforcer learning when a CS is also active. The CS LTM trace *F*_1_ is initially set to one tenth of the US value to prevent the CS from significantly activating *A* before conditioning takes place.

##### Conditioned reinforcer learning

Each adaptive weight *F*_1_ obeys an outstar learning law:

15 $$ \frac{d}{dt}{F}_1=0.5{f}_S\left({S}_i\right)\left(-{F}_1+0.2A\right). $$

Learning by *F*_1_ is turned on and off by the sampling signal 0.5*f*_*S*_(*S*_*i*_), whose size determines the rate at which*F*_1_ time-averages 0.2*A*. Activity *F*_1_ can increase or decrease during learning, hence both long-term potentiation (LTP) and long-term depression (LTD) can occur. To represent the non-learned response to the US, *F*_0_ is held constant at 0.5.

#### Hippocampus and adaptively timed learning

##### Adaptively-timed hippocampal learning

As noted above, the hippocampus delivers adaptively timed signals *H* to the orbitofrontal cortex that can maintain its activity for a duration that can span the trace interval; see Eq. 6. The hippocampus hereby activates an adaptively-timed incentive motivational pathway in cases when the amygdala cannot. The spectral timing process embodies several processing steps.

##### Adaptively-timed hippocampal activity

Activity *H* in the hippocampus obeys:

16 $$ \frac{d}{dt}H=-15H+{\beta}_H\left(2-H\right)\left(0.625R+0.5{B}_H\right). $$

Term − 15*H* represents passive decay. The excitatory term is scaled by the excitatory gain *β*_*H*_ and bounded by 2, due to the shunting term *β*_*H*_(2 − *H*). The two sources of excitatory input are the adaptively timed input *R* (Eq. 17) and the total BDNF input *B*_*H*_ (Eq. 27), each with its own gain term.

##### Adaptively-timed population output signal

The adaptively timed signal *R* is a population response:

17 $$ R={\displaystyle \sum_{i,j}}\;{h}_{ij} $$

that sums over multiple individually timed signals

18 $$ {h}_{ij}=8f\left({x}_{ij}\right){y}_{ij}{z}_{ij} $$

that are defined below. None of the signals *h*_*ij*_ individually can accurately time the ISI between a CS and US. The entire population response in (Eq. 17) can do so using a “spectrum” of differently timed cells, leading to the term “spectral timing” for this kind of learning (Grossberg and Merrill, 1992, 1996; Grossberg and Schmajuk, 1989).

##### Activation spectrum

Model simulations use the simplest embodiment of spectrally-timed learning. A more detailed biochemical model is given using Ca^++^-modulated learning by a spectrum of metabotropic glutamate receptor (mGluR) cell sites in Fiala, Grossberg, and Bullock (1996), which shows how mGluR dynamics can span such long time intervals.

Spectrally timed learning can be initiated when an input signal *f*_*S*_(*S*_*i*_) (Eq. 4) from a sensory cortical representation (Eq. 2) activates a population of hippocampal cell sites with activities *x*_*ij*_ that activate the next processing stage via sigmoidal signals:

19 $$ f\left({x}_{ij}\right)=\frac{x_{ij}^8}{0.01^8+{x}_{ij}^8}. $$

Activities *x*_*ij*_ react at a spectrum of rates:

20 $$ \frac{d}{dt}{x}_{ij}={r}_j\left(-{x}_{ij}+\left(1-{x}_{ij}\right){f}_S\left({S}_i\right)\right), $$

with rates *r*_*j*_ ranging from 0.171 (fast) to 0.016 (slow) defined by:

21 $$ rj=5.125/\left(0.0125+15\left(j+1\right)\right), $$

for j = 1 to 20.

##### Habituative transmitter spectrum

Each spectral activation signal *f*(*x*_*ij*_) is gated by a habituative chemical transmitter, or postsynaptic response, *y*_*ij*_ that obeys:

22 $$ \frac{d}{dt}{y}_{ij}=0.5\left(1-{y}_{ij}\right)-10f\left({x}_{ij}\right){y}_{ij}. $$

As in Eq. 5, *y*_*ij*_ accumulates to 1 via term (1 − *y*_*ij*_) at rate 0.5, and habituates, or inactivates, due to a mass action interaction with signal *f*(*x*_*ij*_), via the gated release term− 10*f*(*x*_*ij*_)*y*_*ij*_. The different rates *r*_*j*_ that activate each *x*_*ij*_ cause the habituative transmitters *y*_*ij*_ to become habituated at different rates as well. The family of curves*y*_*ij*_,*j* = 1, 2, …, 20, is called a habituation spectrum.

##### Gated signal spectrum and time cells

Each signal *f*(*x*_*ij*_)interacts with *y*_*ij*_ via mass action to generate a net output signal from its population of cell sites that obeys:

23 $$ {g}_{ij}\equiv {\left[f\left({x}_{ij}\right){y}_{ij}-0.03\right]}^{+}\equiv \max \left(f\left({x}_{ij}\right){y}_{ij}-0.03,0\right). $$

Each gated signal *g*_*ij*_ has a different rate of growth and decay, thereby generating a unimodal function of time that achieves its maximum value *M*_*ij*_ at time *T*_*ij*_, where *T*_*ij*_ is an increasing function of *j*, and *M*_*ij*_ is a decreasing function of *j*. Taken together, all the functions *g*_*ij*_ define the gated signal spectrum in Fig. 11c. This timed spectrum is the basis of adaptively timed learning over an extended time interval that can range from hundreds of milliseconds to several seconds, with each *g*_*ij*_ acting as the sampling signal for its part of the adaptively timed spectrum.

##### Spectral learning law

Each adaptive weight *z*_*ij*_ in the spectrum obeys an outstar learning law:

24 $$ \frac{d}{dt}{z}_{ij}=2{g}_{ij}\left(-{z}_{ij}+2N\right). $$

In Eq. 24, *g*_*ij*_ is a sampling signal that determines the rate with which *z*_*ij*_ samples a transient Now Print signal *2N* (Eq. 25) that is derived from amygdala activity *A* in Eq. 14. Each *z*_*ij*_ changes by an amount that reflects the degree to which the curves *g*_*ij*_ and *N* have simultaneously large values through time. If *g*_*ij*_ is large when *N* is large, then *z*_*ij*_ increases in size. If *g*_*ij*_ is large when *N* is small, then *z*_*ij*_ decreases in size. Since the different *g*_*ij*_ peak at different times, each *z*_*ij*_ responds to *N* to different degrees.

The Now Print signal *N* obeys:

25 $$ N={\left[A-E-0.04\right]}^{+}\equiv max\left(A-E-0.04,0\right), $$

where *E* is a feedforward inhibitory interneuron that obeys:

26 $$ \frac{d}{dt}E=40\left(-E+A\right). $$

The inhibitory interneuronal activity *E* in (26) time-averages the amygdala activity *A* at rate 40. Its activity hereby lags behind that of *A*. The difference (*A* − *E*) in (25) may thus be activated by any sufficiently rapid increase in A. Either a US, or a CS that has become a conditioned reinforce, can cause such a rapid increase, and thereby activate *N*, and thus learning of any adaptive weight *z*_*ij*_ whose sampling signal *g*_*ij*_ is sufficiently large at such a time.

An important property of *N* is that it increases in amplitude, but not significantly in duration, in response to larger inputs *A*. Thus learning can be faster in response to stronger rewards, but the timing of a conditioned response does not significantly change, as in the data and our simulations thereof (Fig. 8).

##### Doubly-gated signal spectrum

Each long-term memory trace *z*_*ij*_ learns to a different degree. Each *z*_*ij*_ also gate the signals *g*_*ij*_ in order to generate a twice-gated output signal *h*_*ij*_ (Eq. 18) from each of the differently timed cell sites. Comparing the signals *h*_*ij*_ in Fig. 11d with the *g*_*ij*_ in Fig. 11c shows how adaptively timed learning changes the relative strength of each spectral output. When all the *h*_*ij*_ are added together to generate the population output *R* in (Eq. 17), accurate adaptively timing is achieved.

##### Hippocampal BDNF

Production of hippocampal BDNF*B*_*H*_ is a time average of 25 times its adaptively timed population signal *R* (Eq. 17), scaled by a reaction rate of 2:

27 $$ \frac{d}{dt}{B}_H=2\left(-{B}_H+25R\right). $$

Hippocampal BDNF in the model extends hippocampal activation, and thus the incentive motivational support that it supplies to cortico-cortical learning during a memory consolidation period after the CS and US inputs terminate.

#### Pontine nuclei

##### Final common path for conditioned output

Output signals from the amygdala *A* (Eq. 14) and the CS-activated orbitofrontal cortical representation *O*_1_ (Eq. 7) to the pons combine to form a common final path that is used in the model as a signal that generates a behavioral CR further downstream:

28 $$ P=A+{O}_1. $$
